# Discovery and Synthesis of a Pyrimidine-Based Aurora Kinase Inhibitor to Reduce Levels of MYC Oncoproteins

**DOI:** 10.1021/acs.jmedchem.0c01806

**Published:** 2021-05-19

**Authors:** Ya-Hui Chi, Teng-Kuang Yeh, Yi-Yu Ke, Wen-Hsing Lin, Chia-Hua Tsai, Wan-Ping Wang, Yen-Ting Chen, Yu-Chieh Su, Pei-Chen Wang, Yan-Fu Chen, Zhong-Wei Wu, Jen-Yu Yeh, Ming-Chun Hung, Mine-Hsine Wu, Jing-Ya Wang, Ching-Ping Chen, Jen-Shin Song, Chuan Shih, Chiung-Tong Chen, Chun-Ping Chang

**Affiliations:** † Institute of Biotechnology and Pharmaceutical Research, National Health Research Institutes, Zhunan 35053, Taiwan; ‡ Graduate Institute of Biomedical Sciences, China Medical University, Taichung 40402, Taiwan; § Department of Chemistry, Chung Yuan Christian University, Taoyuan 320314, Taiwan

## Abstract

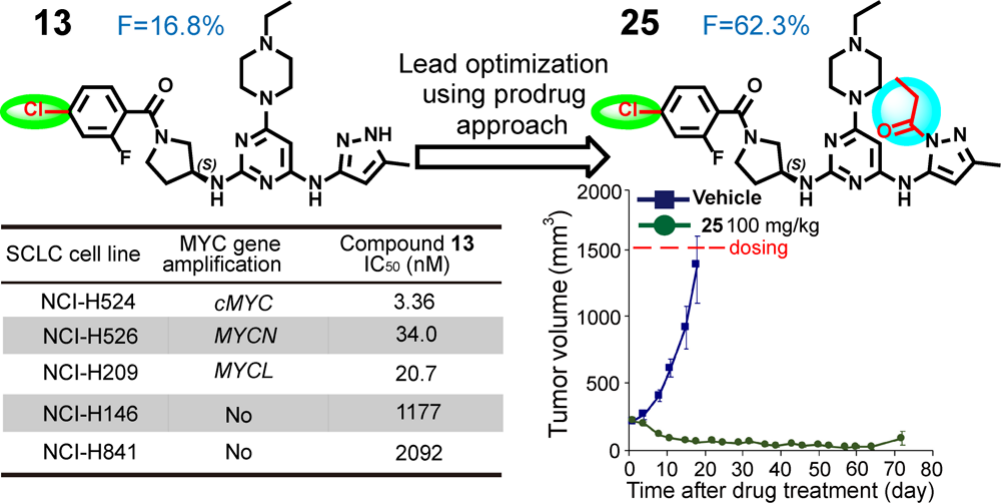

The A-type Aurora kinase is upregulated in many human cancers, and it stabilizes MYC-family oncoproteins, which have long been considered an undruggable target. Here, we describe the design and synthesis of a series of pyrimidine-based derivatives able to inhibit Aurora A kinase activity and reduce levels of cMYC and MYCN. Through structure-based drug design of a small molecule that induces the DFG-out conformation of Aurora A kinase, lead compound **13** was identified, which potently (IC_50_ < 200 nM) inhibited the proliferation of high-MYC expressing small-cell lung cancer (SCLC) cell lines. Pharmacokinetic optimization of **13** by prodrug strategies resulted in orally bioavailable **25**, which demonstrated an 8-fold higher oral AUC (*F* = 62.3%). Pharmacodynamic studies of **25** showed it to effectively reduce cMYC protein levels, leading to >80% tumor regression of NCI-H446 SCLC xenograft tumors in mice. These results support the potential of **25** for the treatment of *MYC*-amplified cancers including SCLC.

## Introduction

Deregulation of MYC-family oncogenes (*i.e.*, *cMYC*, *MYCN*, and *MYCL*) is associated with a poor prognosis and unfavorable survival of cancer patients.^1^ Amplification of *MYC*-family oncogenes has been observed in 28% cancers in The Cancer Genome Atlas (TCGA).^2^ Sustained MYC-family protein levels can initiate tumor formation, accelerate tumor progression, and help in tumor maintenance. Many MYC-driven metabolic changes such as glycolysis and glutaminolysis support the increased need of nucleic acids, proteins, and lipids during rapid cell proliferation.^3,4^ Despite the pivotal role of MYC in normal tissue regeneration,^5,6^ several murine-based studies have supported MYC as a potential therapeutic target for cancers. For example, a conditional transgenic mouse model for MYC-induced tumorigenesis demonstrated that brief inactivation of *cMYC* was sufficient to elicit sustained regression of transplanted osteogenic sarcoma cells,^7^ and knockdown of *cMYC* in glioma cancer stem cells reduced proliferation with concomitant cell cycle arrest and increased apoptosis, whereas nonstem glioma cells displayed limited dependence on MYC expression for survival and proliferation.^8^

MYC has long been considered “undruggable” by small-molecule inhibitors due to its lack of enzymatic activity and an accessible affinity pocket. Nevertheless, alternative approaches toward the modulation of MYC oncogenic functions *via* indirect strategies have been extensively investigated.^9^ For example, use of Omomyc, a 90 amino acid dominant-negative form of MYC, which competes with cellular MYC, prevented formation of MYC/MAX heterodimers and thus, transcriptional activation of a specific set of genes.^10^ The demonstrated antitumor efficacy of the Omomyc miniprotein in several preclinical mouse models has paved the way to clinical trials.^11,12^ MYC transcription was downregulated by the small-molecule bromodomain inhibitor JQ1, which showed potent anticancer effects both *in vitro* and *in vivo* in multiple hematopoietic cancers and pancreatic ductal adenocarcinoma exhibiting high cMYC.^9,13^ CD532, a kinase inhibitor that induces allosteric conformation change of Aurora A kinase, was found to weaken MYCN–Aurora A interaction, thereby releasing MYCN protein for proteasome degradation, resulting in tumor growth inhibition.^14−18^ Although CD532 lacks drug-like properties due to its short half-life and poor oral bioavailability,^14^ this evidence validates an effective strategy for the targeting of MYC-family oncoproteins.

Several small-molecule inhibitors targeting Aurora kinases have been developed in the last two decades,^19−25^ including inhibitors which bind to the protein at the adenosine triphosphate (ATP) binding site which contains a DFG (Asp–Phe–Gly) motif that could adopt two different conformations, the active DFG-in and the inactive DFG-out states. In the DFG-out conformation, the Asp and Phe of the DFG motif of the activation loop swap positions, resulting in the formation of a new allosteric pocket and better enzymatic selectivity.^26^ Using a typical DFG-in inhibitor scaffold of Aurora A, Martin *et al.* discovered that induced-dipole forces along the Ala273 side chain alter the charge distribution of the DFG backbone, leading to DFG-out inhibitors that are highly potent for Aurora A.^27^ We applied this concept to structure-based drug design (SBDD) guided with enzymatic assays and western blot analyses and identified a novel class of 6-methyl-*N*^4^-(5-methyl-1*H*-pyrazol-3-yl)-*N*^*2*^-(pyrrolidin-3-yl) pyrimidine-2,4-diamine derivatives (**A**), which potentially induce a flip in the DFG activation loop of Aurora A, resulting in reduced levels of cMYC and MYCN. Pharmacokinetic (PK) optimization using prodrug strategies for the lead compound **13** resulted in **25**, which demonstrated sufficient oral bioavailability and led to >80% regression of a *cMYC*-amplified small-cell lung cancer (SCLC) in a xenograft mouse model.

## Results and Discussion

### Chemistry

**Scheme 1:**
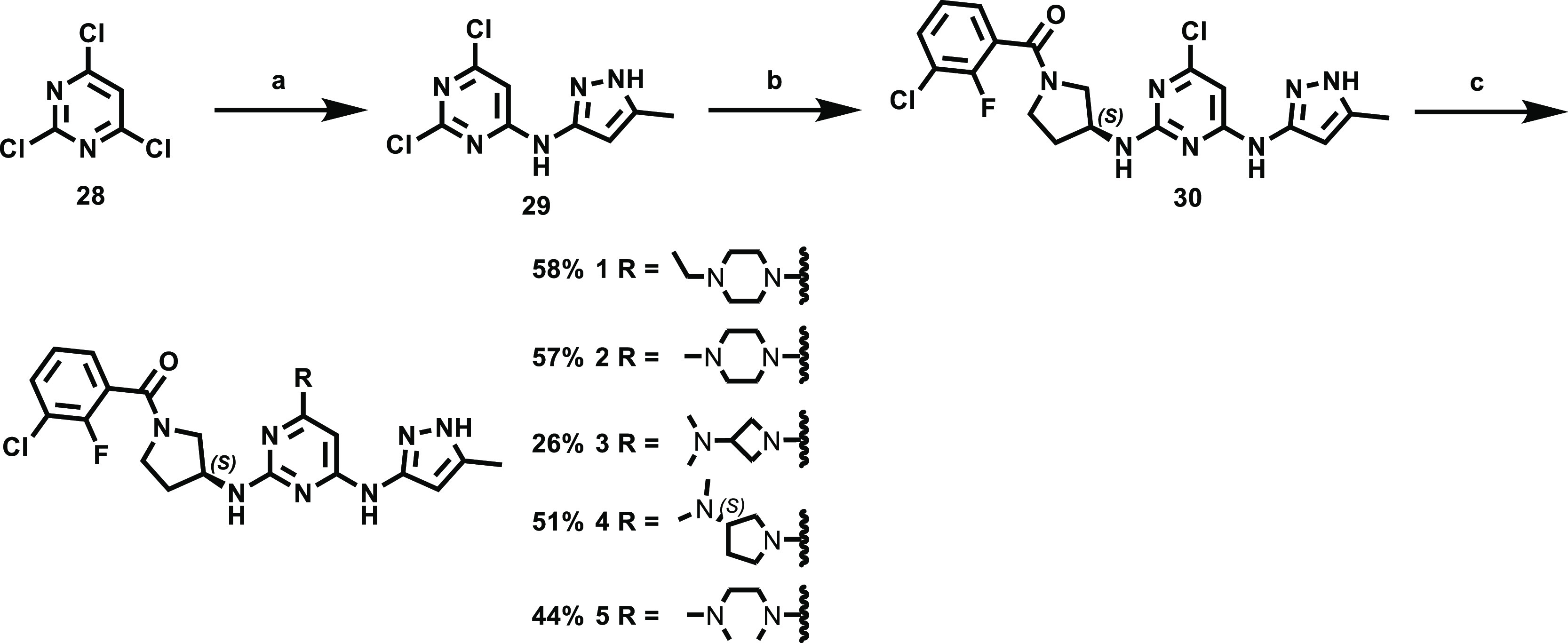
Synthesis of Pyrimidine Derivatives with a Variety of Amines Reagents and conditions: (a) 3-amino-5-methylpyrazole, triethylamine, THF, 50 °C, 16 h, 75%; (b) (*S*)-(3-aminopyrrolidin-1-yl) (3-chloro-2-fluorophenyl)methanone, triethylamine, 1-pentanol, 120 °C, 6 h, 51%; (c) various amine, triethylamine, 1-pentanol, 140 °C, 2 h, 26–58%.

**Scheme 2:**
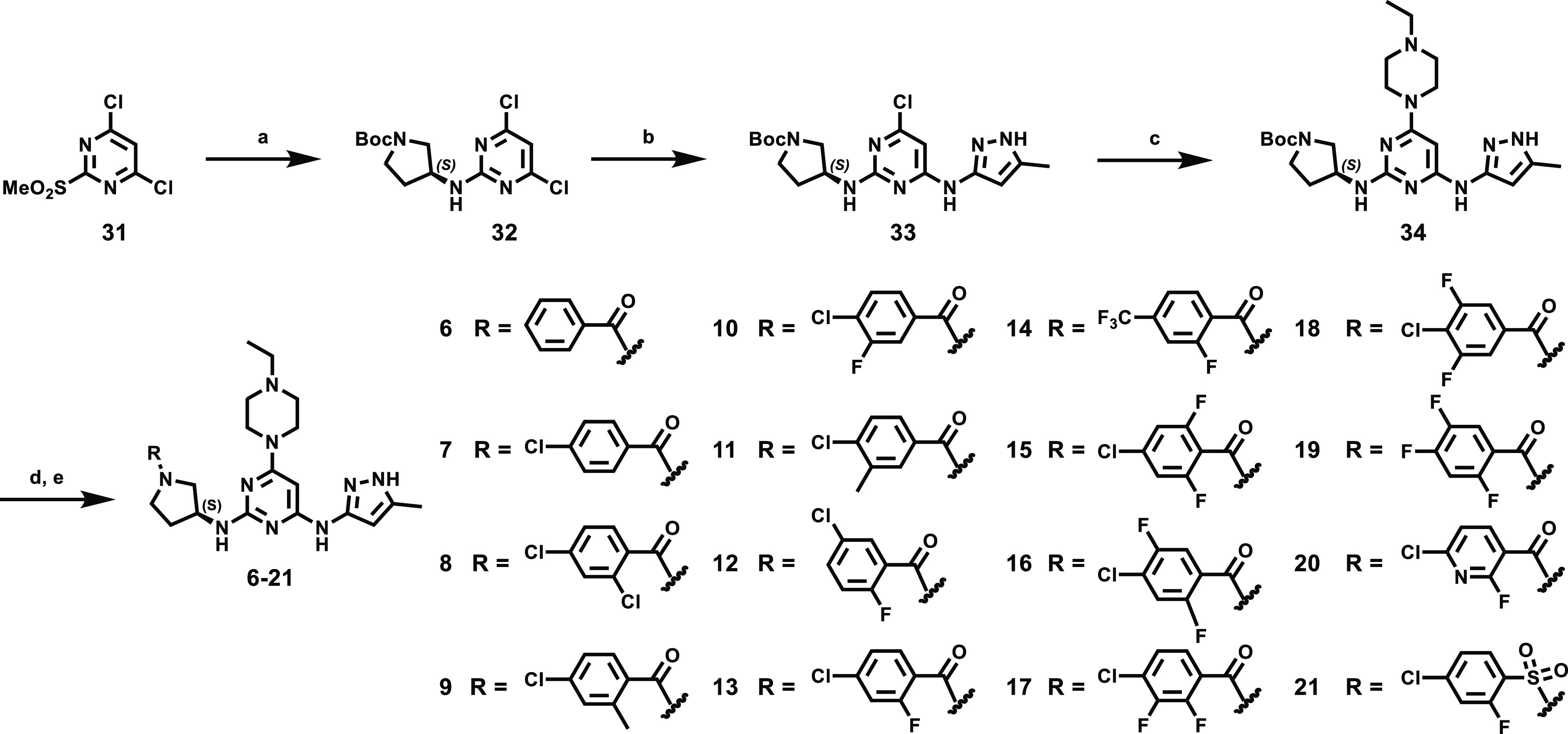
Synthesis of Pyrimidine Derivatives Reagents and conditions: (a) (*S*)-(−)-1-boc-3-aminopyrrolidine, triethylamine, THF, −70 °C, 6 h, 56%; (b) 3-amino-5-methylpyrazole, NaI, triethylamine, DMSO, 90 °C, 16 h, 85%; (c) 1-ethylpiperazine, triethylamine, 1-pentanol, 140 °C, 2 h, 84%; (d) 2 *N* HCl in ether, methanol, dichloromethane, 4 h, 99%; (e) various benzoic acid, T3P, triethylamine, DMF, dichloromethane, 16 h, 44–68% or 6-chloro-2-fluoropyridine-3-carboxylic acid, T3P, triethylamine, DMF, dichloromethane, 16 h, 46% (**20**) or 4-chloro-2-fluorobenzenesulfonyl chloride, triethylamine, dichloromethane, rt, 4 h, 85% (**21**).

**Scheme 3:**
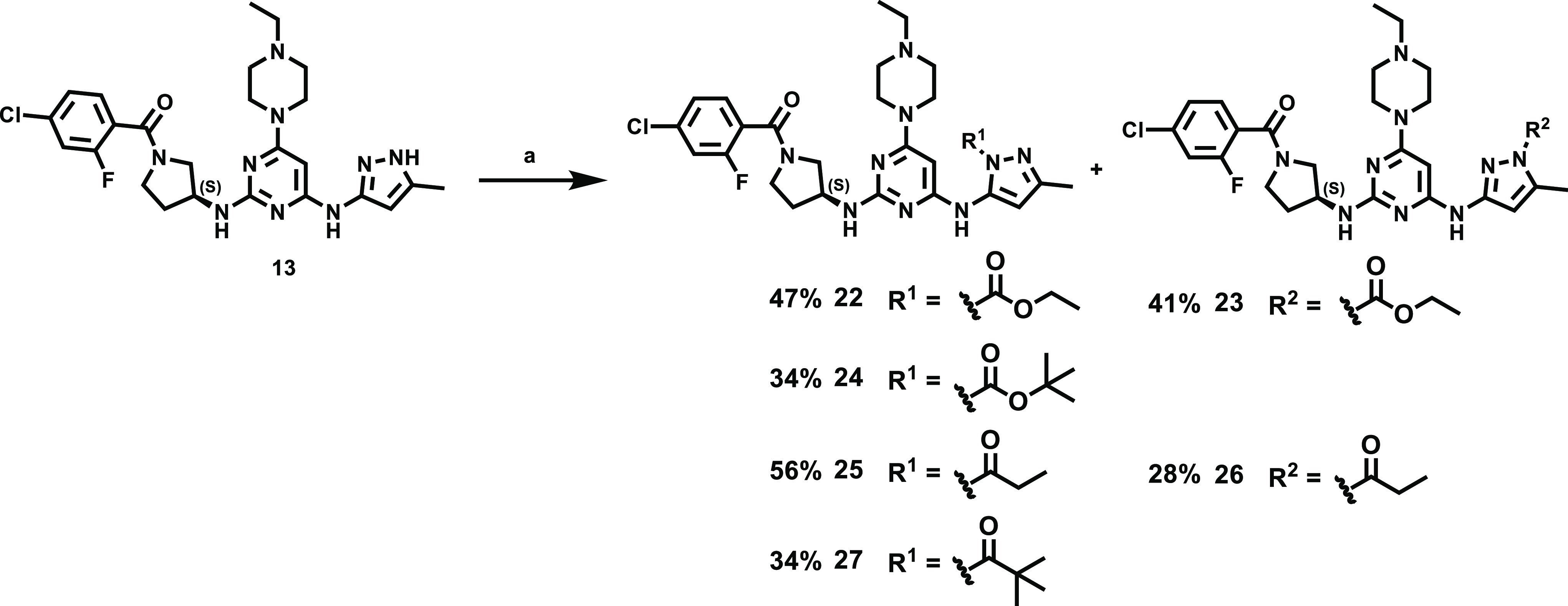
Synthesis of Compound **13** Prodrugs Reagents and conditions: (a) various anhydride or dicarbonate, 1,4-dioxane, 140 °C, 30 min.

Compounds **1**–**21** (Table 1) were prepared according to the general synthetic method depicted in Schemes 1 and 2. A substitution reaction of compound **28** with 3-amino-5-methylpyrazole in THF gave the desired compound **29**, which was converted to the corresponding compound **30** by the reaction with (*S*)-(3-aminopyrrolidin-1-yl) (3-chloro-2-fluorophenyl)methanone in 1-pentanol at 120 °C. Finally, compound **30** was reacted with 1-ethyl piperazine in the presence of trimethylamine to give the desired target compound **1** in 58% yield. Likewise, S_N_Ar (nucleophilic aromatic substitution) reactions of compound **30** with a variety of amines gave the corresponding compounds **2**–**5** in moderate yields (26–57%) (Scheme 1). Treatment of 4,6-dichloro-2-(methylsulfonyl)pyrimidine (**31**) with *tert*-butyl (*S*)-3-aminopyrrolidine-1-carboxylate in the presence of triethylamine gave rise to **32** (56%), which was coupled with 5-methyl-1*H*-pyrazol-3-amine in the presence of NaI and triethylamine in DMSO to provide ether **33** in 85% yield (Scheme 2). Coupling of **33** with 1-ethylpiperazine proceeded at 140 °C to afford **34** in 84% yield. Acidification of **34** gave the corresponding hydrochloride amine salt, which in turn was treated with 4-chloro-2-fluorobenzoic acid and propanephosphonic acid anhydride (T3P) in dichloromethane to yield amide **13** in 56% yield over two steps. Similarly, coupling of the hydrochloride amine salt (from **34**) with a selection of differentially substituted benzoic and pyridinecarboxylic acids and sulfonyl chlorides gave the corresponding amide and sulfonamide derivatives **6–21** in moderate to good yields (44–85%). In addition, the regioisomers **25** and **26** (2:1) were obtained at 140 °C in the presence of propionic anhydride in good yield (84%) (Scheme 3). By the same token, compound **13** directly underwent acylation with selected anhydrides or dicarbonates to afford amide prodrug compound **27** and carbamate prodrug compounds **22–24** in moderate to good yields (34–47%, Scheme 3). The purity of all synthesized compounds was established to be at least 95% by HPLC (see the Experimental Section) prior to their utilization in biological assays and animal studies.

**Table 1:**
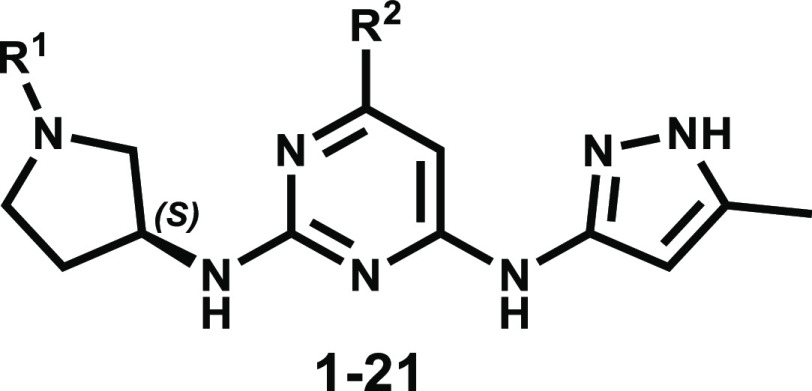

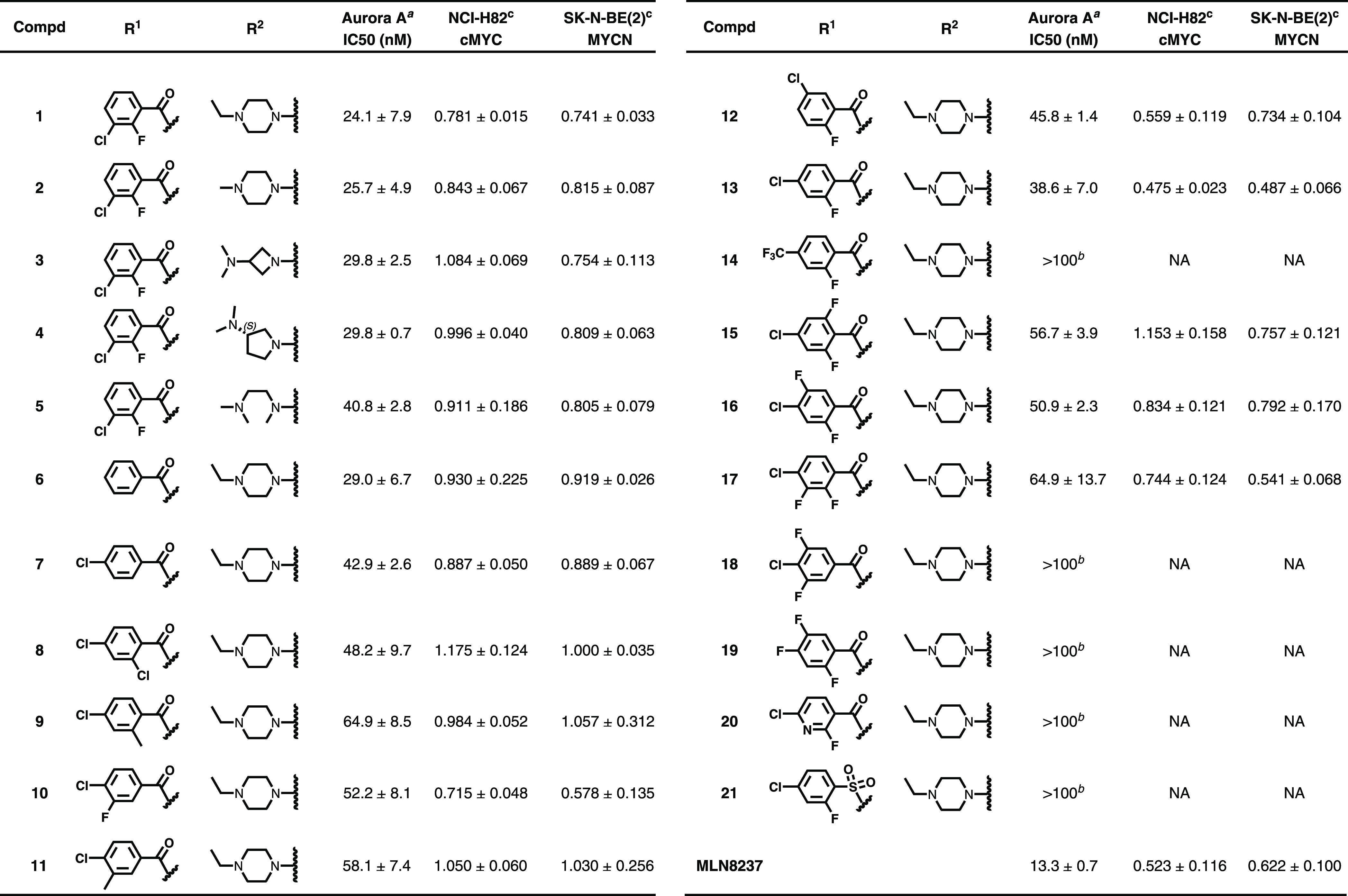
Biological Evaluation of Pyrimidines Derivatives on the Enzymatic Activity of Aurora A and Levels of MYC in *MYC*-Amplified Cancer Cells

a Values are the mean of 2–3 independent determinations ± SD (standard deviation).

b Inhibition < 50% at 100 nM.

c Relative protein level (mean ± SD) of cMYC in NCI-H82 and MYCN in SK-N-BE(2) under 1.0 μM of compound treatment. Experiments were undertaken in triplicate.

### SBDD, Biological Evaluation, and Structure–Activity Relationship

**Figure 1:**
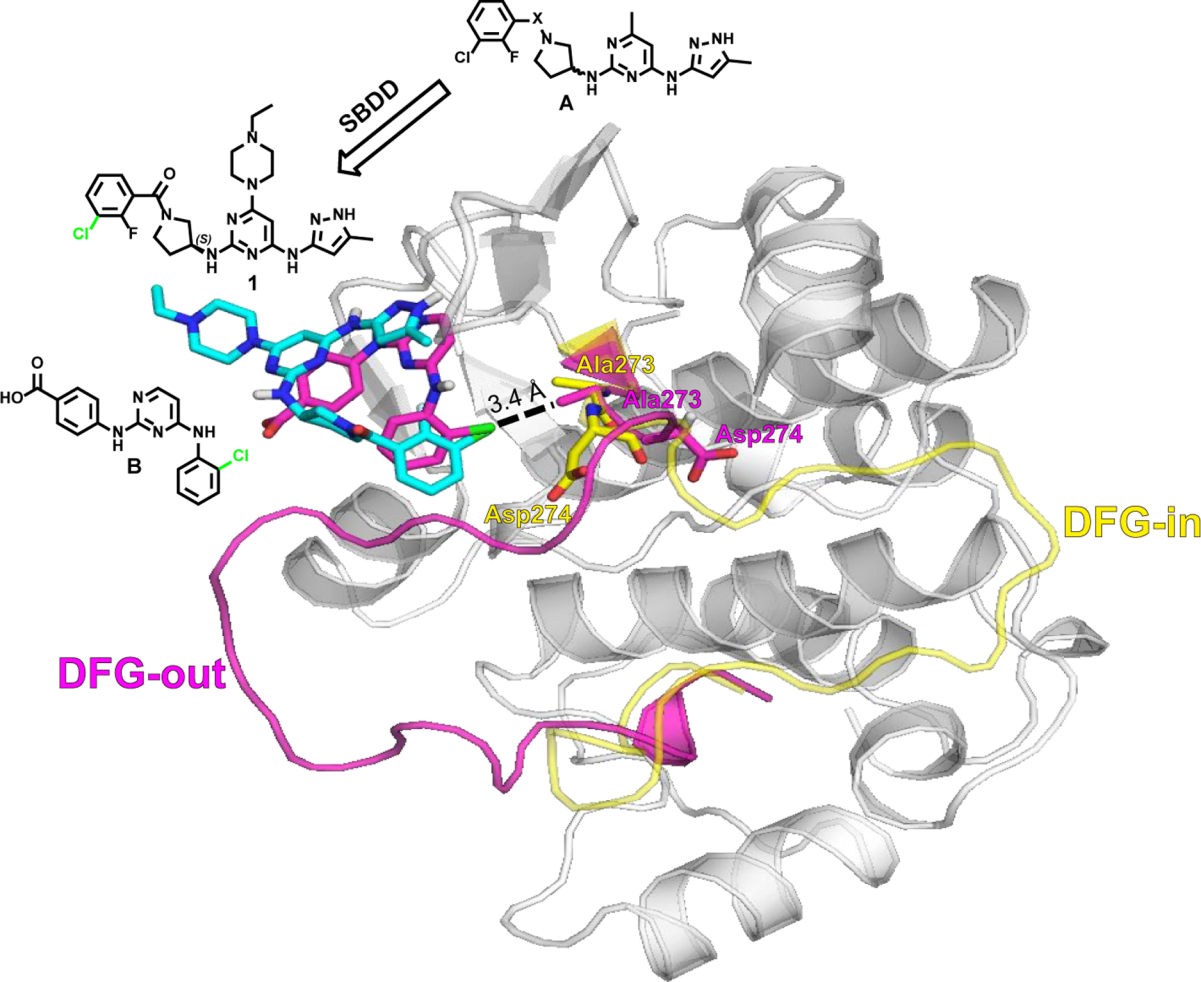
Molecular docking model of structure **A**-derived compound **1** (cyan) aligned with compound **B** (magenta) in complex with Aurora A (PDBID: 3UO6, backbone in gray, DFG-loop in magenta) and compared with an X-ray structure of Aurora A in complex with ATP (PDBID: 5DNR, DFG-loop in yellow). SBDD, structure-based drug design.

Informed by structure modeling, we developed a novel class of 6-methyl-*N*^4^-(5-methyl-1*H*-pyrazol-3-yl)-*N*^2^-(pyrrolidin-3-yl) pyrimidine-2,4-diamine derivatives (structure **A**, Figure 1) as a versatile scaffold for the development of Aurora A kinase inhibitors adopting the DFG-out conformation. Compound **1** was designed through structure modeling of compound **B**, wherein the halogen group establishes an electrostatic dipole–dipole interaction with the methyl group of Ala273, causing the DFG activation loop to flip.^27^ Structural modeling revealed that the chloride substituent of compound **1** aligned well with compound **B** in the same binding pocket of Aurora A, and the distance between the chlorine atom to carbon atom of Ala273 in **1** is similar to **B** (*i.e.*, 3.4 Å) (Figure 1).

The inhibitory potency of the newly synthesized small molecules against Aurora A was first measured in ATP consumption assays using purified Aurora A protein. Aurora A kinase inhibitors capable of inducing a DFG flip have been previously demonstrated to cause degradation of MYC-family oncoproteins in neuroblastoma and SCLC cells.^14,16^ Therefore, compounds that exhibited IC_50_ < 100 nM enzymatic efficacy were subjected to western blot analysis for protein levels of cMYC and MYCN, respectively, in NCI-H82 [a SCLC cell line with *cMYC* amplification] and SK-N-BE(2) [a neuroblastoma cell line with *MYCN* amplification] cells (Table 1 and Supporting Information Figure S1A–C). Western blot analyses showed compound **1** marginally reduced MYCN level at a concentration of 1.0 μM after 24 h (Table 1). Compounds **2–5** bearing modifications to the *R*^2^ water-solubilizing group, such as the acyclic polar group on the 4-position of the pyrimidine ring showed limited ability to reduce levels of the cMYC/MYCN oncoproteins. Derivatization of the 4-position of the ethyl piperazine moiety presumably changed the physiological properties (including solubility, absorption, and metabolic rate) of the compounds but did not affect cMYC/MYCN levels. Compound **6** (lacking substitution on the benzene ring) showed no effect on cMYC or MYCN levels, although its enzymatic Aurora A inhibition activity was maintained.

Based on the above results and the availability of different halogenated benzoic acid derivatives, we investigated the contribution of such substituents to cMYC/MYCN levels. Compound **13**, bearing a 4-chloro-2-fluorophenyl pyrrolidinyl methanone group, exhibited a slight reduction in enzymatic activity (Aurora A IC_50_ = 38.6 ± 7.0 nM) compared to compound **1** (Aurora A IC_50_ = 24.1 ± 7.9 nM) but was associated with the highest degree of cMYC and MYCN level reduction among compounds **1–21**. Compared with compound **13**, compound **7** lacking a fluorine atom showed a slight reduction in enzyme activity to Aurora A but lost the capability to reduce cMYC/MYCN levels. The 4-chloro-3-fluorophenyl compound **10** and 4-chloro-2,3-difluorophenyl compound **17** were less potent enzymatic inhibitors (Aurora A IC_50_ = 52.2 ± 8.1 and 64.9 ± 13.7 nM) than **13** and were moderately capable of decreasing cMYC/MYCN levels. On the other hand, introduction of fluoride at the ortho or meta positions of the phenyl ring (*i.e.*, compounds **15**, **16**, and **18**) was associated with a smaller decrease in cMYC/MYCN levels. The position of chlorine on the benzene ring was found to influence cMYC/MYCN levels; relocation of the chlorine atom of compound **13** from the para to the meta position (to give compounds **1** and **12**) diminished the reduction in cMYC/MYCN levels compared to compound **13**, as did replacement of the fluorine of **13** with chlorine (to give compound **8**) or trifluoromethyl (to give **14**) or its isostere methyl group (to give **9** and **11**). In addition, replacement of the chlorine atom on the benzene ring of compound **16** with a fluorine atom (*i.e.*, to give compound **19**) decreased Aurora A inhibition activity (IC_50_ > 100 nM). These results indicate that the positions of the halogen substituents on the benzene are crucial for Aurora A kinase activity and cMYC/MYCN level reduction. To improve the physicochemical properties of compound **13**, extensive structure–activity relationship studies were carried out by replacing its benzene moiety with a pyridine to give **20** and replacing the amide moiety of **13** with a sulfonamide to give the amide isostere **21**. However, the Aurora A inhibitory potency of these two compounds was reduced by more than 2.5-fold (IC_50_ > 100 nM).

**Figure 2:**
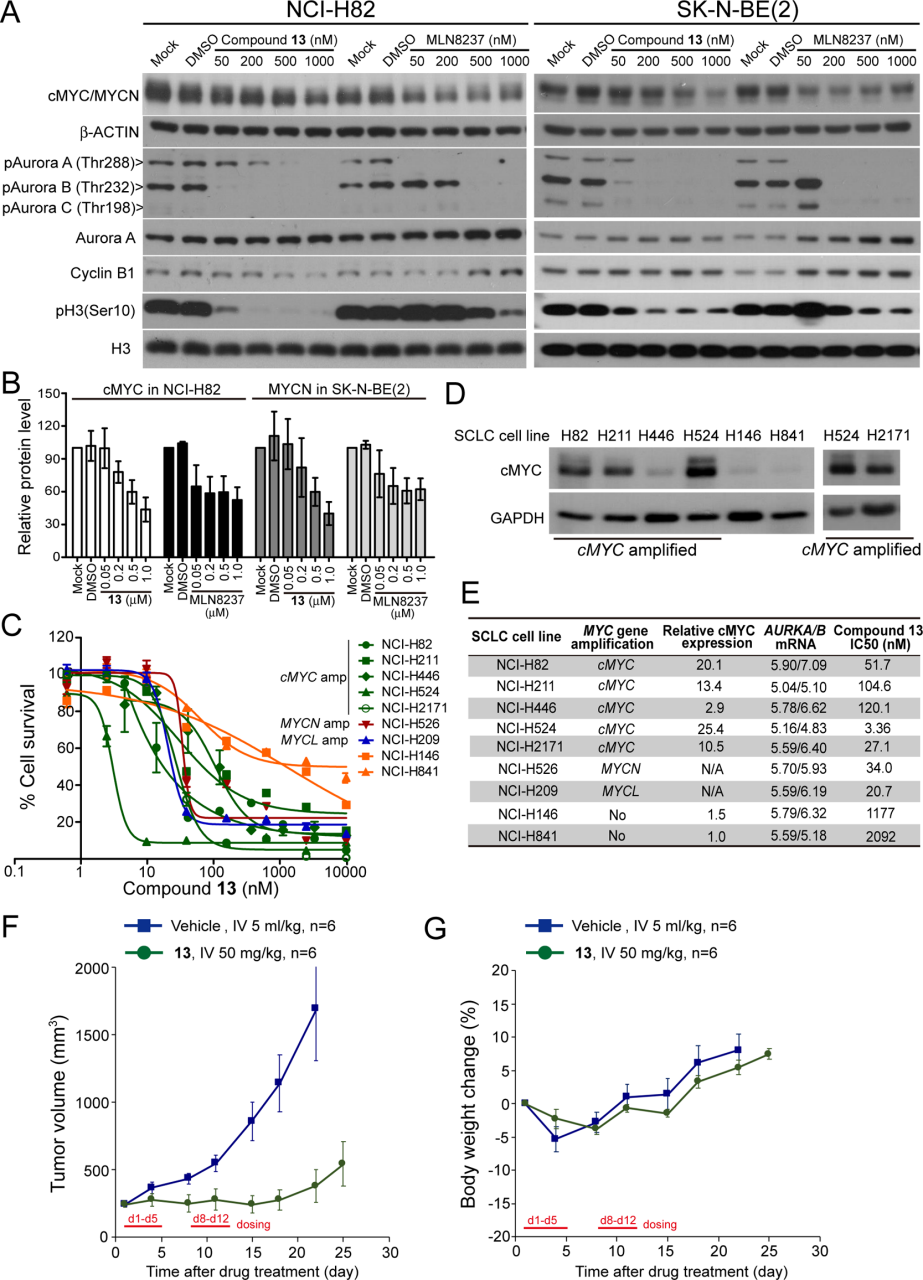
(A) Western blot analysis for the expression levels of cMYC and MYCN, phosphorylation of Aurora kinase A/B/C and cell cycle markers Cyclin B1 and pH3(Ser10) in NCI-H82 and SK-N-BE(2) cells, respectively, treated with **13** and MLN8237 for 24 h. Immunoblotting of β-ACTIN was used as loading controls. (B) Quantification of the relative protein level of cMYC and MYCN in cells treated as shown in (A). The values were obtained from triplicate experiments. (C) Percent survival of the indicated SCLC cell lines treated with **13** for 72 h. Amp, amplification. (D) Western blotting analysis for the cMYC protein expression in NCI-H82, NCI-H211, NCI-H446, NCI-H524, NCI-H2171, NCI-H146, and NCI-H841. Immunoblotting of GAPDH is shown as a loading control. Relative cMYC protein level is quantified in (E). (E) Summary of half-proliferation inhibition concentration (IC_50_) of **13** and cMYC protein levels and *AURKA* and *AURKB* mRNA levels (retrieved from the Cancer Cell Line Encyclopedia database) in SCLC cell lines shown in (C,D) and their genomic features. (F) Tumor growth curve of the xenografted NCI-H446 cells in nude mice. When the tumor size reached 200 mm^3^, mice were intravenously (IV) injected with vehicle (5 mL/kg) or **13** (50 mg/kg) using a 5-on-2-off cycle for two consecutive weeks. *P* < 0.05 from day 8 compares the **13**-treated group and vehicle. (G) Body weight of the mice treated as described in (F) was monitored twice a week for 24 days.

Single treatment of **13** in NCI-H82 and SK-N-BE(2) showed dose-dependent loss of cMYC and MYCN proteins. The phase II investigational drug MLN8237, although showed better activity than **13** in reducing cMYC/MYCN levels at lower concentrations (*i.e.*, 50 and 200 nM), was not able to induce further cMYC/MYCN level reduction at higher concentrations (*i.e.*, 500 and 1000 nM, Figure 2A,B and Supporting Information Figure S1C). In addition, whereas compound **13** is less promising in reducing the MYCN level compared to CD532 at 1.0 μM, it showed a similar trend in reducing the cMYC level in NCI-H82 cells (Supporting Information Figure S1D). The differences in the dose-dependent efficacy of **13**, CD532, and MLN8237 on cMYC/MYCN destabilization could be derived from the various conformation-specific interacting modes between Aurora A, the inhibitors, and cMYC/MYCN, which would require biochemical assays, as reported by Gilburt *et al.* for further verification.^28^

### Proliferation Inhibition of Cancer Cells

SCLC is an aggressive malignancy that accounts for around 10–15% of all lung cancers, for which no targeted therapy is currently available.^29,30^ SCLC cells are characterized by rapid proliferation, universal *RB1* and *TP53* inactivation, and high rates of MYC-family amplification.^31^ A recent study of *MYC* amplification in 77 formalin-fixed paraffin-embedded SCLC tumor samples using chromogenic *in situ* hybridization identified amplification of the *MYC*-family oncogenes in 20% of the tumors.^32,33^ Thus, we evaluated the growth-inhibitory effects of **13** on nine different SCLC cell lines: five with *cMYC* amplification, one with *MYCN* amplification, one with *MYCL* amplification, and two with no *MYC*-family amplification. As shown in Figure 2C, the sensitivity of these SCLC cells to compound **13** is significantly correlated (*R*^2^ = 0.7473, Supporting Information Figure S2A) with the protein level of cMYC and is not correlated with the mRNA levels of *AURKA* or *AURKB* (Figure 2D,E).^34^ NCI-H524 (which expressed with the highest level of cMYC) was the most sensitive to **13**, with a growth inhibition IC_50_ = 3.36 nM. The other four *cMYC*-amplified SCLC cells also demonstrated IC_50_ < 200 nM, whereas the *MYC*-unamplified SCLC cell lines (*i.e.*, NCI-H146 and NCI-H841) were less sensitive to **13** with IC_50_ > 1000 nM (Figure 2E). NCI-H526 and NCI-H209 which acquired *MYCN* and *MYCL* amplification, respectively, also showed high sensitivity to **13** (IC_50_ < 100 nM).

The antiproliferative effect of Aurora kinase inhibitors on SCLCs with *cMYC*-amplification has been reported.^35,36^ Sos *et al.* suggested that the viability of *cMYC*-amplified SCLC cells depends on cMYC and Aurora B protein expression, though knocking down Aurora B by RNA silencing did not alter the protein level of cMYC.^36^ The western blot result (Figure 2A) indicated that **13** and MLN8237 inhibited phosphorylation of both Aurora A and Aurora B in a dose-dependent manner, and compound **13** had a preferential in inhibiting Aurora B [evidenced with reduced pAurora B (Thr232) along with reduced pH3 (Ser10) and Cyclin B1] over Aurora A [evidenced with reduced pAurora A (Thr288)]. We noted that the trend of cMYC/MYCN reduction coincides better with the level of pAurora A (Thr288) than pAurora B (Thr232) in cells treated with various concentrations of **13** (Figure 2A). To elucidate the antiproliferation activity of **13** is attributed to cMYC/MYCN reduction or cell-cycle arrest induced by Aurora A and/or Aurora B kinase inhibition, we compared percent cell survival of NCI-H82 and SK-N-BE(2) with short-term (*i.e.*, 24 h) treatment of an Aurora A-selective inhibitor (*i.e.*, Aurora A inhibitor I, reported cell-free IC_50_ = 3.4 nM), an Aurora B-selective inhibitor (*i.e.*, AZD1152, reported cell-free IC_50_ = 0.37 nM), **13**, or MLN8237. Treatment of NCI-H82 with Aurora A inhibitor I or AZD1152 had a limited effect on cMYC levels (Supporting Information Figure S2B). Compound **13** and MLN8237 had better dose-dependent efficacy than Aurora A inhibitor I or AZD1152 in reducing cell viability. AZD1152 showed similar potency to **13** at lower concentrations (*i.e.*, between 1 and 15.6 nM) but failed to reduce cell viability further at higher concentrations (Supporting Information Figure S2C). The **13**-induced reduction in cMYC level is not due to reduced gene transcription (Supporting Information Figure S2D), and the cMYC protein level could be recovered by treatment with a proteasomal inhibitor MG132 (Supporting Information Figure S2E), suggesting that **13** had a minimal effect on the transcription or translation of cMYC. In addition, whereas **13** induced cell cycle arrest similar to AZD1152 (Supporting Information Figure S2F) through the inhibition on Aurora B, AZD1152 treatment had little effect on the cMYC level in NCI-H82 (Supporting Information Figure S2B). This result indicated that the reduced cMYC level was unlikely to be due to Aurora B inhibition-induced cell cycle perturbation, a conclusion similar to that of Sos *et al.*(36) On the other hand, while the hypothesis that MYC levels are impacted through a destabilization/degradation mechanism is intriguing, it is at present unclear whether the reduced cellular levels of cMYC/MYCN induced by **13** are caused by cell-cycle effects resulting from direct inhibition of Aurora kinases or Aurora A-dependent protein destabilization due to the intrinsic dependence of cMYC/MYCN levels on the cell cycle.

### Xenograft Tumor Growth Inhibition in Mice

PK studies (Table 2) of **13** undertaken in mice revealed a moderate plasma half-life (4.0 ± 0.2 h), providing an area under the curve (AUC) of 703 ± 9 ng/mL·h when delivered at 2 mg/kg intravenous (IV). Although the bioavailability of **13** is suboptimal, its cellular activity suggested potential efficacy *in vivo*. A *cMYC*-amplified SCLC cell line NCI-H446 was selected for *in vivo* efficacy study due to its stem cell-like properties and consistently high growth rate when transplanted in mice.^37,38^ Intravenous administration of **13** at 50 mg/kg led to >90% tumor growth inhibition on a 5-on-2-off dosing schedule (Figure 2F), and less than 5% body weight change was observed during the treatment (Figure 2G).

**Table 2:**
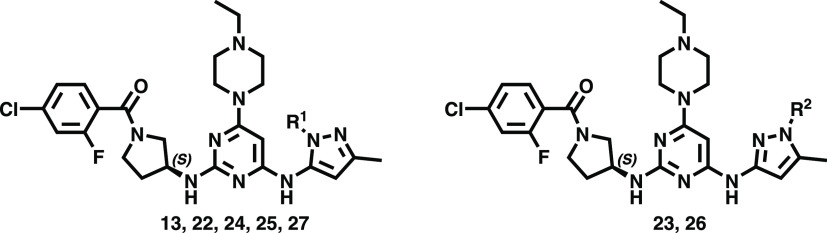

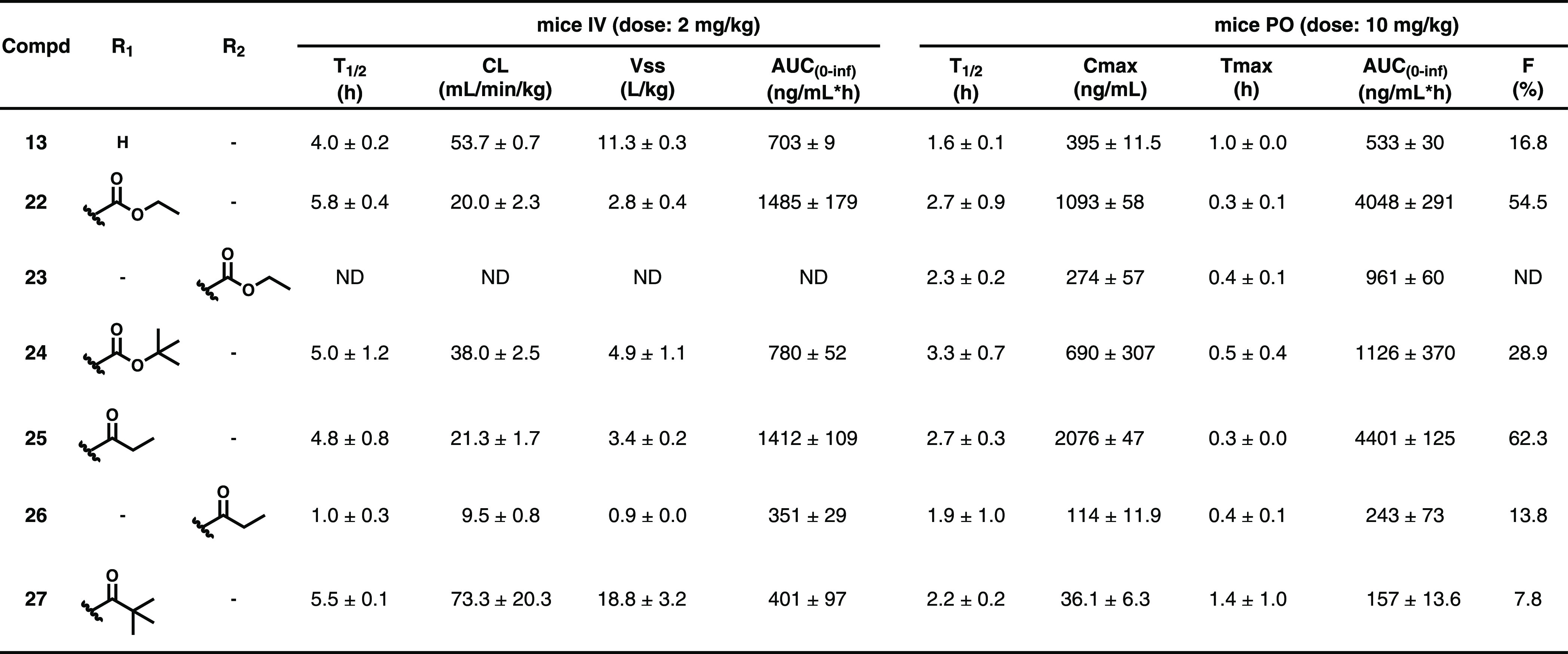
Lead Optimization of Compound **13** Using Prodrug Approaches^a^

a Values indicate the mean and standard deviation (SD) of three mice (*n* = 3). ND, not detected.

### PK Improvements to the Lead Using Prodrug Approaches

The PK study in mice established **13** to have a high clearance rate (53.7 ± 0.7 mL/min/kg) in blood. Because **13** exhibited high *in vivo* SCLC tumor inhibition efficacy with intravenous administration (Figure 2F), we attempted to improve its oral bioavailability using prodrug approaches, which balance membrane permeation, P-glycoprotein efflux, hydrolysis in the gastrointestinal lumen and intestinal cells, and nonesterase metabolism in the liver.^39^ Several classes of nitrogen-containing prodrugs have been reported, wherein polar functional groups such as carbamates, carboxamides, and sulfamides^40−42^ are used to increase permeability. Here, two types of prodrug derivatives were designed: N-carboxylate derivatives (**22–24**) and N-acyl derivatives (**25–27**). The PK properties of **13** were greatly improved by its derivatization as N-carboxylate derivative **22** and N-acyl derivative **25**; the released **13** showed AUC = 4048 ± 291 and 4401 ± 125 ng/mL·h with oral bioavailability = 54.5 and 62.3%, respectively (Table 2). On the other hand, the regioisomers **23** and **26** showed much lower AUC and had poor oral bioavailability. The reduced steric hindrance of the prodrug derivatives **23** and **26** may result in higher *in vivo* metabolic rate compared to **22** and **25**. In addition, prodrugs bearing larger substituents such as **24** (N-carboxylate derivative) and **27** (N-acyl derivative) were also designed; however, the AUC revealed no further enhancement (Table 2). The bulky substituents may increase lipophilicity, thereby reducing tissue absorption.

**Figure 3:**
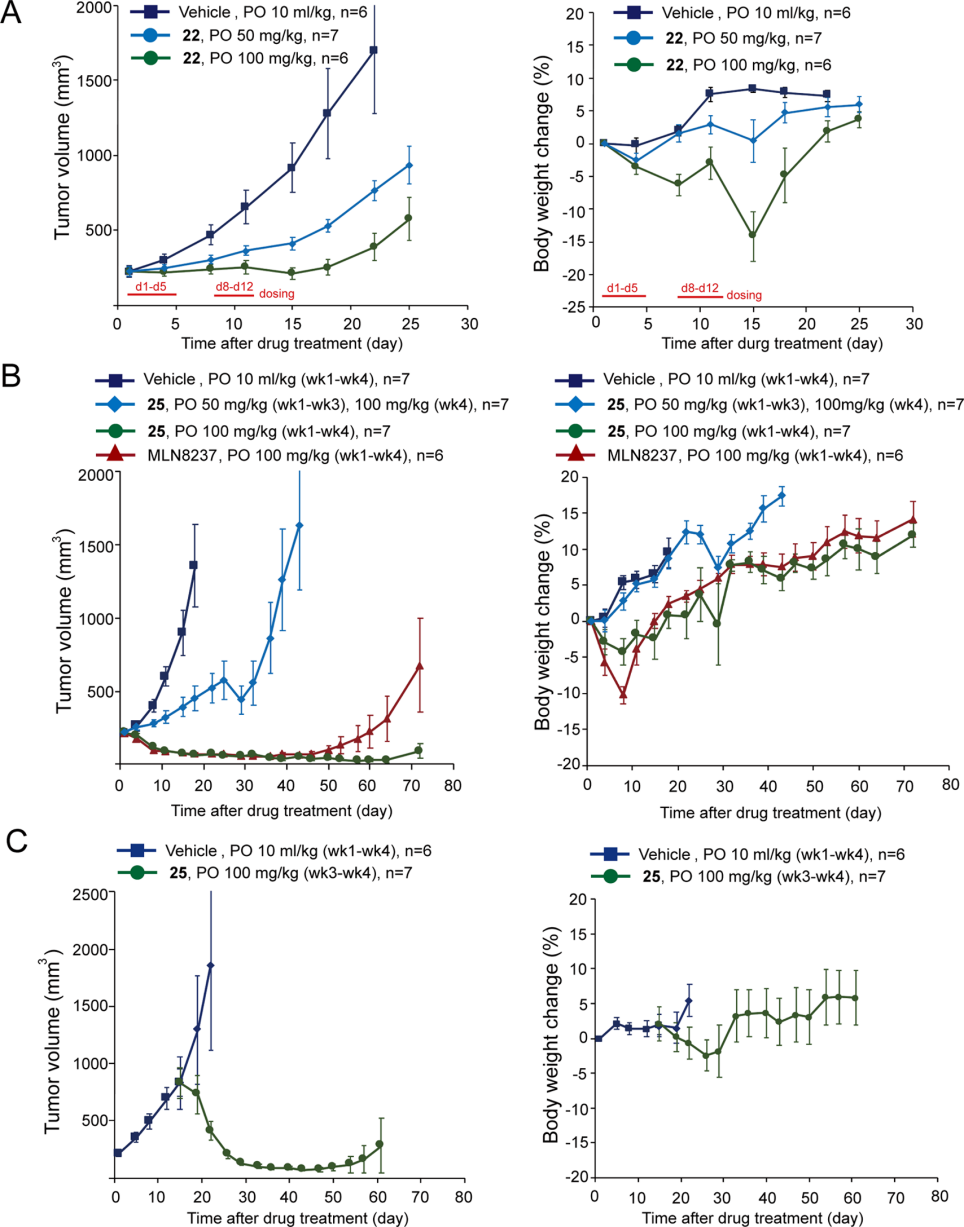
(A,B) Growth curve of xenografted NCI-H446 in mice orally (PO) administered with vehicle and the indicated dosages of **22**, **25**, and MLN8237 using a 5-on-2-off cycle, respectively. Body weight of the mice during the course is shown at right. *P* < 0.05 for tumor volume from day 4 or day 8 compares the drug-treated groups (**22**, **25**, and MLN8237) and the vehicle. (C) Mice bearing with >750 mm^3^ NCI-H446 xenograft tumors were orally treated with vehicle or **25** at 100 mg/kg from week 3 using a 5-on-2-off cycle for 2 weeks. Wk, week.

We then examined the *in vivo* tumor growth inhibition efficacy of the N-carboxylate derivative **22** and N-acyl derivative **25**, both of which showed superior oral bioavailability to **13** (Table 2). In the NCI-H446 xenograft tumor model, oral administration of **22** and **25** in mice on a 5-on-2-off regimen demonstrated dose-dependent anticancer activity (Figure 3A,B). Increasing the dose of **25** from 50 to 100 mg/kg in the fourth week further reduced the tumor volume (Figure 3B). In addition, oral administration of **25** at 100 mg/kg for four consecutive weeks induced >80% volume regression of the tumors, an observation similar to the reference compound MLN8237. Furthermore, the xenografted tumors treated with **25** remained stable for 44 days after the last dosage, whereas the MLN8237-treated group started to grow at day 22 after the last dosage. Body weight change in mice treated with **25** was less than 5% during the 4-week dosing period, suggesting its safety in mice at the 100 mg/kg dosage (Figure 3B). We noted that the body weight loss in mice administered with **25** was less severe compared to animals administered with **22** at the same dosage, although they both potentially induce neutropenia *via* the inhibition on the B-type Aurora kinase.^43^ Because **25** and **22** are both prodrugs of **13**, this result suggested that the prodrug design may alter the PK properties of the compound and thus, the overall therapeutic index. In addition to shrinking of the NCI-H446 xenograft tumors with volume ≅200 mm^3^, **25** also showed great potency to shrink tumors with volume >750 mm^3^ (Figure 3C). These results demonstrate the *in vivo* activity of **22** and **25** on *cMYC*-amplified SCLC.

### *In Vivo* MYC Protein Level and Cancer Cell Apoptosis

**Figure 4:**
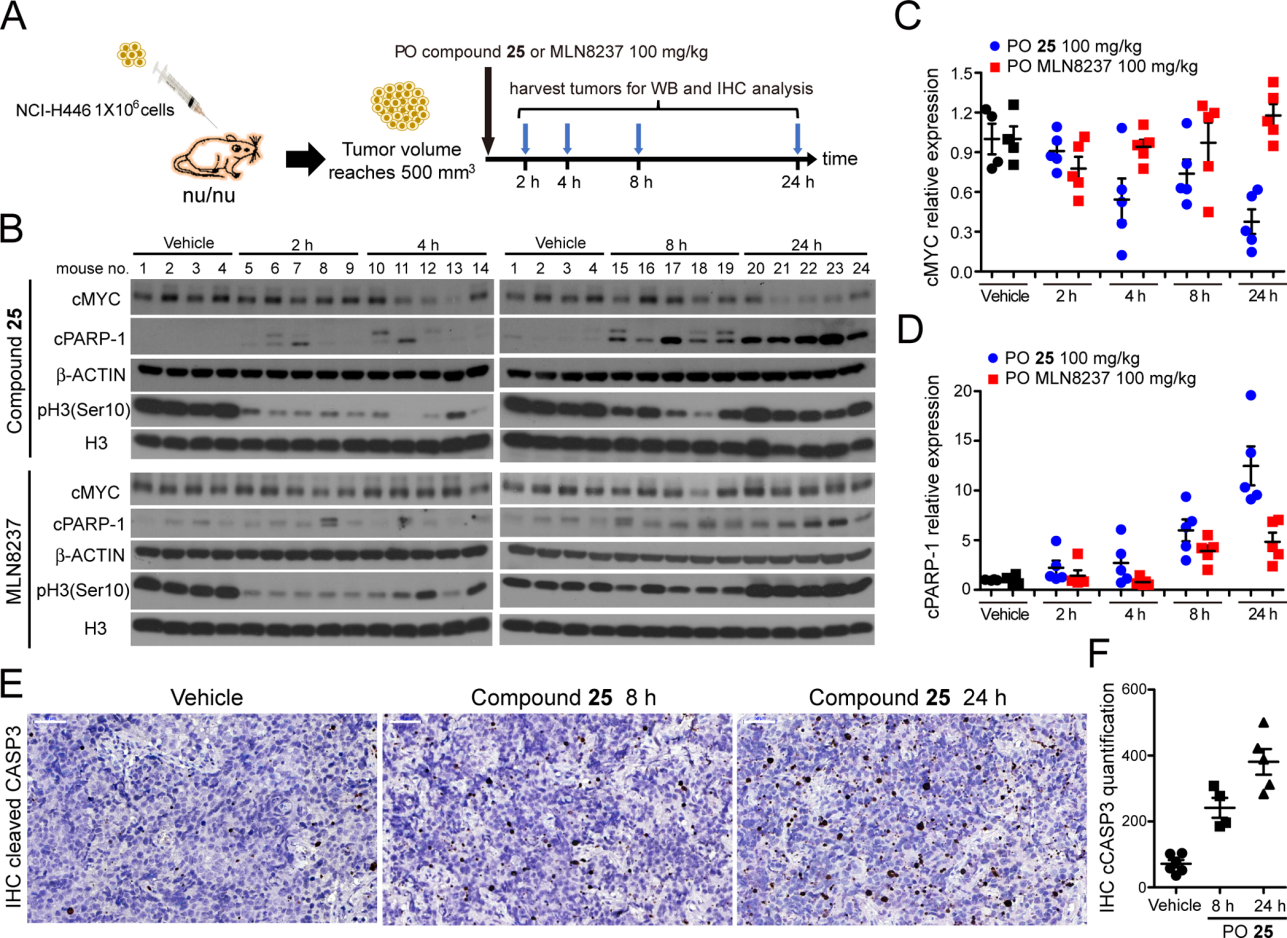
(A) Schematic illustration of the NCI-H446 tumor xenograft and drug treatment protocol in nude mice. (B) Western blot analysis for the expression of cMYC, cleaved PARP-1 (cPARP-1), a mitotic marker pH3(Ser10), and a histone H3 loading control in xenografted NCI-H446 tumors in mice oral (PO) administered with the vehicle (10 mL/kg), **25** at 100 mg/kg, or MLN8237 at 100 mg/kg. Tumors were harvested after 2, 4, 8, and 24 h of drug treatment, respectively. Relative protein levels of cMYC and cleaved cPARP-1 in tumors were quantified in (C,D). (E) Immunohistochemical analysis for the expression of cleaved caspase-3 (*i.e.*, CASP3, brown color) to mark apoptotic cells in NCI-H446 xenograft tumors harvested from mice with vehicle treatment or orally administered with **25**. The images were recorded at 400× magnification. (F) Quantification of the number of cleaved CASP3-positive cells in the IHC profile shown in (E). Cell numbers in tissues from four to six mice were quantified for each set of experimental conditions.

We next evaluated the influence of **25** and MLN8237 on the cMYC level *in vivo*. Mice bearing xenograft NCI-H446 tumors >500 mm^3^ in volume were orally administered a single dose of **25** or MLN8237 at 100 mg/kg. Tumors were harvested after 2, 4, 8, and 24 h of the treatment, respectively (Figure 4A). Whole tumor tissues were collected for western blotting and immunohistochemistry (IHC) analyses. The phosphorylation level of histone H3 at Ser10 [*i.e.*, pH3(Ser10)], a substrate of Aurora B kinase, was reduced by >90% in the tumors treated with either **25** or MLN8237 at 2 h after drug administration, suggesting the effectiveness of the compounds on Aurora kinase inhibition and cell-cycle arrest at this time point. The western blot result of phosphorylated Aurora A/B/C was not provided due to the inability of detecting the signal in the tumor tissues. The protein level of cMYC gradually decreased in the tumors treated with **25** and was around 37% (*p* = 0.0037, *t*-test) of that in the vehicle-treated tumors (1.0 ± 0.230 in vehicle *vs* 0.374 ± 0.204 in tumors at 24 h post-treatment, Figure 4B,C) 24 h after the dosing of **25**. On the other hand, treatment of MLN8237 had marginal effect on the cMYC level, an observation similar to the report of Mollaoglu *et al.*(29) This observation is perhaps due to the less significant dose-dependent efficacy of MLN8237 on cMYC level reduction (Figure 2A) or the overall PK properties of the compound.

Apoptosis was assessed using levels of PARP-1, one of several known cellular substrates of caspases; cleavage of PARP-1 by caspases is considered a hallmark of apoptosis.^44^ Cleaved PARP-1 (cPARP-1) levels were significantly upregulated in NCI-H446 tumor tissues isolated from mice administered with **25** or MLN8237 after 24 h (*p* = 0.0013 for 25 and *p* = 0.0083 for MLN8237, *t*-test), although the level of increment was less significant in MLN8237- than in **25**-treated tumors (Figure 4B,D). The IHC results showed that the incidence of cleaved Caspase-3 (CASP3) was also greatly increased (Figure 4E,F). These data suggested that **25** induced cMYC level reduction, resulting in CASP3-associated programmed cell death, and tumor regression.

## Conclusions

Novel small molecules that promote degradation of MYC-family oncoproteins would be a great advancement in cancer treatment. By the rational design of an Aurora A kinase inhibitor able to induce the DFG-out conformation using computer modeling, we successfully identified an initial hit compound **1**, which marginally reduced levels of cMYC and MYCN. Screening of different halogen substituents on the phenyl group resulted in compound **13** (*S*)-(4-chloro-2-fluorophenyl) (3-((4-(4-ethylpiperazin-1-yl)-6-((5-methyl-1*H*-pyrazol-3-yl)amino)pyrimidin-2-yl)amino)pyrrolidin-1-yl)methanone), which reduced cMYC and MYCN levels by >50% at 1.0 μM. The reduced cMYC/MYCN levels could be attributed to the inhibitor’s effects on cell cycle progression and/or protein stability. Compound **13** showed significantly enhanced inhibition potency against *MYC*-amplified SCLCs than *MYC*-unamplified SCLCs and tumor growth inhibition activity in a *cMYC*-amplified SCLC cell line NCI-H446 xenograft mouse model when administered intravenously. Lead optimization focusing on oral bioavailability led to the discovery of compound **25**, a N-acyl prodrug of **13**, which demonstrated improved oral availability and excellent NCI-H446 SCLC xenografted tumor regression in mice. These results support the potential of **25** for further development as an anticancer drug targeting *MYC*-amplified malignancies such as SCLC.

## Experimental Section

### General Chemistry

Unless otherwise stated, all reagents used were commercially available with suppliers and used as supplied. Reactions requiring anhydrous conditions were performed in flame-dried glassware and cooled under an argon or nitrogen atmosphere. All reactions were carried out under argon or nitrogen and monitored by analytical thin-layer chromatography using glass-backed plates (5 × 10 cm) precoated with silica gel 60 F_254_ as supplied by Merck; zones were detected visually under UV irradiation (254 nm) or by spraying with phosphomolybdic acid reagent (Sigma-Aldrich, USA), followed by heating to 80 °C. Flash column chromatography using silica gel 60 of 230–400 mesh size (Merck) was used routinely for purification and separation of product mixtures. ^1^H and ^13^C NMR spectra were recorded on Varian Mercury-300, Varian Mercury-400, Bruker NMR DMX-600, or Varian VNMRS-700 spectrometers. Chloroform-*d* or dimethyl sulfoxide-*d*_6_ or methanol-*d*_4_ was used as the solvent and tetramethylsilane (TMS) (δ 0.00 ppm) as an internal standard. Chemical shift values are reported in parts per million relative to the TMS in delta (δ) units. Multiplicities are recorded as s (singlet), br s (broad singlet), d (doublet), t (triplet), q (quartet), quint (quintet), dd (doublet of doublets), dt (doublet of triplets), ddd (doublet of doublets of doublets), and m (multiplet). Coupling constants (*J*) are expressed in hertz. Electrospray mass spectroscopy (ESMS) spectra were recorded as *m*/*z* values using an Agilent 1100 MSD mass spectrometer. All test compounds displayed more than 95% purity, as determined by a Hitachi 2000 series HPLC system using a C-18 column (Agilent Eclipse XDB-C18 5 μm, 4.6 mm × 150 mm, USA). Mobile phase A: acetonitrile; mobile phase B: 2 mM ammonium acetate aqueous solution containing 0.1% formic acid. The gradient system started from A/B (10%:90%) to A/B (90%:10%) with a flow rate of 0.5 mL/min, and the injection volume was 20 μL. The system was operated at 25 °C. Peaks were detected at 254 nm. The IUPAC nomenclature of compounds was determined with ACD/Name Pro software. Detailed characterization data for each compound are reported in the Supporting Information.

#### 2,6-Dichloro-*N*-(5-methyl-1*H*-pyrazol-3-yl)pyrimidin-4-amine (**29**)

3-Amino-5-methylpyrazole (7.96 g, 81.9 mmol) was added to a solution of 2,4,6-trichloropyrimidine **28** (10 g, 54.6 mmol), triethylamine (11.4 mL, 81.9 mmol) in THF (200 mL). The reaction mixture was heated at 50 °C, stirred for 16 h, and then quenched with brine (100 mL). The aqueous phase was extracted with ethyl acetate (3 × 200 mL). The combined organic extracts were washed with water and brine, dried over magnesium sulfate, and filtered. The filtrate was concentrated to get crude residue. The residue was purified by flash column chromatography over silica gel with *n*-hexane/ethyl acetate (1:1) to afford compound **29** (10 g, 41.2 mmol, 75% yield) as a white solid. ^1^H NMR (400 MHz, CDCl_3_): δ 7.75 (s, 1H), 5.99 (s, 1H), 2.35 (s, 3H); LCMS (ESI) *m*/*z*: 244 [M + H]^+^.

#### (*S*)-(3-Chloro-2-fluorophenyl) (3-((4-chloro-6-((5-methyl-1*H*-pyrazol-3-yl)amino)pyrimidin-2-yl)amino)pyrrolidin-1-yl)methanone (**30**)

A solution of compound **29** (5 g, 20.5 mmol), (*S*)-(3-aminopyrrolidin-1-yl) (3-chloro-2-fluorophenyl)methanone (6.5 g, 26.7 mmol), triethylamine (4.3 mL, 30.8 mmol) in 1-pentanol (5 mL) was heated at 120 °C for 6 h and then poured into water (300 mL). A precipitate formed, which was collected and purified by flash column chromatography over silica gel with dichloromethane/methanol (94:6) to give compound **30** (4.7 g, 10.5 mmol, 51% yield) as a yellow solid. ^1^H NMR (300 MHz, CDCl_3_): δ 10.65 (br s, 1H), 7.45 and 7.45 (t, *J* = 7.2 Hz, 1H), 7.22–7.08 (m, 1H), 6.35 and 6.31 (s, 1H), 5.98 and 5.92 (s, 1H), 4.85 and 4.64 (br s, 1H), 4.28–4.10 (m, 1H), 3.87–3.65 (m, 2H), 3.48–3.25 (m, 2H), 2.34 and 2.33 (s, 3H), 2.28–2.43 (m, 2H); LCMS (ESI) *m*/*z*: 450.1 [M + H]^+^.

### General Procedure for the Synthesis of Compounds **1–6**

The general procedure is illustrated below with compound **1** as a specific example.

#### (*S*)-(3-Chloro-2-fluorophenyl) (3-((4-(4-ethylpiperazin-1-yl)-6-((5-methyl-1*H*-pyrazol-3-yl)amino)pyrimidin-2-yl)amino)pyrrolidin-1-yl)methanone (**1**)

A solution of compound **30** (200 mg, 0.45 mmol) and 1-ethylpiperazine (103 mg, 0.9 mmol) in 1-pentanol (1 mL) was heated at 140 °C for 2 h and then diluted with brine (100 mL). The aqueous phase was extracted with ethyl acetate (3 × 200 mL). The combined organic extracts were washed with water and brine, dried over magnesium sulfate, and filtered. The filtrate was concentrated to give the crude residue, which was purified by flash column chromatography over silica gel with dichloromethane/methanol (90:10) to afford compound **1** (137 mg, 0.26 mmol, 58% yield) as a yellow solid. ^1^H NMR (400 MHz, CDCl_3_): δ 7.43 and 7.43 (t, *J* = 7.5 Hz, 1H), 7.21 and 7.20 (q, *J* = 7.5 Hz, 1H), 7.09 (t, *J* = 7.5 Hz, 1H), 5.93 and 5.87 (s, 1H), 5.62 and 5.59 (s, 1H), 4.72 and 4.58 (br s, 1H), 4.13 (m, 1H), 3.82–3.66 (m, 2H), 3.62–3.42 (m, 5H), 3.41–3.25 (m, 1H), 2.51–2.40 (m, 6H), 2.31 and 2.29 (s, 3H), 2.28–2.21 (m, 1H), 2.16–2.05 (m, 1H), 1.12 and 1.12 (t, *J* = 7.2 Hz, 3H); ^13^C NMR (100 MHz, DMSO-*d*_6_): δ 163.51, 163.26, 162.82, 161.00 × 2, 160.81, 153.13 (d, *J*_*C–F*_ = 244.0 Hz), 131.60, 127.52, 127.02 and 126.92 (d, *J*_*C–F*_ = 17.9 Hz), 125.97, 120.25 and 120.18 (d, *J*_*C–F*_ = 17.9 Hz), 75.55, 53.39 and 51.62, 52.14, 52.02, 51.73, 50.80 and 49.70, 46.11 and 44.06, 43.72, 31.21 and 29.54, 11.92 × 2; LCMS (ESI) *m*/*z*: 528.3 [M + H]^+^; HRMS (ESI) calcd for C_25_H_31_ClFN_9_O [M + H]^+^*m*/*z*: 528.2402; found, 528.2402; HPLC purity = 100%, *t*R = 10.60 min.

#### (*S*)-(3-Chloro-2-fluorophenyl) (3-((4-((5-methyl-1*H*-pyrazol-3-yl)amino)-6-(4-methylpiperazin-1-yl)pyrimidin-2-yl)amino)pyrrolidin-1-yl)methanone (**2**)

Similar to the reaction procedures for **1**, a solution of compound **30** (200 mg, 0.45 mmol) and 1-methylpiperazine (90 mg, 0.9 mmol) in 1-pentanol (0.9 mL) was heated at 140 °C for 2 h. After a usual workup, the crude residue was purified by silica gel column chromatography with dichloromethane/methanol (90:10) to afford compound **2** (131 mg, 0.26 mmol, 57% yield) as a yellow solid. ^1^H NMR (300 MHz, CDCl_3_): δ 7.43 (t, *J* = 6.6 Hz, 1H), 7.24–7.18 (m, 1H), 7.09 (t, *J* = 7.8 Hz, 1H), 5.93 and 5.87 (s, 1H), 5.63 and 5.60 (s, 1H), 4.72 and 4.78 (br s, 1H), 4.17–4.07 (m, 1H), 3.82–3.24 (m, 8H), 2.46–2.37 (m, 4H), 2.35–2.28 (m, 6H), 2.57–2.04 (m, 2H); ^13^C NMR (100 MHz, DMSO-*d*_6_): δ 163.49, 163.23, 162.80, 160.97 × 2, 160.81, 153.09 (d, *J*_*C–F*_ = 247.0 Hz), 131.59 (d, *J*_*C–F*_ = 5.3 Hz), 127.50, 127.00 and 126.91 (d, *J*_*C–F*_ = 18.0 Hz), 125.98, 120.24 and 120.17 (d, *J*_*C–F*_ = 17.5 Hz), 75.57, 54.35, 54.20, 53.34 and 51.59, 50.76 and 49.67, 46.09 and 44.05, 45.82, 43.62, 31.20 and 29.52, 11.47; LCMS (ESI) *m*/*z*: 514.2 [M + H]^+^; HRMS (ESI) calcd for C_24_H_29_ClFN_9_O [M + H]^+^*m*/*z*: 514.2246; found, 514.2249; HPLC purity = 100%, *t*R = 11.01 min.

#### (*S*)-(3-Chloro-2-fluorophenyl) (3-((4-(3-(dimethylamino)azetidin-1-yl)-6-((5-methyl-1*H*-pyrazol-3-yl)amino)pyrimidin-2-yl)amino)pyrrolidin-1-yl)methanone (**3**)

Similar to the reaction procedures for **1**, a solution of compound **30** (200 mg, 0.45 mmol) and *N*,*N*-dimethylazetidin-3-amine (90 mg, 0.9 mmol) in 1-pentanol (0.9 mL) was heated at 140 °C for 2 h. After a usual workup, the crude residue was purified by silica gel column chromatography with dichloromethane/methanol (90:10) to afford compound **3** (60 mg, 0.12 mmol, 26% yield) as a yellow solid. ^1^H NMR (300 MHz, CDCl_3_): δ 7.42 and 7.42 (t, *J* = 7.2 Hz, 1H), 7.24–7.17 (m, 1H), 7.09 (t, *J* = 7.8 Hz, 1H), 5.91 and 5.85 (s, 1H), 5.29 and 5.26 (s, 1H), 4.71 and 4.58 (br s, 1H), 4.09–3.90 (m, 3H), 3.81–3.65 (m, 4H), 3.51–3.10 (m, 3H), 2.29 and 2.28 (s, 3H), 2.23–2.03 (m, 8H); ^13^C NMR (100 MHz, DMSO-*d*_6_): δ 165.09, 164.93, 162.76, 161.16, 161.11, 159.83, 153.09 (d, *J*_*C–F*_ = 246.3 Hz), 131.58 (d, *J*_*C–F*_ = 3.8 Hz), 127.52 (d, *J*_*C–F*_ = 3.8 Hz), 126.99 and 126.91 (d, *J*_*C–F*_ = 18.3 Hz), 125.98, 120.19 and 120.14 (d, *J*_*C–F*_ = 17.6 Hz), 74.46, 55.94, 55.90, 53.69, 53.56, 53.36 and 51.56, 50.71 and 49.58, 46.07 and 44.03, 41.49, 41.45, 31.13 and 29.56; LCMS (ESI) *m*/*z*: 514.2 [M + H]^+^; HRMS (ESI) calcd for C_24_H_29_ClFN_9_O [M + H]^+^*m*/*z*: 514.2246; found, 514.2238; HPLC purity = 100%, *t*R = 10.95 min.

#### (3-Chloro-2-fluorophenyl) ((*S*)-3-((4-((*S*)-3-(dimethylamino)pyrrolidin-1-yl)-6-((5-methyl-1*H*-pyrazol-3-yl)amino)pyrimidin-2-yl)amino)pyrrolidin-1-yl)methanone (**4**)

Similar to the reaction procedures for **1**, a solution of compound **30** (200 mg, 0.45 mmol) and (*S*)–*N*,*N*-dimethylpyrrolidin-3-amine (102 mg, 0.9 mmol) in 1-pentanol (0.9 mL) was heated at 140 °C for 2 h. After workup, the crude residue was purified by silica gel column chromatography with dichloromethane/methanol (90:10) to afford compound **4** (121 mg, 0.23 mmol, 51% yield) as a yellow solid. ^1^H NMR (300 MHz, CDCl_3_): δ 7.47–7.40 (m, 1H), 7.33–7.27 (m, 1H), 7.16–7.08 (m, 1H), 5.90 and 5.85 (s, 1H), 5.46–5.36 (m, 2H), 4.59 (br s, 1H), 4.10–3.60 (m, 3H), 3.60–3.10 (m, 5H), 2.88–2.75 (m, 1H), 2.35–2.24 (m, 11H), 1.96–1.80 (m, 2H); ^13^C NMR (100 MHz, DMSO-*d*_6_): δ 162.84, 161.38, 161.22, 161.02 and 160.98, 159.96, 159.93, 153.14 (d, *J*_*C–F*_ = 247.1 Hz), 131.62 (d, *J*_*C–F*_ = 5.4 Hz), 127.52 (d, *J*_*C–F*_ = 3.0 Hz), 127.02 and 126.95 (d, *J*_*C–F*_ = 17.6 Hz), 126.03, 120.25 and 120.22 (d, *J*_*C–F*_ = 17.6 Hz), 75.32, 64.65 and 64.57, 53.50 and 51.69, 50.86 and 49.75, 50.22, 49.96, 46.14 and 44.12, 45.14, 44.99, 43.81, 43.75, 31.20 and 29.25, 29.62; LCMS (ESI) *m*/*z*: 528.2 [M + H]^+^; HRMS (ESI) calcd for C_25_H_31_ClFN_9_O [M + H]^+^*m*/*z*: 528.2402; found, 528.2389; HPLC purity = 98.47%, *t*R = 11.19 min.

#### (*S*)-(3-Chloro-2-fluorophenyl) (3-((4-((2-(dimethylamino)ethyl) (methyl)amino)-6-((5-methyl-1*H*-pyrazol-3-yl)amino)pyrimidin-2-yl)amino)pyrrolidin-1-yl)methanone (**5**)

Similar to the reaction procedures for **1**, a solution of compound **30** (200 mg, 0.45 mmol) and *N*1,*N*1,*N*2-trimethylethane-1,2-diamine (92 mg, 0.9 mmol) in 1-pentanol (0.9 mL) was heated at 140 °C for 2 h. After workup, the crude residue was purified by silica gel column chromatography with dichloromethane/methanol (90:10) to afford compound **5** (102 mg, 0.2 mmol, 44% yield) as a yellow solid. ^1^H NMR (400 MHz, CDCl_3_): δ 7.43 (t, *J* = 7.6 Hz, 1H), 7.25–7.20 (m, 1H), 7.10 (t, *J* = 7.8 Hz, 1H), 5.93 and 5.88 (s, 1H), 5.51 and 5.49 (s, 1H), 4.69 and 4.60 (br s, 1H), 4.15–4.04 (m, 1H), 3.83–3.44 (m, 5H), 3.42–3.25 (m, 1H), 2.98 and 2.94 (s, 3H), 2.41–2.35 (m, 2H), 2.32–2.25 (m, 7H), 2.23 (s, 3H), 2.20–2.05 (m, 1H); ^13^C NMR (100 MHz, DMSO-*d*_6_): δ 162.81, 162.67, 160.79 × 2, 160.42 × 2, 153.09 (d, *J*_*C–F*_ = 247.0 Hz), 131.61 (d, *J*_*C–F*_ = 6.9 Hz), 127.49, 126.98 and 126.91 (d, *J*_*C–F*_ = 17.9 Hz), 126.03, 120.22 (d, *J*_*C–F*_ = 17.5 Hz), 74.63, 56.39, 53.61 and 51.63, 50.81 and 49.73, 46.53, 46.38, 46.07 and 44.15, 45.50, 45.40, 35.38, 31.20 and 29.59; LCMS (ESI) *m*/*z*: 516.2 [M + H]^+^; HRMS (ESI) calcd for C_24_H_31_ClFN_9_O [M + H]^+^*m*/*z*: 516.2402; found, 516.2402; HPLC purity = 99.91%, *t*R = 11.16 min.

#### *tert*-Butyl (*S*)-3-((4,6-dichloropyrimidin-2-yl)amino)pyrrolidine-1-carboxylate (**32**)

*tert*-Butyl (*S*)-3-aminopyrrolidine-1-carboxylate (20 g, 105.7 mmol) was added dropwise at −70 °C over 1 h to a solution of the starting material 4,6-dichloro-2-(methylsulfonyl)pyrimidine **31** (20 g, 88.1 mmol) and triethylamine (25.5 mL, 176.2 mmol) in THF (200 mL). The reaction mixture was warmed up to room temperature, stirred for 6 h, and then quenched with brine (100 mL). The aqueous phase was extracted with ethyl acetate (3 × 200 mL). The combined organic extracts were washed with water and brine, dried over magnesium sulfate, and filtered. The filtrate was concentrated to afford the crude residue. The residue was purified by flash column chromatography over silica gel with *n*-hexane/ethyl acetate (4:1) to afford compound **32** (16.4 g, 49.3 mmol, 56% yield) as a white solid. ^1^H NMR (400 MHz, CDCl_3_): δ 6.65 (s, 1H), 5.42 (br s, 1H), 4.60–4.47 (m, 1H), 3.69 (dd, *J* = 11.2, 6.0 Hz, 1H), 3.52–3.40 (m, 3H), 3.35–3.15 (m, 1H), 2.23 (m, 1H), 1.85–1.75 (m, 1H), 1.47 (s, 9H); LCMS (ESI) *m*/*z*: 355.1 [M + Na]^+^.

#### *tert*-Butyl (*S*)-3-((4-chloro-6-((5-methyl-1*H*-pyrazol-3-yl)amino)pyrimidin-2-yl)amino)pyrrolidine-1-carboxylate (**33**)

A solution of compound **32** (7 g, 21.0 mmol), 3-amino-5-methylpyrazole (8.1 g, 84.0 mmol), triethylamine (3.5 mL, 25.2 mmol), and NaI (4.7 g, 31.5 mmol) in DMSO (70 mL) was stirred at 90 °C for 16 h. The solution was cooled down to room temperature and poured into water. A precipitate formed, which was collected and purified by flash column chromatography over silica gel with *n*-hexane/ethyl acetate (1:1) to give compound **33** (7 g, 17.9 mmol, 85% yield) as a yellow solid. ^1^H NMR (400 MHz, CDCl_3_): δ 6.32 (s, 1H), 5.93 (s, 1H), 4.48 (br s, 1H), 3.85–3.60 (m, 1H), 3.60–3.40 (m, 4H), 2.31 (s, 3H), 1.46 (s, 9H); LCMS (ESI) *m*/*z*: 394.1 [M + H]^+^.

#### *tert*-Butyl (*S*)-3-((4-(4-ethylpiperazin-1-yl)-6-((5-methyl-1*H*-pyrazol-3-yl)amino)pyrimidin-2-yl)amino)pyrrolidine-1-carboxylate (**34**)

A solution of compound **33** (7 g, 17.8 mmol) and 1-ethylpiperazine (4.1 g, 35.6 mmol) in 1-pentanol (14 mL) was heated at 140 °C for 2 h and then quenched with brine (100 mL). The aqueous phase was extracted with ethyl acetate (3 × 200 mL). The combined organic extracts were washed with water and brine, dried over magnesium sulfate, and filtered. The filtrate was concentrated to give the crude residue, which was purified by flash column chromatography over silica gel with ethyl acetate/methanol (90:10) to afford compound **34** (7 g, 14.9 mmol, 84% yield) as a yellow solid. ^1^H NMR (400 MHz, CDCl_3_): δ 5.87 (s, 1H), 5.62 (s, 1H), 4.70–4.39 (m, 1H), 3.80–3.30 (m, 7H), 2.52–2.40 (m, 6H), 2.27 (s, 3H), 2.24–2.04 (m, 2H), 1.46 (s, 9H), 1.11 (t, *J* = 7.2 Hz, 3H); LCMS (ESI) *m*/*z*: 472.1 [M + H]^+^.

### General Procedure for the Synthesis of Compounds **6–21**

The general procedure is illustrated below with compound **13** as a specific example.

#### (*S*)-(4-Chloro-2-fluorophenyl) (3-((4-(4-ethylpiperazin-1-yl)-6-((5-methyl-1*H*-pyrazol-3-yl)amino)pyrimidin-2-yl)amino)pyrrolidin-1-yl)methanone (**13**)

A solution of 2 *N* hydrochloric acid in ether (1.06 mL, 2.1 mmol) was added to a solution of compound **34** (200 mg, 0.42 mmol) in dichloromethane/methanol (2:1, 1 mL) at room temperature with stirring. The resulting mixture was stirred at room temperature for 4 h and then concentrated *in vacuo* to give the crude amine salt, which was used without further purification.

Triethylamine (0.35 mL, 2.52 mmol), 4-chloro-2-fluorobenzoic acid (88 mg, 0.5 mmol), and propanephosphonic acid anhydride (T3P) ≥50 wt % in ethyl acetate (400 mg, 0.63 mmol) were added to a solution of amine salt in DMF/dichloromethane (1:3, 4 mL) at room temperature. The resulting mixture was stirred at room temperature for 16 h and then quenched with brine (20 mL). The aqueous phase was extracted with ethyl acetate (3 × 30 mL). The combined organic extracts were washed with water and brine, dried over magnesium sulfate, and filtered. The filtrate was concentrated to afford the crude residue, which was purified by flash column chromatography over silica gel with ethyl acetate/methanol (85:15) to give compound **13** (128 mg, 0.24 mmol, 58% yield) as a white solid. ^1^H NMR (400 MHz, CD_3_OD): δ 7.46–7.25 (m, 3H), 5.95–5.75 (m, 1H), 5.62–5.45 (m, 1H), 4.53 and 4.36 (br s, 1H), 3.98–3.64 (m, 3H), 3.63–3.56 (m, 2H), 3.54–3.40 (m, 4H), 2.60–2.40 (m, 6H), 2.36–2.29 (m, 1H), 2.23 and 2.22 (s, 3H), 2.10–1.90 (m, 1H), 1.15 and 1.14 (t, *J* = 7.2 Hz, 3H); ^13^C NMR (100 MHz, CD_3_OD): δ 166.60, 165.34, 165.17, 162.61 × 3, 159.78 (d, *J*_*C–F*_ = 249.3 Hz), 137.99 (d, *J*_*C–F*_ = 9.9 Hz), 131.19 (d, *J*_*C–F*_ = 4.6 Hz), 126.52 (d, *J*_*C–F*_ = 3.1 Hz), 125.05 (t, *J*_*C–F*_ = 16.8 Hz), 118.01 and 117.92 (d, *J*_*C–F*_ = 25.1 Hz), 77.43, 55.30 and 53.37, 53.57, 53.51, 52.49 and 51.48, 47.82 and 45.63, 44.88, 44.85, 32.73 and 31.11, 11.86, 11.82; LCMS (ESI) *m*/*z*: 528.2 [M + H]^+^; HRMS (ESI) calcd for C_25_H_31_ClFN_9_O [M + H]^+^*m*/*z*: 528.2402; found, 528.2408; HPLC purity = 99.57%, *t*R = 10.68 min.

#### (*S*)-(3-((4-(4-Ethylpiperazin-1-yl)-6-((5-methyl-1*H*-pyrazol-3-yl)amino)pyrimidin-2-yl)amino)pyrrolidin-1-yl) (phenyl)methanone (**6**)

Similar to the reaction procedures for **13**, a solution of 2 *N* hydrochloric acid in ether (1.06 mL, 2.1 mmol) was added to a solution of compound **34** (200 mg, 0.42 mmol) in dichloromethane/methanol (2:1, 1 mL), and the resulting mixture was stirred at room temperature for 4 h. After concentration *in vacuo*, the residue was dissolved in DMF/dichloromethane (1:3, 4 mL), and then, triethylamine (0.35 mL, 2.52 mmol), benzoic acid (61 mg, 0.5 mmol), and propanephosphonic acid anhydride (T3P) ≥50 wt % in ethyl acetate (400 mg, 0.63 mmol) were added. The solution was stirred at room temperature for 16 h. After workup, the crude residue was purified by silica gel column chromatography with ethyl acetate/methanol (85:15) to afford compound **6** (132 mg, 0.28 mmol, 66% yield) as a yellow solid. ^1^H NMR (300 MHz, CDCl_3_): δ 7.49–7.44 (m, 2H), 7.42–7.34 (m, 3H), 5.91 and 5.84 (s, 1H), 5.63 and 5.59 (s, 1H), 4.68 and 4.55 (br s, 1H), 4.15–4.05 (m, 1H), 3.82–3.40 (m, 8H), 2.54–2.42 (m, 6H), 2.26 and 2.29 (s, 3H), 2.25–2.03 (m, 2H), 1.14 and 1.13 (t, *J* = 7.1 Hz, 3H); ^13^C NMR (100 MHz, DMSO-*d*_6_): δ 168.49, 163.44, 163.21, 160.94 × 2, 160.74, 137.01 and 136.94, 129.77, 128.26 and 128.19, 127.10, 75.50, 54.81 and 51.66, 52.01, 51.90, 51.12 and 49.49, 47.31 and 44.34, 43.55 × 2, 31.76 and 29.40, 11.78 × 2; LCMS (ESI) *m*/*z*: 476.2 [M + H]^+^; HRMS (ESI) calcd for C_25_H_33_N_9_O [M + H]^+^*m*/*z*: 476.2887; found, 476.2883; HPLC purity = 96.41%, *t*R = 9.63 min.

#### (*S*)-(4-Chlorophenyl) (3-((4-(4-ethylpiperazin-1-yl)-6-((5-methyl-1*H*-pyrazol-3-yl)amino)pyrimidin-2-yl)amino)pyrrolidin-1-yl)methanone (**7**)

Similar to the reaction procedures for **13**, a solution of 2 *N* hydrochloric acid in ether (1.06 mL, 2.1 mmol) was added to a solution of compound **34** (200 mg, 0.42 mmol) in dichloromethane/methanol (2:1, 1 mL), and the resulting mixture was stirred at room temperature for 4 h. After concentration *in vacuo*, the residue was dissolved in DMF/dichloromethane (1:3, 4 mL), and then, triethylamine (0.35 mL, 2.52 mmol), 4-chlorobenzoic acid (78 mg, 0.5 mmol), and propanephosphonic acid anhydride (T3P) ≥50 wt % in ethyl acetate (400 mg, 0.63 mmol) were added. The solution was stirred at room temperature for 16 h. After workup, the crude residue was purified by silica gel column chromatography with ethyl acetate/methanol (85:15) to afford compound **7** (144 mg, 0.28 mmol, 67% yield) as a yellow solid. ^1^H NMR (400 MHz, CDCl_3_): δ 7.42 and 7.42 (d, *J* = 8.4 Hz, 2H), 7.36 and 7.34 (d, *J* = 8.4 Hz, 2H), 5.91 and 5.85 (s, 1H), 5.62 and 5.57 (s, 1H), 4.69 and 4.56 (br s, 1H), 4.20–4.14 (m, 1H), 3.77–3.66 (m, 2H), 3.64–3.40 (m, 8H), 2.50–2.40 (m, 6H), 2.29 and 2.28 (s, 3H), 2.26–2.17 (m, 1H), 2.14–2.05 (m, 1H), 1.12 and 1.11 (t, *J* = 7.2 Hz, 3H); ^13^C NMR (100 MHz, DMSO-*d*_6_): δ 167.46, 163.52, 163.25, 161.03 × 2, 160.84, 135.68 and 135.64, 134.57, 129.19, 128.37 and 128.31, 75.52, 54.84 and 52.16, 52.02 × 2, 51.78, 51.21 and 49.53, 47.31 and 44.56, 43.76, 31.81 and 29.42, 11.94 × 2; LCMS (ESI) *m*/*z*: 510.2 [M + H]^+^; HRMS (ESI) calcd for C_25_H_32_ClN_9_O [M + H]^+^*m*/*z*: 510.2497; found, 510.2498; HPLC purity = 99.63%, *t*R = 10.60 min.

#### (*S*)-(2,4-Dichlorophenyl) (3-((4-(4-ethylpiperazin-1-yl)-6-((5-methyl-1*H*-pyrazol-3-yl)amino)pyrimidin-2-yl)amino)pyrrolidin-1-yl)methanone (**8**)

Similar to the reaction procedures for **13**, a solution of 2 *N* hydrochloric acid in ether (1.06 mL, 2.1 mmol) was added to a solution of compound **34** (200 mg, 0.42 mmol) in dichloromethane/methanol (2:1, 1 mL), and the resulting mixture was stirred at room temperature for 4 h. After concentration *in vacuo*, the residue was dissolved in DMF/dichloromethane (1:3, 4 mL), and then, triethylamine (0.35 mL, 2.52 mmol), 2,4-dichlorobenzoic acid (96 mg, 0.5 mmol), and propanephosphonic acid anhydride (T3P) ≥50 wt % in ethyl acetate (400 mg, 0.63 mmol) were added. The solution was stirred at room temperature for 16 h. After workup, the crude residue was purified by silica gel column chromatography with ethyl acetate/methanol (85:15) to afford compound **8** (101 mg, 0.18 mmol, 44% yield) as a yellow solid. ^1^H NMR (400 MHz, CDCl_3_): δ 7.42 and 7.41 (d, *J* = 11.8 Hz, 1H), 7.25–7.23 (m, 1H), 7.22 and 7.20 (t, *J* = 7.6 Hz, 1H), 5.91 and 5.86 (s, 1H), 5.65 and 5.64 (s, 1H), 4.57 (br s, 1H), 4.11–4.00 (m, 1H), 3.80–3.68 (m, 1H), 3.63–3.47 (m, 5H), 3.45–3.14 (m, 2H), 2.51–2.40 (m, 6H), 2.29 and 2.28 (s, 3H), 2.27–2.02 (m, 2H), 1.12 (t, *J* = 7.6 Hz, 3H); ^13^C NMR (175 MHz, DMSO-*d*_6_): δ 164.60, 163.41, 163.14, 160.92 × 2, 160.70, 136.04 and 135.88, 134.16 and 136.12, 130.15 and 130.04, 129.26 and 129.21, 129.09 and 129.00, 127.87, 75.47, 53.16 and 51.17, 52.12, 51.99, 51.69, 50.71 and 49.62, 45.85 and 43.72, 43.67, 31.19 and 29.41, 11.95 × 2; LCMS (ESI) *m*/*z*: 544.2 [M + H]^+^; HRMS (ESI) calcd for C_25_H_31_Cl_2_N_9_O [M + H]^+^*m*/*z*: 544.2107; found, 544.2094; HPLC purity = 99.97%, *t*R = 12.04 min.

#### (*S*)-(4-Chloro-2-methylphenyl) (3-((4-(4-ethylpiperazin-1-yl)-6-((5-methyl-1*H*-pyrazol-3-yl)amino)pyrimidin-2-yl)amino)pyrrolidin-1-yl)methanone (**9**)

Similar to the reaction procedures for **13**, a solution of 2 *N* hydrochloric acid in ether (1.06 mL, 2.1 mmol) was added to a solution of compound **34** (200 mg, 0.42 mmol) in dichloromethane/methanol (2:1, 1 mL), and the resulting mixture was stirred at room temperature for 4 h. After concentration *in vacuo*, the residue was dissolved in DMF/dichloromethane (1:3, 4 mL), and then, triethylamine (0.35 mL, 2.52 mmol), 4-chloro-2-methylbenzoic acid (85 mg, 0.5 mmol), and propanephosphonic acid anhydride (T3P) ≥50 wt % in ethyl acetate (400 mg, 0.63 mmol) were added. The solution was stirred at room temperature for 16 h. After workup, the crude residue was purified by silica gel column chromatography with ethyl acetate/methanol (90:10) to afford compound **9** (160 mg, 0.31 mmol, 68% yield) as a yellow solid. ^1^H NMR (400 MHz, CDCl_3_): δ 7.22–7.07 (m, 3H), 5.91 and 5.86 (s, 1H), 5.66 (s, 1H), 4.55 (br s, 1H), 4.15–4.05 (m, 1H), 3.79–3.32 (m, 7H), 3.20–3.13 (m, 1H), 2.54–2.40 (m, 6H), 2.30–2.25 (m, 6H), 2.15–2.05 (m, 2H), 1.13 and 1.12 (t, *J* = 7.0 Hz, 3H); ^13^C NMR (150 MHz, DMSO-*d*_6_): δ 168.04, 163.90, 163.65, 161.42 × 2, 161.19, 137.19 and 137.00, 136.80 and 136.60, 133.38 and 133.33, 130.32 and 130.25, 127.93 and 127.84, 126.17, 75.95, 54.00 and 51.50, 52.52, 52.39, 52.09, 51.25 and 50.12, 46.61 and 43.82, 44.18, 44.14, 40.58, 31.79 and 29.99, 18.83, 18.68, 12.32; LCMS (ESI) *m*/*z*: 524.3 [M + H]^+^; HRMS (ESI) calcd for C_26_H_34_ClN_9_O [M + H]^+^*m*/*z*: 524.2653; found, 524.2655; HPLC purity = 99.65%, *t*R = 11.29 min.

#### (*S*)-(4-Chloro-3-fluorophenyl) (3-((4-(4-ethylpiperazin-1-yl)-6-((5-methyl-1*H*-pyrazol-3-yl)amino)pyrimidin-2-yl)amino)pyrrolidin-1-yl)methanone (**10**)

Similar to the reaction procedures for **13**, a solution of 2 *N* hydrochloric acid in ether (1.06 mL, 2.1 mmol) was added to a solution of compound **34** (200 mg, 0.42 mmol) in dichloromethane/methanol (2:1, 1 mL), and the resulting mixture was stirred at room temperature for 4 h. After concentration *in vacuo*, the residue was dissolved in DMF/dichloromethane (1:3, 4 mL), and then, triethylamine (0.35 mL, 2.52 mmol), 4-chloro-3-fluorobenzoic acid (87 mg, 0.5 mmol), and propanephosphonic acid anhydride (T3P) ≥50 wt % in ethyl acetate (400 mg, 0.63 mmol) were added. The solution was stirred at room temperature for 16 h. After workup, the crude residue was purified by silica gel column chromatography with ethyl acetate/methanol (85:15) to afford compound **10** (122 mg, 0.23 mmol, 55% yield) as a yellow solid. ^1^H NMR (700 MHz, DMSO-*d*_6_): δ 8.70 (br s, 1H), 7.68 and 7.64 (t, *J* = 8.1 Hz, 1H), 7.60 and 7.57 (dd, *J* = 9.8, 1.4 Hz, 1H), 6.51 (br s, 1H), 6.06 (br s, 1H), 5.80 (br s, 1H), 4.41 and 4.20 (br s, 1H), 3.85–3.73 (m, 1H), 3.67–3.62 (m, 1H), 3.61–3.50 (m, 1H), 3.50–3.40 (m, 3H), 2.49–2.22 (m, 6H), 2.16 and 2.13 (s, 3H), 2.11–2.09 (m, 1H), 2.03–1.86 (m, 1H), 1.07–0.99 (m, 3H); ^13^C NMR (175 MHz, DMSO-*d*_6_): δ 166.00, 165.96, 163.35, 163.08, 160.89 × 2, 156.80 and 156.76 (d, *J*_*C–F*_ = 245.9 Hz), 137.83 and 137.80 (d, *J*_*C–F*_ = 12.3 Hz), 130.69 (d, *J*_*C–F*_ = 10.5 Hz), 124.53, 120.95 and 120.92 (d, *J*_*C–F*_ = 17.3 Hz), 115.78 (d, *J*_*C–F*_ = 22.0 Hz), 75.56, 54.54 and 51.11, 51.96, 51.84, 51.63, 49.37 and 49.09, 44.39 and 40.00, 43.53, 31.69 and 29.37, 11.76 × 2; LCMS (ESI) *m*/*z*: 528.2 [M + H]^+^; HRMS (ESI) calcd for C_25_H_31_ClFN_9_O [M + H]^+^*m*/*z*: 528.2402; found, 528.2400; HPLC purity = 98.79%, *t*R = 11.67 min.

#### (*S*)-(4-Chloro-3-methylphenyl) (3-((4-(4-ethylpiperazin-1-yl)-6-((5-methyl-1*H*-pyrazol-3-yl)amino)pyrimidin-2-yl)amino)pyrrolidin-1-yl)methanone (**11**)

Similar to the reaction procedures for **13**, a solution of 2 *N* hydrochloric acid in ether (1.06 mL, 2.1 mmol) was added to a solution of compound **34** (200 mg, 0.42 mmol) in dichloromethane/methanol (2:1, 1 mL), and the resulting mixture was stirred at room temperature for 4 h. After concentration *in vacuo*, the residue was dissolved in DMF/dichloromethane (1:3, 4 mL), and then, triethylamine (0.35 mL, 2.52 mmol), 4-chloro-3-methylbenzoic acid (85 mg, 0.5 mmol), and propanephosphonic acid anhydride (T3P) ≥50 wt % in ethyl acetate (400 mg, 0.63 mmol) were added. The solution was stirred at room temperature for 16 h. After workup, the crude residue was purified by silica gel column chromatography with ethyl acetate/methanol (90:10) to afford compound **11** (170 mg, 0.32 mmol, 60% yield) as a yellow solid. ^1^H NMR (400 MHz, CDCl_3_): δ 7.36–7.30 (m, 2H), 7.23 and 7.23 (d, *J* = 8.0 Hz, 1H), 5.91 and 5.84 (s, 1H), 5.641 and 5.61 (s, 1H), 4.65 and 4.56 (br s, 1H), 4.12–4.00 (m, 1H), 3.80–3.70 (m, 2H), 3.65–3.40 (m, 6H), 2.53–2.40 (m, 6H), 2.36 (s, 3H), 2.29 and 2.28 (s, 3H), 2.25–2.16 (m, 1H), 2.15–2.03 (m, 1H), 1.12 and 1.11 (t, *J* = 7.2 Hz, 3H); ^13^C NMR (150 MHz, DMSO-*d*_6_): δ 167.39 × 2, 163.38, 163.13, 160.87, 160.68, 135.77, 135.46 and 135.39, 134.47, 129.83, 128.59 and 128.53, 126.23, 75.40, 54.60 and 52.01, 51.87, 51.81, 51.58, 51.05 and 49.38, 47.12 and 44.17, 43.65, 31.64 and 29.30, 19.34, 11.80; LCMS (ESI) *m*/*z*: 524.3 [M + H]^+^; HRMS (ESI) calcd for C_26_H_34_ClN_9_O [M + H]^+^*m*/*z*: 524.2653; found, 524.2653; HPLC purity = 97.33%, *t*R = 11.61 min.

#### (*S*)-(5-Chloro-2-fluorophenyl) (3-((4-(4-ethylpiperazin-1-yl)-6-((5-methyl-1*H*-pyrazol-3-yl)amino)pyrimidin-2-yl)amino)pyrrolidin-1-yl)methanone (**12**)

Similar to the reaction procedures for **13**, a solution of 2 *N* hydrochloric acid in ether (1.06 mL, 2.1 mmol) was added to a solution of compound **34** (200 mg, 0.42 mmol) in dichloromethane/methanol (2:1, 1 mL), and the resulting mixture was stirred at room temperature for 4 h. After concentration *in vacuo*, the residue was dissolved in DMF/dichloromethane (1:3, 4 mL), and then, triethylamine (0.35 mL, 2.52 mmol), 5-chloro-2-fluorobenzoic acid (87 mg, 0.5 mmol), and propanephosphonic acid anhydride (T3P) ≥50 wt % in ethyl acetate (400 mg, 0.63 mmol) were added. The solution was stirred at room temperature for 16 h. After workup, the crude residue was purified by silica gel column chromatography with ethyl acetate/methanol (90:10) to afford compound **12** (162 mg, 0.31 mmol, 68% yield) as a yellow solid. ^1^H NMR (400 MHz, CDCl_3_): δ 7.39–7.30 (m, 2H), 7.06 and 7.03 (t, *J* = 8.8 Hz, 1H), 5.89 and 5.85 (s, 1H), 5.73 and 5.71 (s, 1H), 4.56 (br s, 1H), 3.99–3.80 (m, 1H), 3.80–3.50 (m, 6H), 3.46–3.24 (m, 2H), 2.62–2.45 (m, 6H), 2.31 and 2.29 (s, 3H), 2.23–2.15 (m, 2H), 1.17 and 1.15 (t, *J* = 7.2 Hz, 3H); ^13^C NMR (150 MHz, DMSO-*d*_6_): δ 163.67, 163.49, 162.81, 161.33 × 2, 161.25, 156.88 and 156.83 (d, *J*_*C–F*_ = 245.6 Hz), 131.57, 129.04, 128.70, 127.60 and 127.51 (d, *J*_*C–F*_ = 20.1 Hz), 118.47 and 118.40 (d, *J*_*C–F*_ = 23.6 Hz), 76.11, 53.56 and 51.90, 51.76 × 3, 51.15 and 50.03, 46.39 and 44.38, 43.26, 31.61 and 29.98, 11.37 × 2; LCMS (ESI) *m*/*z*: 528.2 [M + H]^+^; HRMS (ESI) calcd for C_25_H_31_ClFN_9_O [M + H]^+^*m*/*z*: 528.2402; found, 528.2410; HPLC purity = 97.89%, *t*R = 10.86 min.

#### (*S*)-(3-((4-(4-Ethylpiperazin-1-yl)-6-((5-methyl-1*H*-pyrazol-3-yl)amino)pyrimidin-2-yl)amino)pyrrolidin-1-yl) (2-fluoro-4-(trifluoromethyl)phenyl)methanone (**14**)

Similar to the reaction procedures for **13**, a solution of 2 *N* hydrochloric acid in ether (1.06 mL, 2.1 mmol) was added to a solution of compound **34** (200 mg, 0.42 mmol) in dichloromethane/methanol (2:1, 1 mL), and the resulting mixture was stirred at room temperature for 4 h. After concentration *in vacuo*, the residue was dissolved in DMF/dichloromethane (1:3, 4 mL), and then, triethylamine (0.35 mL, 2.52 mmol), 2-fluoro-4-(trifluoromethyl)benzoic acid (104 mg, 0.5 mmol), and propanephosphonic acid anhydride (T3P) ≥50 wt % in ethyl acetate (400 mg, 0.63 mmol) were added. The solution was stirred at room temperature for 16 h. After workup, the crude residue was purified by silica gel column chromatography with ethyl acetate/methanol (85:15) to afford compound **14** (129 mg, 0.23 mmol, 55% yield) as a yellow solid. ^1^H NMR (300 MHz, CD_3_OD): δ 7.74–7.55 (m, 3H), 5.87 and 5.82 (s, 1H), 4.56 and 4.42 (br s, 1H), 4.01–3.77 (m, 1H), 3.77–3.62 (m, 4H), 3.62–3.57 (m, 3H), 3.57–3.40 (m, 1H), 2.83–2.57 (m, 6H), 2.40–2.30 (m, 1H), 2.24 and 2.22 (s, 3H), 2.15–2.02 (m, 1H), 1.27–1.14 (m, 3H); ^13^C NMR (100 MHz, DMSO-*d*_6_): δ 163.24, 163.05, 162.70, 169.94 × 2, 160.82, 157.54 (d, *J*_*C–F*_ = 247.0 Hz), 131.56 (q, *J*_*C–F*_ = 31.2 Hz), 130.21, 129.45 (d, *J*_*C–F*_ = 17.6 Hz), 123.20 (q, *J*_*C–F*_ = 247.8 Hz), 121.97, 113.72 (d, *J*_*C–F*_ = 25.9 Hz), 75.66, 53.23 and 51.56, 51.37 × 3, 50.79 and 49.66, 46.03 and 44.09, 42.80, 31.18 and 29.54, 10.95 × 2; LCMS (ESI) *m*/*z*: 562.3 [M + H]^+^; HRMS (ESI) calcd for C_26_H_31_F_4_N_9_O [M + H]^+^*m*/*z*: 562.2666; found, 562.2659; HPLC purity = 98.61%, *t*R = 11.59 min.

#### (*S*)-(4-Chloro-2,6-difluorophenyl) (3-((4-(4-ethylpiperazin-1-yl)-6-((5-methyl-1*H*-pyrazol-3-yl)amino)pyrimidin-2-yl)amino)pyrrolidin-1-yl)methanone (**15**)

Similar to the reaction procedures for **13**, a solution of 2 *N* hydrochloric acid in ether (1.06 mL, 2.1 mmol) was added to a solution of compound **34** (200 mg, 0.42 mmol) in dichloromethane/methanol (2:1, 1 mL), and the resulting mixture was stirred at room temperature for 4 h. After concentration *in vacuo*, the residue was dissolved in DMF/dichloromethane (1:3, 4 mL), and then, triethylamine (0.35 mL, 2.52 mmol), 4-chloro-2,6-difluorobenzoic acid (96 mg, 0.5 mmol), and propanephosphonic acid anhydride (T3P) ≥50 wt % in ethyl acetate (400 mg, 0.63 mmol) were added. The solution was stirred at room temperature for 16 h. After workup, the crude residue was purified by silica gel column chromatography with ethyl acetate/methanol (85:15) to afford compound **15** (140 mg, 0.26 mmol, 61% yield) as a yellow solid. ^1^H NMR (400 MHz, CDCl_3_): δ 7.10–6.95 (m, 2H), 5.88 and 5.85 (s, 1H), 5.76 and 5.74 (s, 1H), 4.58 (br s, 1H), 4.21–3.87 (m, 2H), 3.80–3.68 (m, 1H), 3.67–3.47 (m, 5H), 3.43–3.17 (m, 1H), 2.57–2.43 (m, 6H), 2.38–2.30 (m, 1H), 2.29 and 2.28 (s, 3H), 2.24–2.25 (m, 1H), 1.14 and 1.14 (t, *J* = 7.2 Hz, 3H); ^13^C NMR (175 MHz, DMSO-*d*_6_): δ 163.57, 163.35, 161.23, 161.02, 158.65, 158.56, 158.27 (t, *J*_*C–F*_ = 247.4 Hz), 135.72 (t, *J*_*C–F*_ = 12.8 Hz), 114.12 (q, *J*_*C–F*_ = 23.1 Hz), 113.72 (d, *J*_*C–F*_ = 23.3 Hz), 75.89, 53.12 and 50.84, 51.84 × 3, 50.01 and 45.86, 44.34 and 40.00, 43.39, 31.01 and 29.66, 11.57, 11.49; LCMS (ESI) *m*/*z*: 546.2 [M + H]^+^; HRMS (ESI) calcd for C_25_H_30_ClF_2_N_9_O [M + H]^+^*m*/*z*: 546.2308; found, 546.2314; HPLC purity = 99.75%, *t*R = 11.23 min.

#### (*S*)-(4-Chloro-2,5-difluorophenyl) (3-((4-(4-ethylpiperazin-1-yl)-6-((5-methyl-1*H*-pyrazol-3-yl)amino)pyrimidin-2-yl)amino)pyrrolidin-1-yl)methanone (**16**)

Similar to the reaction procedures for **13**, a solution of 2 *N* hydrochloric acid in ether (1.06 mL, 2.1 mmol) was added to a solution of compound **34** (200 mg, 0.42 mmol) in dichloromethane/methanol (2:1, 1 mL), and the resulting mixture was stirred at room temperature for 4 h. After concentration *in vacuo*, the residue was dissolved in DMF/dichloromethane (1:3, 4 mL), and then, triethylamine (0.35 mL, 2.52 mmol), 4-chloro-2,5-difluorobenzoic acid (96 mg, 0.5 mmol), and propanephosphonic acid anhydride (T3P) ≥50 wt % in ethyl acetate (400 mg, 0.63 mmol) were added. The solution was stirred at room temperature for 16 h. After workup, the crude residue was purified by silica gel column chromatography with ethyl acetate/methanol (85:15) to afford compound **16** (119 mg, 0.22 mmol, 52% yield) as a yellow solid. ^1^H NMR (400 MHz, CDCl_3_): δ 7.23–7.10 (m, 2H), 5.91 and 5.86 (s, 1H), 5.70 and 5.68 (s, 1H), 4.63 and 4.58 (br s, 1H), 4.02–3.95 (m, 1H), 3.80–3.70 (m, 2H), 3.65–3.47 (m, 5H), 3.44–3.25 (m, 1H), 2.55–2.42 (m, 6H), 2.30 and 2.29 (s, 3H), 2.22–2.03 (m, 2H), 1.14 and 1.13 (t, *J* = 7.2 Hz, 3H); ^13^C NMR (100 MHz, DMSO-*d*_6_): δ 163.37, 163.12, 161.78, 160.93, 160.79 × 2, 153.81 (d, *J*_*C–F*_ = 242.5 Hz), 153.48 (d, *J*_*C–F*_ = 244.8 Hz), 125.72, 121.46, 118.63 and 118.57 (d, *J*_*C–F*_ = 28.2 Hz), 116.46 and 116.26, 76.50, 53.24 and 51.81, 51.74, 51.59 × 2, 50.75 and 49.61, 46.00 and 44.05, 43.38, 31.17 and 29.20, 11.57 × 2; LCMS (ESI) *m*/*z*: 546.2 [M + H]^+^; HRMS (ESI) calcd for C_25_H_30_ClF_2_N_9_O [M + H]^+^*m*/*z*: 546.2308; found, 546.2312; HPLC purity = 97.98%, *t*R = 11.07 min.

#### (*S*)-(4-Chloro-2,3-difluorophenyl) (3-((4-(4-ethylpiperazin-1-yl)-6-((5-methyl-1*H*-pyrazol-3-yl)amino)pyrimidin-2-yl)amino)pyrrolidin-1-yl)methanone (**17**)

Similar to the reaction procedures for **13**, a solution of 2 *N* hydrochloric acid in ether (1.06 mL, 2.1 mmol) was added to a solution of compound **34** (200 mg, 0.42 mmol) in dichloromethane/methanol (2:1, 1 mL), and the resulting mixture was stirred at room temperature for 4 h. After concentration *in vacuo*, the residue was dissolved in DMF/dichloromethane (1:3, 4 mL), and then, triethylamine (0.35 mL, 2.52 mmol), 4-chloro-2,3-difluorobenzoic acid (96 mg, 0.5 mmol), and propanephosphonic acid anhydride (T3P) ≥50 wt % in ethyl acetate (400 mg, 0.63 mmol) were added. The solution was stirred at room temperature for 16 h. After workup, the crude residue was purified by silica gel column chromatography with ethyl acetate/methanol (85:15) to afford compound **17** (101 mg, 0.18 mmol, 44% yield) as a yellow solid. ^1^H NMR (700 MHz, CDCl_3_): δ 7.18 (t, *J* = 7.0 Hz, 1H), 7.04 (s, 1H), 5.93 and 5.87 (s, 1H), 5.63 and 5.60 (s, 1H), 4.70 and 4.58 (br s, 1H), 4.11 (m, 1H), 3.79–3.68 (m, 2H), 3.60–3.57 (m, 1H), 3.56–3.46 (m, 4H), 3.41–3.27 (m, 1H), 2.50–2.41 (m, 6H), 2.30 and 2.29 (s, 3H), 2.27–2.22 (m, 1H), 2.16–2.08 (m, 1H), 1.12 and 1.11 (t, *J* = 7.0 Hz, 3H); ^13^C NMR (175 MHz, DMSO-*d*_6_): δ 164.82 × 2, 164.20, 164.17, 161.03, 147.78 and 147.33 (dd, *J*_*C–F*_ = 237.0, 15.2 Hz), 147.42 (dd, *J*_*C–F*_ = 237.6, 13.8 Hz), 126.09, 125.79 and 125.71 (dd, *J*_*C–F*_ = 48.6, 14.4 Hz), 124.41 and 124.33 (dd, *J*_*C–F*_ = 60.9, 14.3 Hz), 123.75, 123.04, 75.03, 55.23 and 51.13, 52.73, 52.67, 52.63, 51.79 and 50.64, 46.35 and 45.27, 44.36, 32.62 and 30.73, 12.12, 11.73; LCMS (ESI) *m*/*z*: 546.2 [M + H]^+^; HRMS (ESI) calcd for C_25_H_30_ClF_2_N_9_O [M + H]^+^*m*/*z*: 546.2308; found, 546.2300; HPLC purity = 99.16%, *t*R = 11.82 min.

#### (*S*)-(4-Chloro-3,5-difluorophenyl) (3-((4-(4-ethylpiperazin-1-yl)-6-((5-methyl-1*H*-pyrazol-3-yl)amino)pyrimidin-2-yl)amino)pyrrolidin-1-yl)methanone (**18**)

Similar to the reaction procedures for **13**, a solution of 2 *N* hydrochloric acid in ether (1.06 mL, 2.1 mmol) was added to a solution of compound **34** (200 mg, 0.42 mmol) in dichloromethane/methanol (2:1, 1 mL), and the resulting mixture was stirred at room temperature for 4 h. After concentration *in vacuo*, the residue was dissolved in DMF/dichloromethane (1:3, 4 mL), and then, triethylamine (0.35 mL, 2.52 mmol), 4-chloro-3,5-difluorobenzoic acid (96 mg, 0.5 mmol), and propanephosphonic acid anhydride (T3P) ≥50 wt % in ethyl acetate (400 mg, 0.63 mmol) were added. The solution was stirred at room temperature for 16 h. After workup, the crude residue was purified by silica gel column chromatography with ethyl acetate/methanol (90:10) to afford compound **18** (138 mg, 0.25 mmol, 56% yield) as a yellow solid. ^1^H NMR (400 MHz, CDCl_3_): δ 7.18 and 7.15 (d, *J* = 7.0 Hz, 2H), 5.88 and 5.84 (s, 1H), 5.76 and 5.73 (s, 1H), 4.57 (br s, 1H), 4.01–3.85 (m, 1H), 3.82–3.53 (m, 6H), 3.52–3.40 (m, 2H), 2.58–2.45 (m, 6H), 2.30 and 2.29 (s, 3H), 2.20–2.15 (m, 2H), 1.20–1.10 (m, 3H); ^13^C NMR (150 MHz, DMSO-*d*_6_): δ 165.44, 163.66, 163.45, 161.29 × 3, 158.18 and 158.14 (d, *J*_*C–F*_ = 248.9 Hz), 138.18 (t, *J*_*C–F*_ = 6.8 Hz), 112.06 (d, *J*_*C–F*_ = 23.7 Hz), 110.11 (t, *J*_*C–F*_ = 20.2 Hz), 76.06, 54.71, 52.40 and 51.55, 51.74 × 2, 49.84 and 47.43, 44.88 and 43.30, 43.30, 32.08 and 29.91, 11.84, 11.33; LCMS (ESI) *m*/*z*: 546.2 [M + H]^+^; HRMS (ESI) calcd for C_25_H_30_ClF_2_N_9_O [M + H]^+^*m*/*z*: 546.2308; found, 546.2306; HPLC purity = 98.78%, *t*R = 12.07 min.

#### (*S*)-(3-((4-(4-Ethylpiperazin-1-yl)-6-((5-methyl-1*H*-pyrazol-3-yl)amino)pyrimidin-2-yl)amino)pyrrolidin-1-yl) (2,4,5-trifluorophenyl)methanone (**19**)

Similar to the reaction procedures for **13**, a solution of 2 *N* hydrochloric acid in ether (1.06 mL, 2.1 mmol) was added to a solution of compound **34** (200 mg, 0.42 mmol) in dichloromethane/methanol (2:1, 1 mL), and the resulting mixture was stirred at room temperature for 4 h. After concentration *in vacuo*, the residue was dissolved in DMF/dichloromethane (1:3, 4 mL), and then, triethylamine (0.35 mL, 2.52 mmol), 2,4,5-trifluorobenzoic acid (88 mg, 0.5 mmol), and propanephosphonic acid anhydride (T3P) ≥50 wt % in ethyl acetate (400 mg, 0.63 mmol) were added. The solution was stirred at room temperature for 16 h. After workup, the crude residue was purified by silica gel column chromatography with ethyl acetate/methanol (85:15) to afford compound **19** (100 mg, 0.19 mmol, 45% yield) as a yellow solid. ^1^H NMR (400 MHz, CDCl_3_): δ 7.33–7.23 (m, 1H), 7.01–6.92 (m, 1H), 5.87 and 5.84 (s, 1H), 5.74 and 5.72 (s, 1H), 4.54 (br s, 1H), 3.96–3.80 (m, 2H), 3.78–3.50 (m, 5H), 3.49–3.26 (m, 2H), 2.60–2.50 (m, 6H), 2.31 and 2.30 (s, 3H), 2.22–2.03 (m, 2H), 1.17 and 1.16 (t, *J* = 7.0 Hz, 3H); ^13^C NMR (175 MHz, DMSO-*d*_6_): δ 172.74, 163.37, 163.19, 162.41, 161.17, 161.07, 153.62 (dt, *J*_*C–F*_ = 244.9, 8.6 Hz), 150.14 (dt, *J*_*C–F*_ = 250.0, 13.4 Hz), 146.46 (dt, *J*_*C–F*_ = 244.3, 12.7 Hz), 122.14 (t, *J* = 21.8 Hz), 117.20 (d, *J* = 20.3 Hz), 107.08 (ddd, *J* = 29.2, 21.4, 11.1 Hz), 75.97, 53.55, 51.92 and 50.98, 51.61, 51.25, 49.91 and 46.37, 44.45 and 42.60, 42.51, 31.36 and 29.76, 10.66, 10.55; LCMS (ESI) *m*/*z*: 530.3 [M + H]^+^; HRMS (ESI) calcd for C_25_H_30_F_3_N_9_O [M + H]^+^*m*/*z*: 530.2604; found, 530.2617; HPLC purity = 96.24%, *t*R = 10.37 min.

#### (*S*)-(6-Chloro-2-fluoropyridin-3-yl) (3-((4-(4-ethylpiperazin-1-yl)-6-((5-methyl-1*H*-pyrazol-3-yl)amino)pyrimidin-2-yl)amino)pyrrolidin-1-yl)methanone (**20**)

Similar to the reaction procedures for **13**, a solution of 2 *N* hydrochloric acid in ether (1.06 mL, 2.1 mmol) was added to a solution of compound **34** (200 mg, 0.42 mmol) in dichloromethane/methanol (2:1, 1 mL), and the resulting mixture was stirred at room temperature for 4 h. After concentration *in vacuo*, the residue was dissolved in DMF/dichloromethane (1:3, 4 mL), and then, triethylamine (0.35 mL, 2.52 mmol), 6-chloro-2-fluoropyridine-3-carboxylic acid (87 mg, 0.5 mmol), and propanephosphonic acid anhydride (T3P) ≥50 wt % in ethyl acetate (400 mg, 0.63 mmol) were added. The solution was stirred at room temperature for 16 h. After workup, the crude residue was purified by silica gel column chromatography with ethyl acetate/methanol (85:15) to afford compound **20** (102 mg, 0.19 mmol, 46% yield) as a yellow solid. ^1^H NMR (400 MHz, CDCl_3_): δ 7.82 and 7.82 (t, *J* = 8.2 Hz, 1H), 7.29–7.24 (m, 1H), 5.90 and 5.85 (s, 1H), 5.68 and 5.66 (s, 1H), 4.63 and 4.59 (br s, 1H), 4.04–3.93 (m, 1H), 3.83–3.72 (m, 2H), 3.63–3.26 (m, 6H), 2.53–2.42 (m, 6H), 2.31 and 2.30 (s, 3H), 2.22–2.04 (m, 2H), 1.13 and 1.13 (t, *J* = 7.2 Hz, 3H); ^13^C NMR (175 MHz, DMSO-*d*_6_): δ 163.69, 163.45, 162.03, 162.01, 161.23, 161.03, 157.25 and 157.13 (d, *J*_*C–F*_ = 242.2 Hz), 148.26 (t, *J*_*C–F*_ = 12.1 Hz), 143.65 (d, *J*_*C–F*_ = 12.8 Hz), 123.02, 118.76 and 118.67 (d, *J*_*C–F*_ = 31.0 Hz), 75.79, 53.59 and 52.05, 52.21, 52.12, 51.96, 51.09 and 49.89, 46.35 and 44.52, 43.75, 31.43 and 29.73, 11.90 × 2; LCMS (ESI) *m*/*z*: 529.2 [M + H]^+^; HRMS (ESI) calcd for C_24_H_30_ClFN_10_O [M + H]^+^*m*/*z*: 529.2355; found, 529.2353; HPLC purity = 97.47%, *t*R = 9.99 min.

#### (*S*)-*N*^2^-(1-((4-Chloro-2-fluorophenyl)sulfonyl)pyrrolidin-3-yl)-6-(4-ethylpiperazin-1-yl)-*N*^4^-(5-methyl-1*H*-pyrazol-3-yl)pyrimidine-2,4-diamine (**21**)

A solution of 2 *N* hydrochloric acid in ether (1.06 mL, 2.1 mmol) was added to a solution of compound **34** (200 mg, 0.42 mmol) in dichloromethane/methanol (2:1, 1 mL) at room temperature. The resulting mixture was stirred at room temperature for 4 h and then concentrated to give crude amine salt, which was used without further purification.

Triethylamine (0.35 mL, 2.52 mmol) and 4-chloro-2-fluorobenzenesulfonyl chloride (114 mg, 0.5 mmol) were added to a solution of amine salt in dichloromethane (4 mL) at room temperature. The resulting mixture was stirred at room temperature for 4 h and then quenched with brine (20 mL). The aqueous phase was extracted with ethyl acetate (3 × 30 mL). The combined organic extracts were washed with water and brine, dried over magnesium sulfate, and filtered. The filtrate was concentrated to get a crude residue, which was purified by flash column chromatography over silica gel with ethyl acetate/methanol (85:15) to afford compound **21** (201 mg, 0.36 mmol, 85% yield) as a light-purple solid. ^1^H NMR (300 MHz, CD_3_OD): δ 7.81 and 7.80 (t, *J* = 7.5 Hz, 1H), 7.34–7.28 (m, 2H), 5.82 (s, 1H), 5.57 (s, 1H), 4.27 (br s, 1H), 3.80–3.66 (m, 4H), 3.63–3.45 (m, 5H), 3.02–2.80 (m, 6H), 2.26 (s, 3H), 2.23–2.15 (m, 1H), 2.04–1.93 (m, 1H), 1.28 and 1.27 (t, *J* = 7.1 Hz, 3H); ^13^C NMR (175 MHz, DMSO-*d*_6_): δ 162.84 × 2, 160.96 × 3, 158.37 (d, *J*_*C–F*_ = 255.5 Hz), 139.33 (d, *J*_*C–F*_ = 9.6 Hz), 132.27, 125.45 (d, *J*_*C–F*_ = 2.6 Hz), 124.09 (d, *J*_*C–F*_ = 15.3 Hz), 118.36 (d, *J*_*C–F*_ = 25.9 Hz), 76.00, 64.32, 61.58, 53.24, 50.76, 46.36, 38.57, 38.53, 30.64, 7.17 × 2; LCMS (ESI) *m*/*z*: 564.2 [M + H]^+^; HRMS (ESI) calcd for C_24_H_31_ClFN_9_O_2_S [M + H]^+^*m*/*z*: 564.2072; found, 564.2063; HPLC purity = 96.34%, *t*R = 11.11 min.

### General Procedure for the Synthesis of Compounds **22–27**

The general procedure is illustrated below with compounds **25** as a specific example.

#### (*S*)-1-(5-((2-((1-(4-Chloro-2-fluorobenzoyl)pyrrolidin-3-yl)amino)-6-(4-ethylpiperazin-1-yl)pyrimidin-4-yl)amino)-3-methyl-1*H*-pyrazol-1-yl)propan-1-one (**25**)

A solution of propionic anhydride (60 mg, 0.46 mmol) in 1,4-dioxane (0.5 mL) was added to a solution of compound **13** (300 mg, 0.42 mmol) in 1,4-dioxane (6 mL) at 140 °C. The resulting mixture was stirred at 140 °C for 30 min, cooled to room temperature, and then concentrated in vacuo. The residue was purified by flash column chromatography over silica gel with *n*-hexane/ethyl acetate/triethylamine (60:35:5) to afford compound **25** (137 mg, 0.24 mmol, 56% yield) and **26** (69 mg, 0.12 mmol, 28% yield) as a pale-yellow solid. ^1^H NMR (300 MHz, CDCl_3_): δ 9.88 and 9.84 (br s, 1H), 7.34 and 7.32 (t, *J* = 7.7 Hz, 1H), 7.12 (q, *J* = 8.3 Hz, 1H), 7.05 (d, *J* = 9.3 Hz, 1H), 6.51 and 6.42 (s, 1H), 5.30 and 5.25 (s, 1H), 4.72 (br s, 1H), 4.60–4.40 (m, 1H), 4.00–3.60 (m, 3H), 3.60–3.15 (m, 5H), 3.06 and 3.05 (q, *J* = 8.3 Hz, 2H), 2.65–2.37 (m, 6H), 2.35–2.23 (m, 1H), 2.19 and 2.17 (s, 3H), 2.03–1.92 (m, 1H), 1.23–1.07 (m, 6H); ^13^C NMR (100 MHz, CDCl_3_): δ 178.03, 164.45, 163.88 and 163.77, 161.40 and 161.32, 159.13, 158.31 and 158.28 (d, *J*_*C–F*_ = 250.1 Hz), 153.91 and 153.85, 144.18 and 144.12, 136.62 (d, *J*_*C–F*_ = 9.9 Hz), 130.13 and 130.08 (d, *J*_*C–F*_ = 10.0 Hz), 125.18 and 125.15, 123.84 and 123.80 (d, *J*_*C–F*_ = 18.0 Hz), 116.82 and 116.74 (d, *J*_*C–F*_ = 25.2 Hz), 95.80 and 95.69, 78.39, 53.70 and 52.24, 52.47, 52.41, 51.20 and 50.28, 46.01 and 44.35, 44.05, 32.21 and 30.55, 29.00, 14.45, 11.95, 8.32; LCMS (ESI) *m*/*z*: 584.2 [M + H]^+^; HRMS (ESI) calcd for C_28_H_35_ClFN_9_O_2_ [M + H]^+^*m*/*z*: 584.2665; found, 584.2645; HPLC purity = 96.81%, *t*R = 16.39 min.

#### (*S*)-1-(3-((2-((1-(4-Chloro-2-fluorobenzoyl)pyrrolidin-3-yl)amino)-6-(4-ethylpiperazin-1-yl)pyrimidin-4-yl)amino)-5-methyl-1*H*-pyrazol-1-yl)propan-1-one (**26**)

^1^H NMR (400 MHz, CDCl_3_): δ 7.35 (q, *J* = 6.8 Hz, 1H), 7.15 (q, *J* = 9.7 Hz, 1H), 7.09 (d, *J* = 9.6 Hz, 1H), 6.35 and 6.30 (s, 1H), 6.04 and 6.00 (s, 1H), 5.03 and 4.93 (br s, 1H), 4.55 and 4.44 (d, *J* = 5.7 Hz, 1H), 4.03–3.34 (m, 7H), 3.30–3.16 (m, 1H), 3.04 (t, *J* = 7.8 Hz, 2H), 2.60–2.36 (m, 5H), 2.47–2.36 (m, 4H), 2.35–2.18 (m, 2H), 1.29–1.19 (m, 3H), 1.11 (t, *J* = 7.0 Hz, 3H); ^13^C NMR (100 MHz, CDCl_3_): δ 174.26 and 174.24, 164.55, 164.36 and 164.28, 160.78, 159.64 and 159.56, 158.29 (d, *J*_*C–F*_ = 230.3 Hz), 150.78 and 150.71, 144.61, 136.72 (d, *J*_*C–F*_ = 9.9 Hz), 130.23 and 130.19 (d, *J*_*C–F*_ = 9.1 Hz), 125.27, 123.94 and 123.85 (d, *J*_*C–F*_ = 18.0 Hz), 116.91 and 116.83 (d, *J*_*C–F*_ = 25.2 Hz), 103.03 and 102.95, 78.22, 53.78 and 52.32, 52.59, 52.53, 51.15 and 50.24, 46.11 and 44.42, 44.18, 32.25 and 30.64, 28.90, 14.88, 12.02, 8.85; LCMS (ESI) *m*/*z*: 584.3 [M + H]^+^; HRMS (ESI) calcd for C_28_H_35_ClFN_9_O_2_ [M + H]^+^*m*/*z*: 584.2655; found, 584.2665; HPLC purity = 97.32%, *t*R = 12.99 min.

#### Ethyl (*S*)-5-((2-((1-(4-chloro-2-fluorobenzoyl)pyrrolidin-3-yl)amino)-6-(4-ethylpiperazin-1-yl)pyrimidin-4-yl)amino)-3-methyl-1*H*-pyrazole-1-carboxylate (**22**)

Similar to the reaction procedures for **25**, diethyl dicarbonate (375 mg, 2.3 mmol) was added to a solution of compound **13** (300 mg, 0.42 mmol) in 1,4-dioxane (6 mL). The resulting mixture was stirred at 140 °C for 30 min. After workup, the crude residue was purified by silica gel column chromatography with dichloromethane/methanol (97:3) to give compound **22** (118 mg, 0.2 mmol, 47% yield) and compound **23** (103 mg, 0.17 mmol, 41% yield) as a yellow solid. ^1^H NMR (400 MHz, CDCl_3_): δ 9.35 and 9.31 (br s, 1H), 7.38 (q, *J* = 7.3 Hz, 1H), 7.22–7.07 (m, 2H), 6.56 and 6.48 (s, 1H), 5.37 and 5.33 (s, 1H), 4.90 and 4.84 (br s, 1H), 4.62–4.44 (m, 3H), 4.04–3.68 (m, 2H), 3.66–3.45 (m, 4H), 3.43–3.21 (m, 2H), 2.57–2.40 (m, 6H), 2.38–2.29 (m, 1H), 2.27 and 2.26 (s, 3H), 2.06–1.95 (m, 1H), 1.47 and 1.46 (t, *J* = 7.2 Hz, 3H), 1.13 and 1.12 (t, *J* = 7.2 Hz, 3H); ^13^C NMR (100 MHz, CDCl_3_): δ 164.61, 164.01 and 163.90, 161.49 and 161.40, 158.45 and 158.41 (d, *J*_*C–F*_ = 7.2 Hz), 159.17 and 159.14, 154.36 and 154.31, 152.59, 144.31 and 144.25, 136.80 (d, *J*_*C–F*_ = 9.9 Hz), 130.26 and 130.21 (d, *J*_*C–F*_ = 9.6 Hz), 125.33 and 125.31, 123.95 and 123.91 (d, *J*_*C–F*_ = 17.9 Hz), 116.97 and 116.88 (d, *J*_*C–F*_ = 25.2 Hz), 95.73 and 95.64, 78.43, 64.60, 53.87 and 52.38, 52.56, 52.50, 51.33 and 50.41, 46.14 and 44.45, 44.13, 32.39 and 30.75, 14.65, 14.50, 12.01 and 11.98; LCMS (ESI) *m*/*z*: 600.3 [M + H]^+^; HRMS (ESI) calcd for C_28_H_35_ClFN_9_O_3_ [M + H]^+^*m*/*z*: 600.2614; found, 600.2612; HPLC purity = 97.55%, *t*R = 16.35 min.

#### Ethyl (*S*)-3-((2-((1-(4-chloro-2-fluorobenzoyl)pyrrolidin-3-yl)amino)-6-(4-ethylpiperazin-1-yl)pyrimidin-4-yl)amino)-5-methyl-1*H*-pyrazole-1-carboxylate (**23**)

^1^H NMR (400 MHz, CDCl_3_): δ 7.37 (q, *J* = 7.5 Hz, 1H), 7.17 (q, *J* = 10.3 Hz, 1H), 7.10 (d, *J* = 9.6 Hz, 1H), 6.31 and 6.26 (s, 1H), 5.97 and 5.89 (s, 1H), 4.98 and 4.88 (br s, 1H), 4.58–4.39 (m, 3H), 4.04–3.67 (m, 2H), 3.65–3.51 (m, 4H), 3.50–3.19 (m, 2H), 2.53 and 2.52 (s, 3H), 2.50–2.42 (m, 6H), 2.35–2.17 (m, 2H), 1.43 and 1.42 (t, *J* = 7.0 Hz, 3H), 1.13 and 1.12 (t, *J* = 7.2 Hz, 3H); ^13^C NMR (100 MHz, CDCl_3_): δ 164.61, 164.15 and 164.11, 161.02, 160.01 and 159.88, 158.44 (d, *J*_*C–F*_ = 250.8 Hz), 151.35 and 151.28, 150.51 and 150.48, 145.18, 136.78 (d, *J*_*C–F*_ = 10.7 Hz), 130.28 and 130.23 (d, *J*_*C–F*_ = 9.5 Hz), 125.32 (d, *J*_*C–F*_ = 2.2 Hz), 124.00 and 123.94 (d, *J*_*C–F*_ = 17.5 Hz), 116.97 and 116.90 (d, *J*_*C–F*_ = 25.2 Hz), 103.05 and 102.96, 77.81 and 77.73, 63.94, 53.88 and 52.38, 52.63, 52.57, 51.25 and 50.35, 46.18 and 44.47, 44.09, 32.34 and 30.73, 14.68, 14.45, 12.01 and 11.98; LCMS (ESI) *m*/*z*: 600.3 [M + H]^+^; HRMS (ESI) calcd for C_28_H_35_ClFN_9_O_3_ [M + H]^+^*m*/*z*: 600.2614; found, 600.2616; HPLC purity = 97.55%, *t*R = 16.35 min.

#### *tert*-Butyl (*S*)-5-((2-((1-(4-chloro-2-fluorobenzoyl)pyrrolidin-3-yl)amino)-6-(4-ethylpiperazin-1-yl)pyrimidin-4-yl)amino)-3-methyl-1*H*-pyrazole-1-carboxylate (**24**)

Similar to the reaction procedures for **25**, di-*tert*-butyl dicarbonate (100 mg, 0.46 mmol) was added to a solution of compound **13** (300 mg, 0.42 mmol) in 1,4-dioxane (6 mL). The resulting mixture was stirred at 140 °C for 30 min. After workup, the crude residue was purified by silica gel column chromatography with dichloromethane/methanol (97:3) to give compound **24** (90 mg, 0.14 mmol, 34% yield) as a yellow solid. ^1^H NMR (400 MHz, CDCl_3_): δ 9.44 and 9.42 (br s, 1H), 7.39 (q, *J* = 7.7 Hz, 1H), 7.24–7.09 (m, 2H), 6.55 and 6.47 (s, 1H), 5.39 and 5.35 (s, 1H), 4.83 and 4.78 (br s, 1H), 4.63–4.45 (m, 2H), 4.23–4.00 (m, 2H), 3.92–3.72 (m, 2H), 3.65–3.48 (m, 2H), 3.47–3.23 (m, 1H), 2.55–2.39 (m, 4H), 2.39–2.32 (m, 2H), 2.28 and 2.27 (s, 3H), 2.07–1.99 (m, 2H), 1.56 (s, 9H), 1.14 and 1.12 (t, *J* = 7.2 Hz, 3H); ^13^C NMR (100 MHz, DMSO-*d*_6_): δ 164.66, 164.09 and 163.96, 161.52 and 161.43, 159.26, 158.46 (d, *J*_*C–F*_ = 253.2 Hz), 153.63, 151.51, 144.34 and 144.28, 130.28 (d, *J*_*C–F*_ = 10.7 Hz), 130.28 and 130.23 (d, *J*_*C–F*_ = 9.6 Hz), 125.35, 123.97 (d, *J*_*C–F*_ = 17.6 Hz), 116.99 and 116.91 (d, *J*_*C–F*_ = 25.2 Hz), 95.56 and 95.49, 86.14, 78.40, 53.88 and 52.43, 52.66, 52.59, 51.34 and 50.43, 46.17 and 44.49, 44.25, 32.43 and 30.77, 28.26, 14.82, 12.11; LCMS (ESI) *m*/*z*: 628.3 [M + H]^+^; HRMS (ESI) calcd for C_30_H_39_ClFN_9_O_3_ [M + H]^+^*m*/*z*: 628.2927; found, 628.2922; HPLC purity = 97.10%, *t*R = 16.70 min.

#### (*S*)-1-(5-((2-((1-(4-Chloro-2-fluorobenzoyl)pyrrolidin-3-yl)amino)-6-(4-ethylpiperazin-1-yl)pyrimidin-4-yl)amino)-3-methyl-1*H*-pyrazol-1-yl)-2,2-dimethylpropan-1-one (**27**)

Similar to the reaction procedures for **25**, pivalic anhydride (86 mg, 0.46 mmol) was added to a solution of compound **13** (300 mg, 0.42 mmol) in 1,4-dioxane (6 mL). The resulting mixture was stirred at 140 °C for 30 min. After workup, the crude residue was purified by silica gel column chromatography with dichloromethane/methanol (97:3) to give compound **27** (87 mg, 0.14 mmol, 34% yield) as a yellow solid. ^1^H NMR (400 MHz, CDCl_3_): δ 7.42–7.35 (m, 1H), 7.23–7.09 (m, 2H), 6.55 and 6.50 (s, 1H), 5.87 and 5.84 (s, 1H), 4.92 and 4.79 (br s, 1H), 4.56 and 4.47 (q, *J* = 5.7 Hz, 1H), 4.06–3.79 (m, 1H), 3.77–3.55 (m, 5H), 3.54–3.19 (m, 2H), 2.56–2.40 (m, 9H), 2.36–2.22 (m, 1H), 2.03–1.94 (m, 1H), 1.53 and 1.51 (s, 9H), 1.16–1.12 (m, 3H); ^13^C NMR (100 MHz, CDCl_3_): δ 177.66 and 177.63, 164.58, 164.45 and 164.35, 160.66, 159.25 and 159.17, 158.36 (d, *J*_*C–F*_ = 250.9 Hz), 149.93 and 149.83, 145.61, 136.69 (d, *J*_*C–F*_ = 9.9 Hz), 130.19 and 130.14 (d, *J*_*C–F*_ = 9.6 Hz), 125.24, 123.88 and 123.78 (d, *J*_*C–F*_ = 17.6 Hz), 116.88 and 116.80 (d, *J*_*C–F*_ = 24.8 Hz), 101.95 and 101.85, 78.60, 53.81 and 52.27, 52.55, 52.49, 51.10 and 50.19, 46.15 and 42.07, 44.01, 44.31, 44.27, 44.14, 32.21 and 30.57, 24.79, 27.76, 27.71, 15.60, 12.00; LCMS (ESI) *m*/*z*: 612.3 [M + H]^+^; HRMS (ESI) calcd for C_30_H_39_ClFN_9_O_2_ [M + H]^+^*m*/*z*: 612.2978; found, 612.2974; HPLC purity = 97.45%, *t*R = 16.89 min.

### *In Vitro* Inhibition of Aurora A Kinase Activity

The efficacy of the compounds as inhibitors of Aurora A kinase was assessed *in vitro* using the Kinase-Glo Plus Luminescent Kinase assay (Promega, USA), as described previously.^45^ In brief, recombinant glutathione S-transferase (GST)-tagged N-terminal truncated human Aurora A (amino acids 123–401) was expressed in Sf9 insect cells and purified by glutathione affinity chromatography to obtain recombinant Aurora A. Samples of purified recombinant Aurora A (150 ng) were reacted with each test compound in 50 μL of 50 mM Tris-HCl pH 7.4, 10 mM NaCl, 10 mM MgCl_2_, 0.01% bovine serum albumin, 5.0 μM ATP, 1 mM dithiothreitol, 15 μM tetra(-LRRASLG) peptide at 37 °C for 120 min, followed by addition of 50 μL Kinase-Glo Plus Reagent. The resulting mixture was incubated at 25 °C for 20 min. A 70 μL aliquot of the mixture was transferred to a black microtiter plate. Luminescence was measured using a Wallac Vector 1420 multilabel counter (PerkinElmer, USA).

### Cell Culture

The SCLC cell lines NCI-H82, NCI-H446, NCI-H211, NCI-H524, NCI-H526, NCI-H146, NCI-H841, and NCI-H209, and the neuroblastoma cell line SK-N-BE(2) were obtained from American Type Culture Collection (ATCC, USA). All SCLC cell lines were maintained in RPMI1640 medium (ThermoFisher Scientific, USA) supplemented with 10% fetal bovine serum (FBS, Hyclone, USA) and antibiotics. SK-N-BE(2) was maintained in Minimum Essential Medium (MEM, ThermoFisher Scientific, USA) supplemented with 10% FBS (HyClone, USA) and antibiotics.

### Western Blot Analysis

Cancer cells were treated with each of the compounds at different compound concentrations. After 24 h, cells were collected, washed with 1× phosphate buffered saline (PBS), lysed in 1× Laemmli protein sample buffer, and boiled at 100 °C for 10 min. Each lysate was separated by sodium dodecyl sulfate polyacrylamide gel electrophoresis (SDS-PAGE), transferred to polyvinylidene fluoride (PVDF, Millipore, USA) membrane, and blotted with antibodies. Primary antibodies used for western blotting were cMYC (Cell Signaling, 5605S), MYCN (Cell Signaling, 9405S), PARP-1(Abcam, ab32378), GAPDH (Genetex, GTX100118), pAurora A/B/C (Cell Signaling, #2914), Aurora A (Abcam, ab52973), pHistone H3 Ser10 (Epitomics, 1173–1), Histone H3 (Millipore, 07-690), Cyclin B1 (Santa Cruz, sc-245), and β-ACTIN (Sigma-Aldrich, A1978). After the blotting (0.2% casein, 1% BSA, or 5% nonfat milk), membranes were washed with blotting buffer (0.2% casein in 1× PBS or 1× TBST), and corresponding alkaline phosphatase (AP)- or horseradish peroxidase (HRP)-conjugated secondary antibodies (Sigma-Aldrich) were added. The blots were developed by chemiluminescence (PerkinElmer, USA).

### Cell Proliferation Inhibition Assay

The efficacy of the compounds as inhibitors of cancer cell proliferation was assessed using PrestoBlue Cell Viability Reagent (ThermoFisher Scientific, USA). Cells were seeded at a density of 5000–10,000 cells per well in 96-well plates. After 24 h, cells were treated with each compound at various concentrations (0–10 μM). IC_50_ values were computed based on a triplicate, eight-point titration. After 72 h of drug treatment, 10 μL of PrestoBlue Cell Viability Reagent was added to each well, and fluorescence signals were collected using a Wallac Vector 1420 multilabel counter (PerkinElmer, USA).

### Real-Time Quantitative PCR (qRT-PCR)

Total mRNAs were isolated from cells using RNeasy mini kit (Qiagen). Complementary DNAs were produced using SuperScript III Reverse Transcriptase (Thermo Fisher Scientific) and oligo dT (Invitrogen) and random hexamer (Fermentas) as primers. Quantitative PCR was carried out using Power SYBR Green master mix (Thermo Fisher Scientific) using primers for *cMYC* (5′-AAACACAAACTTGAACAGCTAC-3′ and 5′-ATTTGAGGCAGTTTACATTATGG-3′) and *GAPDH* (5′-GGAAGGTGAAGGTCGGAGTCA-3′ and 5′-GTCATTGATGGCAACAATATCCACT-3′).

### Pharmacokinetics

Procedures and use of animals were approved by the Institutional Animal Care and Use Committee (IACUC) of the National Health Research Institutes (NHRI, Zhunan, Miaoli, Taiwan). The facility where this research was conducted is accredited by the Association for Assessment and Accreditation of Laboratory Animal Care (AAALAC) International and adheres to principles stated in the Guide for the Care and Use of Laboratory Animals, National Research Council. Six-week-old male ICR mice obtained from BioLASCO, Taiwan, were used for the PK studies. A single 2 mg/kg intravenous and 10 mg/kg oral dose were separately administered to two groups of three male mice. Serial blood samples were collected from each mouse at times of 0.03, 0.08 (IV only), 0.25, 0.5, 1, 2, 4, 6, 8, and 24 h after dosing. Plasma was separated from blood by centrifugation and analyzed for compounds by LC–MS/MS. The chromatographic system consisted of an Agilent 1200 series LC system and an Agilent ZORBAX Eclipse XDB-C8 column (5 μm, 3.0 × 150 mm) interfaced to an MDS Sciex API3000 tandem mass spectrometer, equipped with an ESI in the positive scanning mode. A gradient HPLC method was employed for separation. PK parameters were calculated by the noncompartmental model using the Kinetica program.

### *In Vivo* Xenograft Tumor Growth Inhibition

Male athymic nu/nu nude mice (BioLASCO, Taiwan) 6 weeks old were housed in sterile cages maintained under 12 h light/dark cycles with controlled temperature and humidity. Mice were inoculated subcutaneously with 1 × 10^6^ NCI–H446 cells (ATCC@ HTB-171) resuspended in saline mixed with 50% Matrigel matrix (Corning, USA). The sizes of the xenografted tumors were measured by a digital caliper (GMC-190; Goldsun Electronics Co.) and calculated using the formula: tumor volume (mm^3^) = length × (width)2/2. Body weight and tumor size were measured at least twice a week. When the xenograft tumor reached ≥200 mm^3^ in size, mice were intravenous or orally administered with the vehicle (5% dimethylacetamide/95% PEG400) or the compounds on a 5-on-2-off dosing regimen for 2–4 weeks. Tumor growth was analyzed for a statistically significant difference using ANOVA, followed by the Student–Newman–Keuls test. *P* < 0.05 was considered a significant difference between groups.

### *In Vivo* Target Validation

The efficacy of a compound in reducing the MYC protein level and in inducing cell apoptosis was assessed using an NCI-H446 xenograft tumorigenicity mouse model. Six-week-old male athymic nu/nu nude mice (BioLASCO, Taiwan) were inoculated subcutaneously with 1 × 10^6^ NCI-H446 cells resuspended in saline mixed with 50% Matrigel matrix (Corning, USA). When the xenograft tumor reached a size ≥500 mm^3^, mice were orally administered with one dosage of the vehicle (5% dimethylacetamide/95% PEG400, 10 mL/kg) or **25** or MLN8237 at 100 mg/kg. Tumors were harvested after 2, 4, 8, and 24 h of drug treatment, respectively. Half of the tumor was cryopreserved and then subjected to western analysis; the other half was fixed in 10% formalin and then embedded in paraffin. For immunohistochemistry, tissue paraffin sections were deparaffinized using xylene and ethanol. After rehydration, antigen retrieval was achieved by placing the slides in 100 °C citrate buffer, pH 6.0 for 1 h. Endogenous peroxides were quenched by 0.5% H_2_O_2_ for 10 min. Slides were blocked with 3% BSA for 30 min and then incubated with a rabbit anticleaved Caspase-3 antibody (Cell signaling, 9661S) at room temperature for 1.5 h. After three washes with 1× PBS, a goat antirabbit HRP-polymer (Biocare Medical, USA) was added, followed by development of the color using 3,3′-diaminobenzidine substrate chromogen (Biocare Medical, USA). The nucleus was stained with hematoxylin.

## Supporting Information Available

A docking model of compound **1** (PDB) aligned with compound **B** (PDB) in complex with Aurora A (PDBID: 3UO6) (PDB)
Molecular formula strings and Aurora A enzymatic inhibition activities for **1**−**27** (CSV)
Western blot profile of cMYC and MYCN in cells treated with various Aurora kinase inhibitors, cell viability, cell cycle analysis and expression profile of the target proteins in cells treated with various Aurora kinase inhibitors, ^1^H, ^13^C NMR, and HPLC spectra of compounds **1–27**, and ^1^H spectra of compounds **29**, **30**, and **32–34** (PDF)

## Supplementary Material

jm0c01806_si_002.pdf

jm0c01806_si_003.pdb

jm0c01806_si_004.pdb

jm0c01806_si_005.pdb

## Abbreviations

ATP: adenosine triphosphate
AUC: area under the curve
BSA: bovine serum albumin
CASP3: caspase-3
cCASP3: cleaved caspase-3
cPARP-1: cleaved PARP-1
IHC: immunohistochemistry
IV: intravenous
PK: pharmacokinetic
PO: per os
SBDD: structure-based drug design
SCLC: small-cell lung cancer
TBST: Tris-buffered saline and 0.1% Tween-20
