# Supplementary material for: Discovery and Synthesis of a Pyrimidine-Based Aurora Kinase Inhibitor to Reduce Levels of MYC Oncoproteins
Source: J Med Chem. 2021 May 19;64(11):7312–30. doi: 10.1021/acs.jmedchem.0c01806 (PMC8279414; doi:10.1021/acs.jmedchem.0c01806)

Supporting Information (SI)

Discovery and Synthesis of a Pyrimidine-Based Aurora Kinase Inhibitor to Reduce Levels of MYC Oncoproteins

Ya-Hui Chi,[*‖] Teng-Kuang Yeh,[‖] Yi-Yu Ke, Wen-Hsing Lin, Chia-Hua Tsai, Wan-Ping Wang, Yen-Ting Chen, Yu-Chieh Su, Pei-Chen Wang, Yan-Fu Chen, Zhong-Wei Wu, Jen-Yu Yeh, Ming-Chun Hung, Mine-Hsine Wu, Jing-Ya Wang, Ching-Ping Chen, Jen-Shin Song, Chuan Shih, Chiung-Tong Chen, and Chun-Ping Chang[*]

Table of Contents

Figure S1. Western blot profile of cMYC and MYCN in cells treated with various Aurora kinase inhibitors……………………………………………………S3
Figure S2. Cell viability, cell cycle analysis and expression profile of the target proteins in cells treated with various Aurora kinase inhibitors………….……S4
Figure S3. NMR and HPLC spectra of compound 1 …………….………….S5
Figure S4. NMR and HPLC spectra of compound 2 …………….………….S7
Figure S5. NMR and HPLC spectra of compound 3 …………….………….S9
Figure S6. NMR and HPLC spectra of compound 4 ………….…..……S11
Figure S7. NMR and HPLC spectra of compound 5 ………….………..S13
Figure S8. NMR and HPLC spectra of compound 6 ………….………..S15
Figure S9. NMR and HPLC spectra of compound 7 ………….………..S17
Figure S10. NMR and HPLC spectra of compound 8 ………..………..S19
Figure S11. NMR and HPLC spectra of compound 9 ………………….S21
Figure S12. NMR and HPLC spectra of compound 10 …………...…S23
Figure S13. NMR and HPLC spectra of compound 11 …………...…S25
Figure S14. NMR and HPLC spectra of compound 12 …………...…S27
Figure S15. NMR and HPLC spectra of compound 13 …………...…S29
Figure S16. NMR and HPLC spectra of compound 14 …………...…S31
Figure S17. NMR and HPLC spectra of compound 15 …………...…S33
Figure S18. NMR and HPLC spectra of compound 16 …………...…S35
Figure S19. NMR and HPLC spectra of compound 17 …………...…S37
Figure S20. NMR and HPLC spectra of compound 18 …………...…S39
Figure S21. NMR and HPLC spectra of compound 19 …………...…S41
Figure S22. NMR and HPLC spectra of compound 20 …………...…S43
Figure S23. NMR and HPLC spectra of compound 21 …………...…S45
Figure S24. NMR and HPLC spectra of compound 22 …………...…S47
Figure S25. NMR and HPLC spectra of compound 23 …………...…S49
Figure S26. NMR and HPLC spectra of compound 24 …………...…S51
Figure S27. NMR and HPLC spectra of compound 25 …………...…S53
Figure S28. NMR and HPLC spectra of compound 26 …………...…S55
Figure S29. NMR and HPLC spectra of compound 27 …………...…S57
Figure S30. ^{1}H NMR spectrum of compound 29 ………….………...…S59
Figure S31. ^{1}H NMR spectrum of compound 30 ………….………...…S60
Figure S32. ^{1}H NMR spectrum of compound 32 ………….………...…S61
Figure S33. ^{1}H NMR spectrum of compound 33 ………….………...…S62
Figure S34. ^{1}H NMR spectrum of compound 34 ………….………...…S63

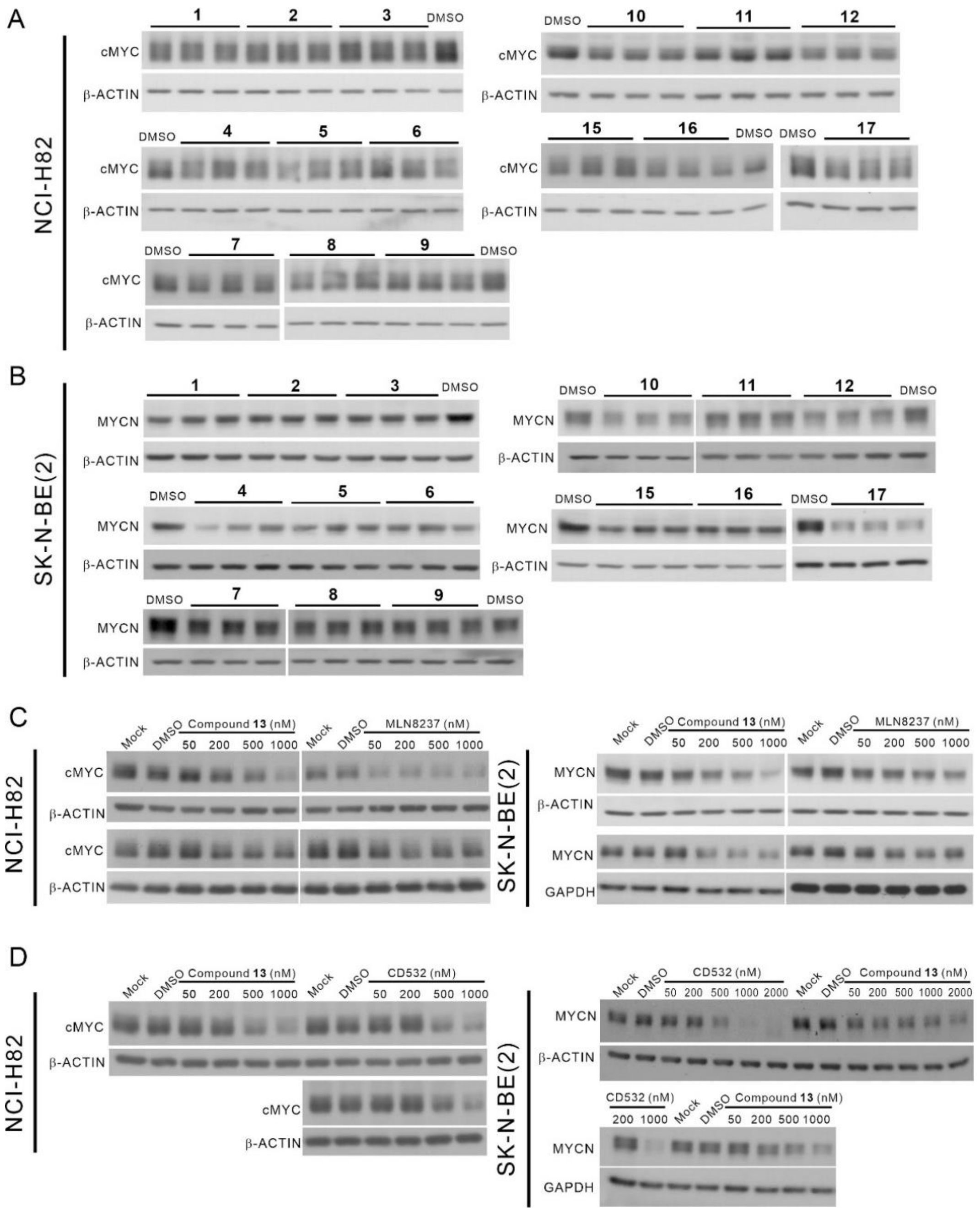

Figure S1. (A-C) Western blot analysis for the expression levels of cMYC and MYCN in NCI-H82 and SK-N-BE(2) cells respectively treated with 1.0 μM of the indicated compounds, or various concentrations of **13**/MLN8237 for 24 h. The cMYC/MYCN levels undertaken in triplicate were normalized to β-ACTIN or GAPDH, and are presented in Table 1. (D) Western blot analysis for the comparison of cMYC/MYCN protein expression profiles in NCI-H82 and SK-N-BE(2) cells respectively treated with various concentrations of **13** and CD532 for 24 h.

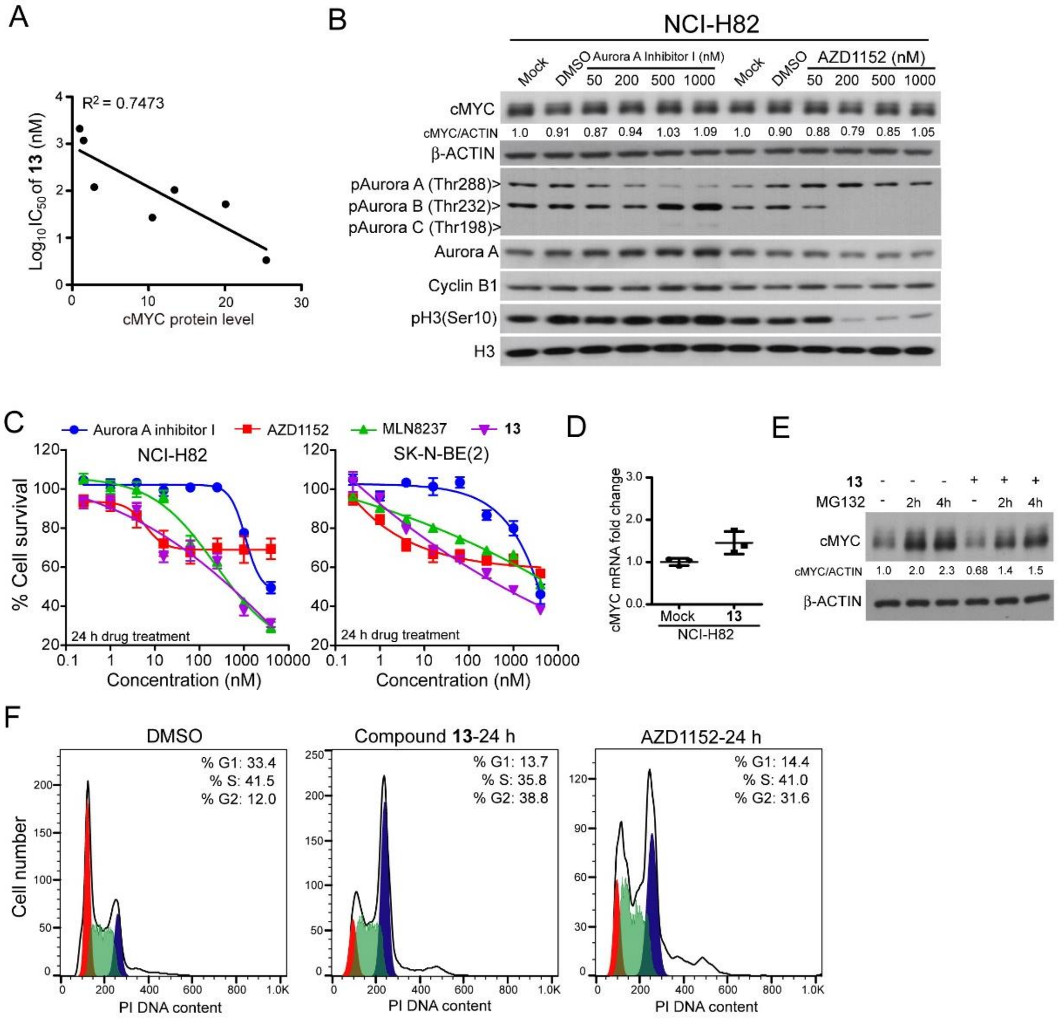

Figure S2. (A) Correlation between half growth inhibition concentrations (IC_{50}, log_{10} scale) of **13** and relative expression levels of cMYC in the seven SCLC cell lines (i.e. NCI-H82, NCI-H446, NCI-H211, NCI-H524, NCI-H2171, NCI-H146 and NCI-H841) in Figure 2E. The computed coefficient of determination R^2 is 0.7473. (B) Western blot analysis for the expression levels of cMYC, phosphorylated Aurora kinase A/B/C and cell cycle markers Cyclin B1 and pH3(Ser10), in NCI-H82 cells respectively treated with Aurora A inhibitor I and AZD1152 at the indicated concentrations for 24 h. Immunoblotting of β-ACTIN was used as a loading control. Relative expression of cMYC normalized to β-ACTIN was denoted. (C) Percent survival of NCI-H82 and SK-N-BE(2) cells treated with Aurora A inhibitor I, AZD1152, **13**, or MLN8237 for 24 h. (D) Relative mRNA level of *cMYC* in NCI-H82 mock- or **13**- treated for 24 h. (E) Western blotting analysis for the cMYC protein expression in NCI-H82 cells with/without **13** treatment for 5 h, and in the presence/absence of 1.0 μg/mL MG132 for 2 or 4 h. Immunoblotting of β-ACTIN was used as a loading control. A slight reduction in cMYC level was observed in NCI-H82 cells treated with **13** for 5 h. (F) Cell cycle analysis of NCI-H82 treated with 0.1% DMSO, 1.0 μM of **13** or 1.0 μM of AZD1152 for 24 h. Cells were fixed in 70% ethanol, stained with propidium iodide (PI)/0.1 % Triton X-100 staining solution, and were analyzed using a FACSCalibur™ (BD Biosciences). Data were processed by FlowJo™ software. Percent of cells in G1, S or G2/M (i.e. G2 in FlowJo™ software) phases are denoted on the figure.

Figure S3. NMR and HPLC spectra of compound 1.

(*S*)-(3-chloro-2-fluorophenyl)(3-((4-(4-ethylpiperazin-1-yl)-6-((5-methyl-1H-pyrazol-3-yl)amino)pyrimidin-2-yl)amino)pyrrolidin-1-yl)methanone (1)

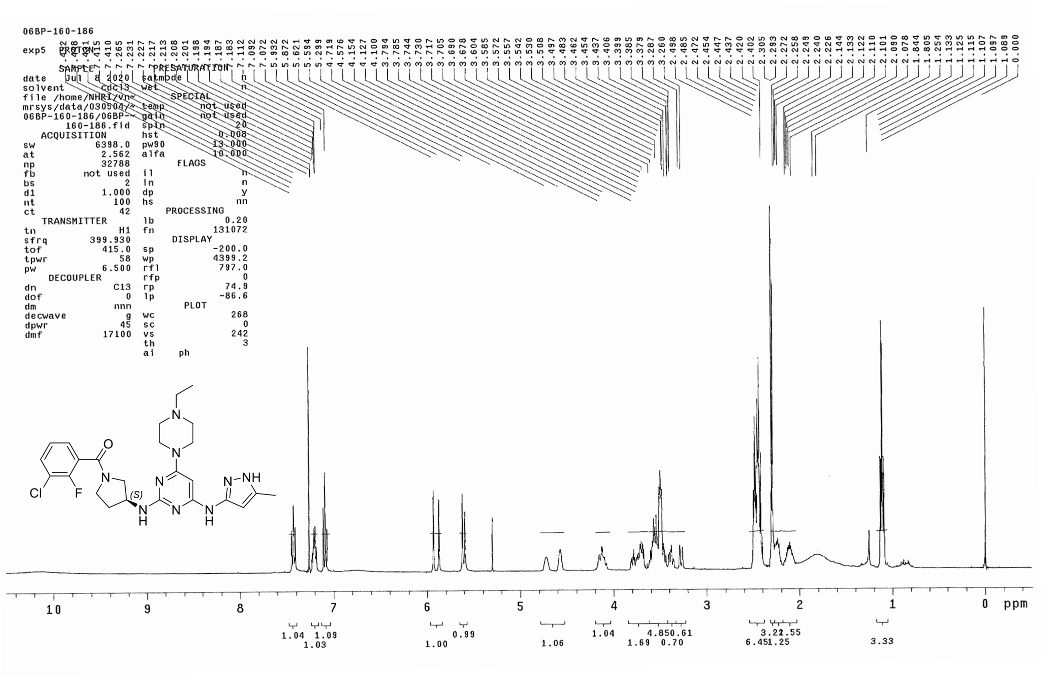

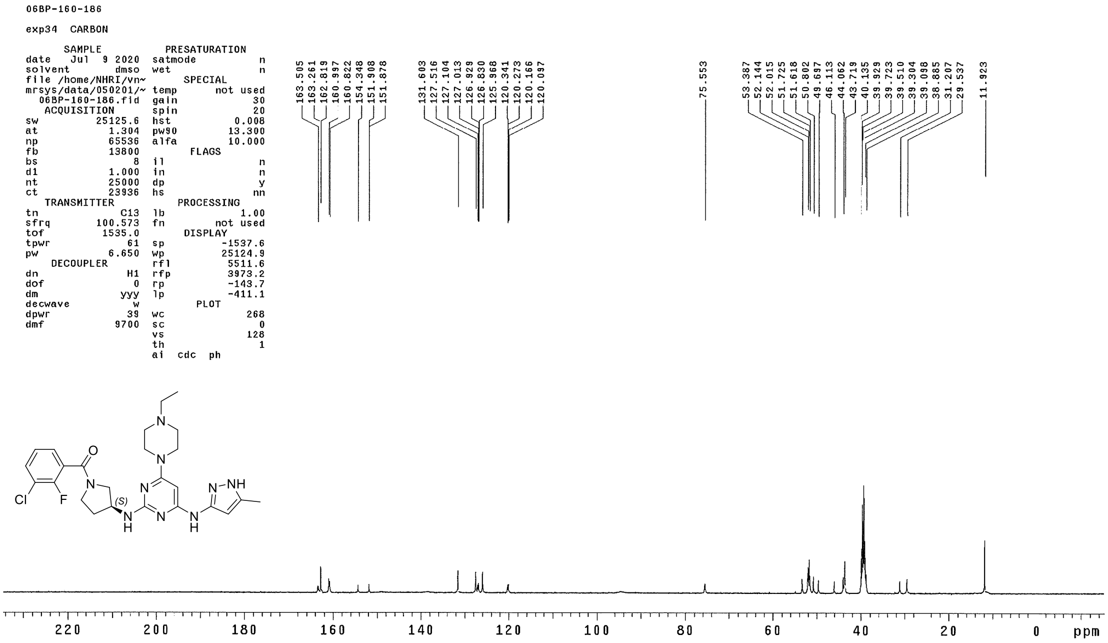

Sample Name: 06BP-160-186-50 Vial Number: 102
Injection from this vial: 1 of 1 Volume: 20.0 ul
Sample Description:

Chrom Type: Fixed WL Chromatogram, 254 nm

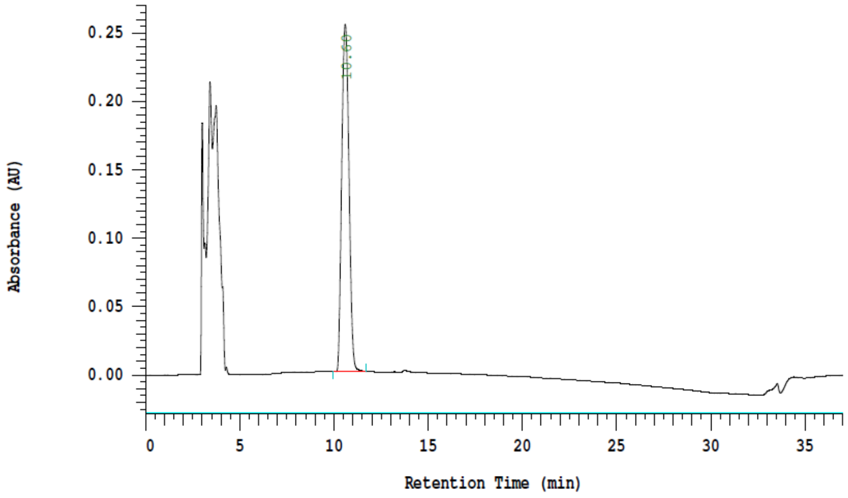

Processing Method: Purity_37min
Column Type: Column Method Developer: Bob
Method Description:

Peak Quantitation: AREA
Calculation Method: AREA%

No.	RT	Area	Height	Conc 1
1	10.60	3357313	126883	100.000
		3357313	126883	100.000

Peak rejection level: 0

Figure S4. NMR and HPLC spectra of compound 2.

(*S*)-(3-chloro-2-fluorophenyl)(3-((4-((5-methyl-1H-pyrazol-3-yl)amino)-6-(4-methylpiperazin-1-yl)pyrimidin-2-yl)amino)pyrrolidin-1-yl)methanone (2)

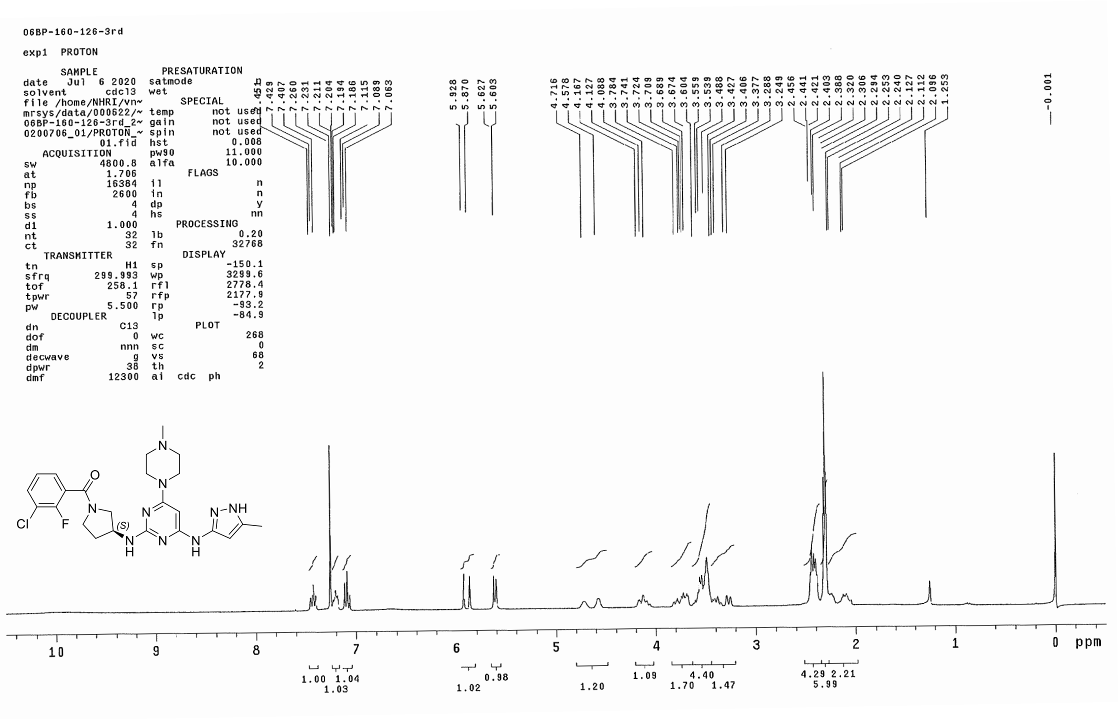

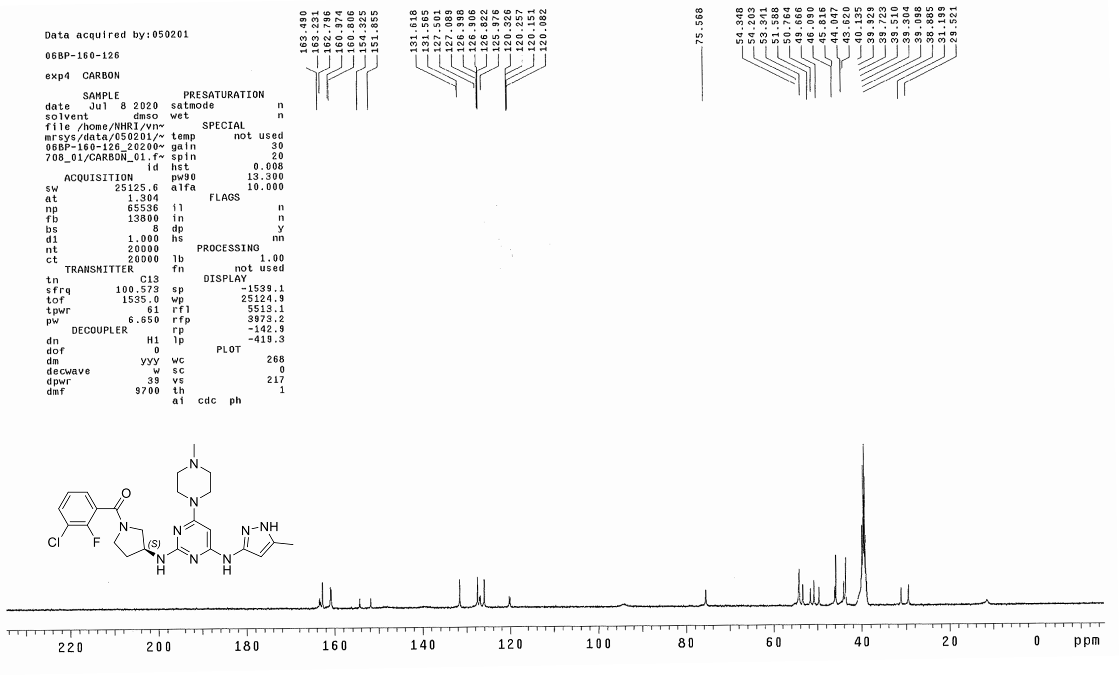

Sample Name: 06BP-160-126-50 Vial Number: 138
Injection from this vial: 1 of 1 Volume: 20.0 ul
Sample Description:

Chrom Type: Fixed WL Chromatogram, 254 nm

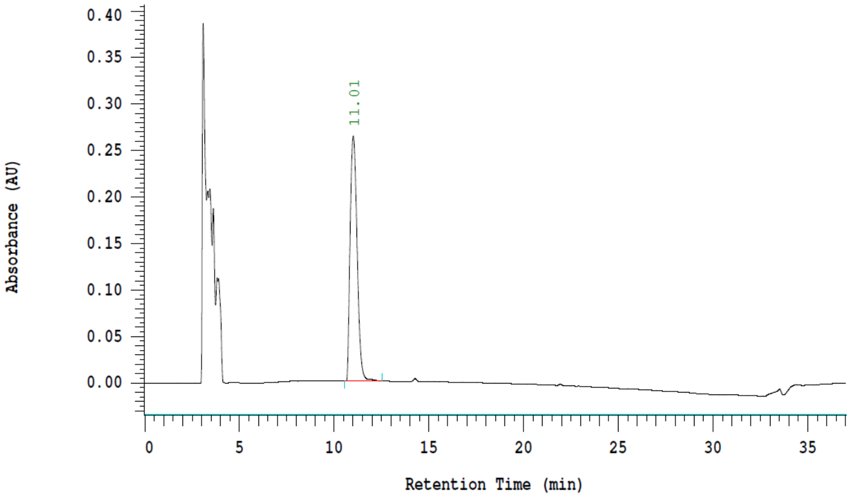

Processing Method: Purity_37min
Column Type: Column Method Developer: Bob
Method Description:

Peak Quantitation: AREA
Calculation Method: AREA%

No.	RT	Area	Height	Conc 1
1	11.01	3333011	131560	100.000
		3333011	131560	100.000

Peak rejection level: 0

Figure S5. NMR and HPLC spectra of compound 3.

(*S*)-(3-chloro-2-fluorophenyl)(3-((4-(3-(dimethylamino)azetidin-1-yl)-6-((5-methyl-1H-pyrazol-3-yl)amino)pyrimidin-2-yl)amino)pyrrolidin-1-yl)methanone (3)

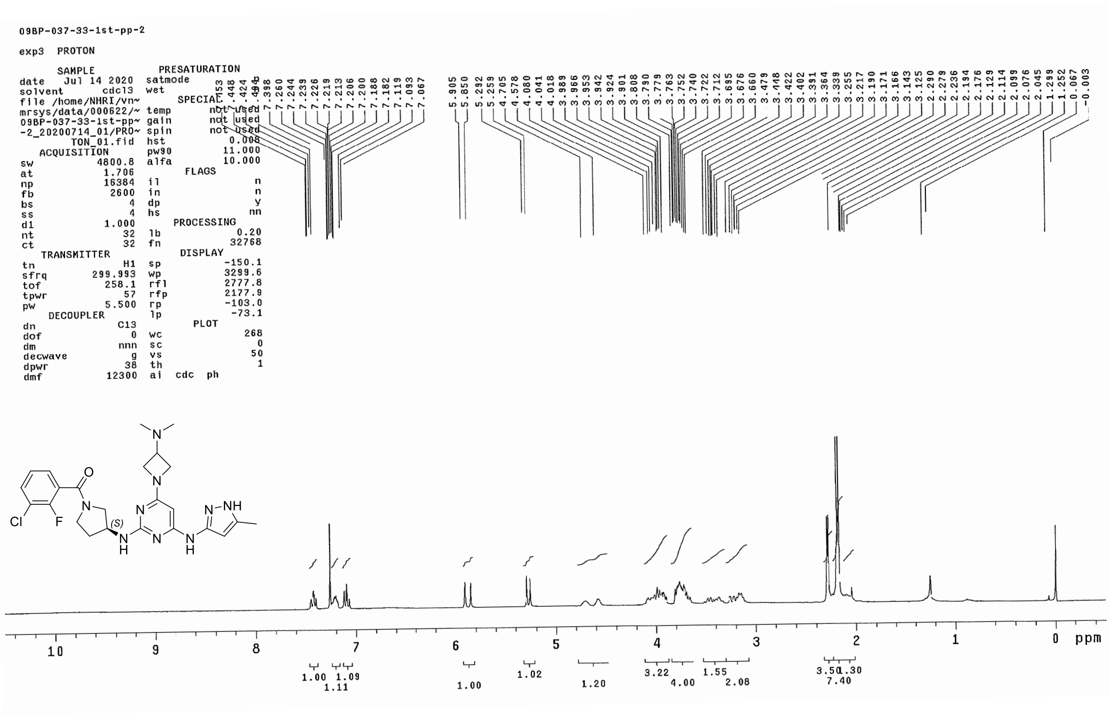

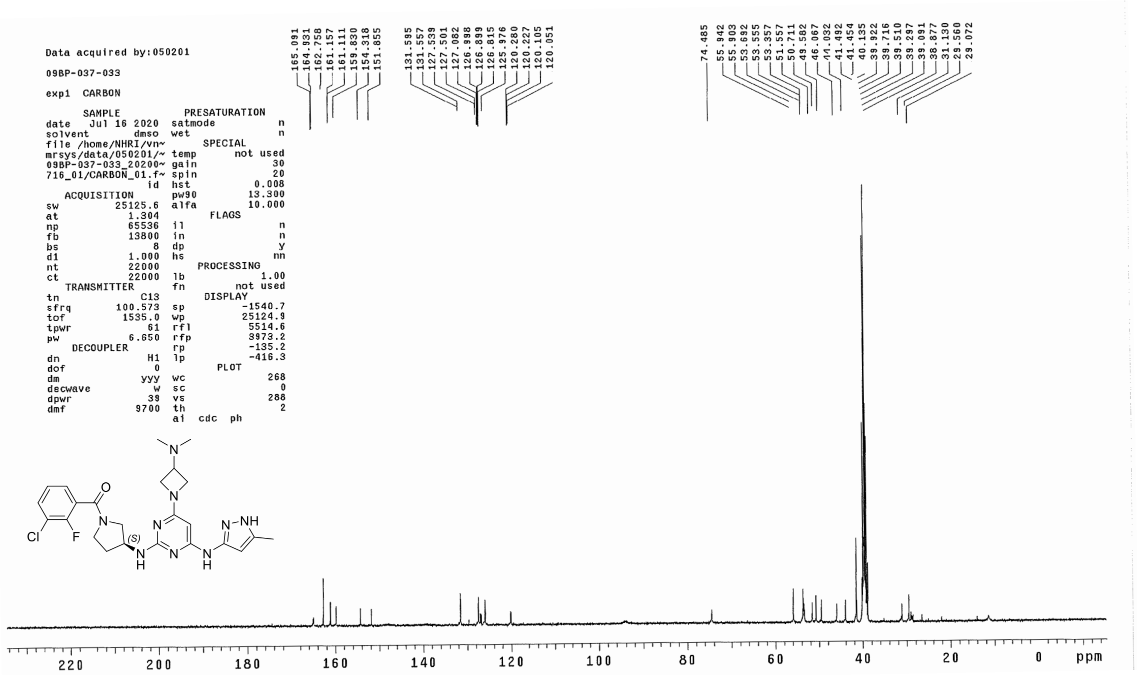

Sample Name: 09BP-037-33-1st-PP-1-50   Vial Number: 128
Injection from this vial: 1 of 1   Volume: 20.0 ul
Sample Description:

Chrom Type: Fixed WL Chromatogram, 254 nm

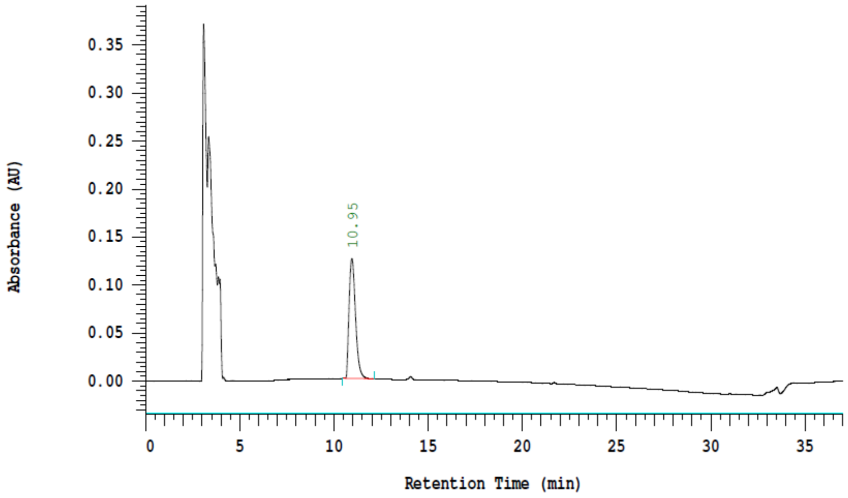

Processing Method: Purity_37min
Column Type: Column   Method Developer: Bob
Method Description:

Peak Quantitation: AREA
Calculation Method: AREA%

No.	RT	Area	Height	Conc 1
1	10.95	1468768	62258	100.000
		1468768	62258	100.000

Peak rejection level: 0

Figure S6. NMR and HPLC spectra of compound 4.

(3-chloro-2-fluorophenyl)((S)-3-((4-((S)-3-(dimethylamino)pyrrolidin-1-yl)-6-((5-methyl-1H-pyrazol-3-yl)amino)pyrimidin-2-yl)amino)pyrrolidin-1-yl)methanone (4)

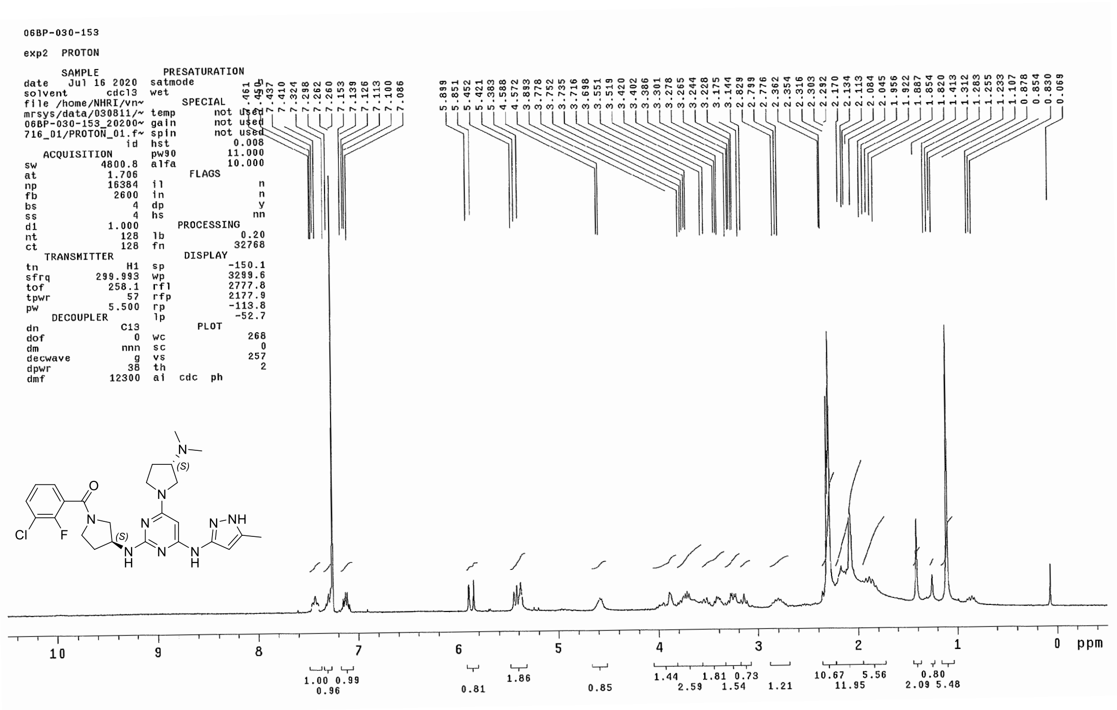

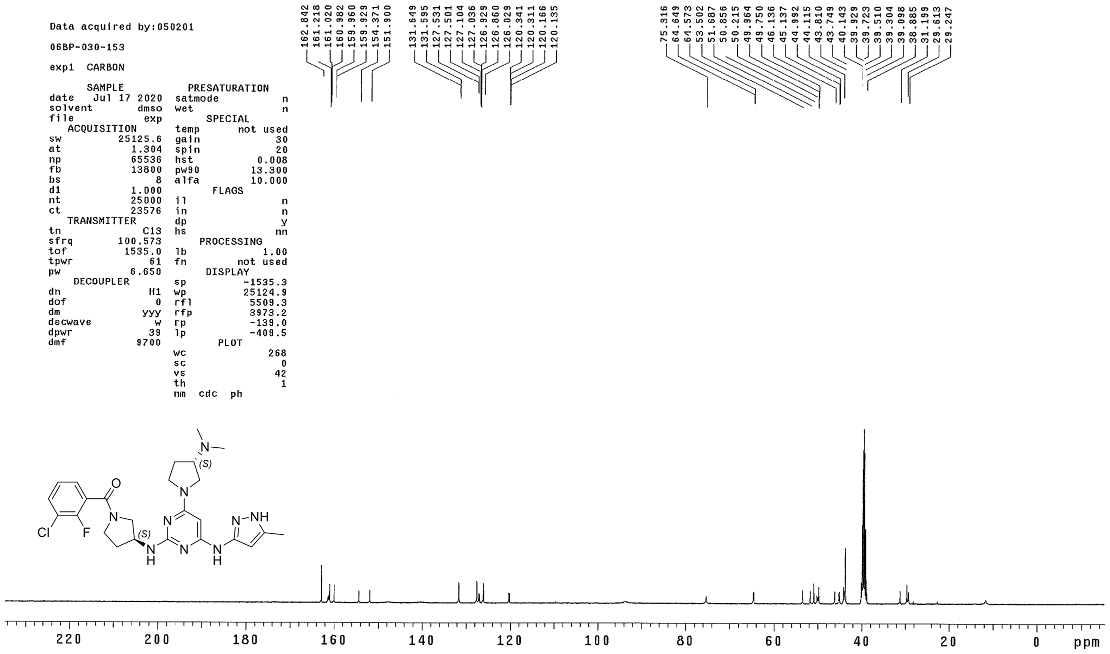

Sample Name: 06BP-030-153-50 Vial Number: 142
Injection from this vial: 1 of 1 Volume: 20.0 ul
Sample Description:

Chrom Type: Fixed WL Chromatogram, 254 nm

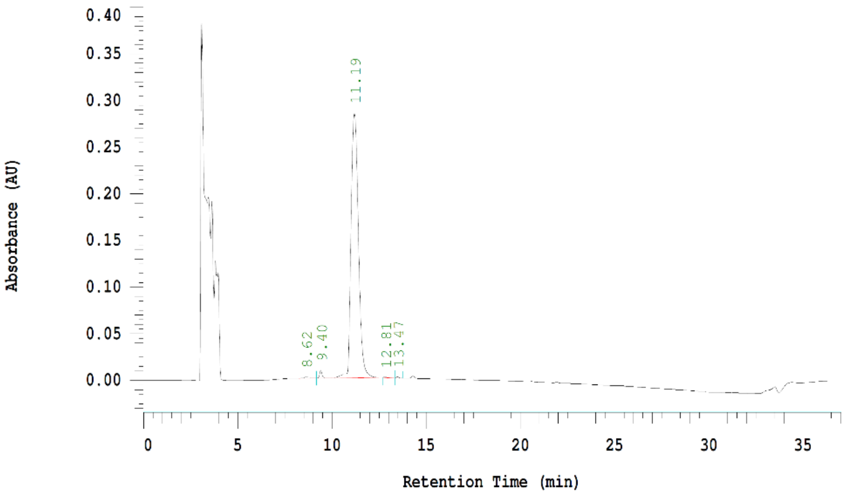

Processing Method: Purity_37min
Column Type: Column Method Developer: Bob
Method Description:

Peak Quantitation: AREA
Calculation Method: AREA%

No.	RT	Area	Height	Conc 1
1	8.62	14326	556	0.367
2	9.40	37144	4226	0.952
3	11.19	3842063	141755	98.474
4	12.81	1836	215	0.047
5	13.47	6239	743	0.160
		3901608	147495	100.000

Peak rejection level: 0

Figure S7. NMR and HPLC spectra of compound 5.

(*S*)-(3-chloro-2-fluorophenyl)(3-((4-((2-(dimethylamino)ethyl)(methyl)amino)-6-((5-methyl-1H-pyrazol-3-yl)amino)pyrimidin-2-yl)amino)pyrrolidin-1-yl)methanone (5)

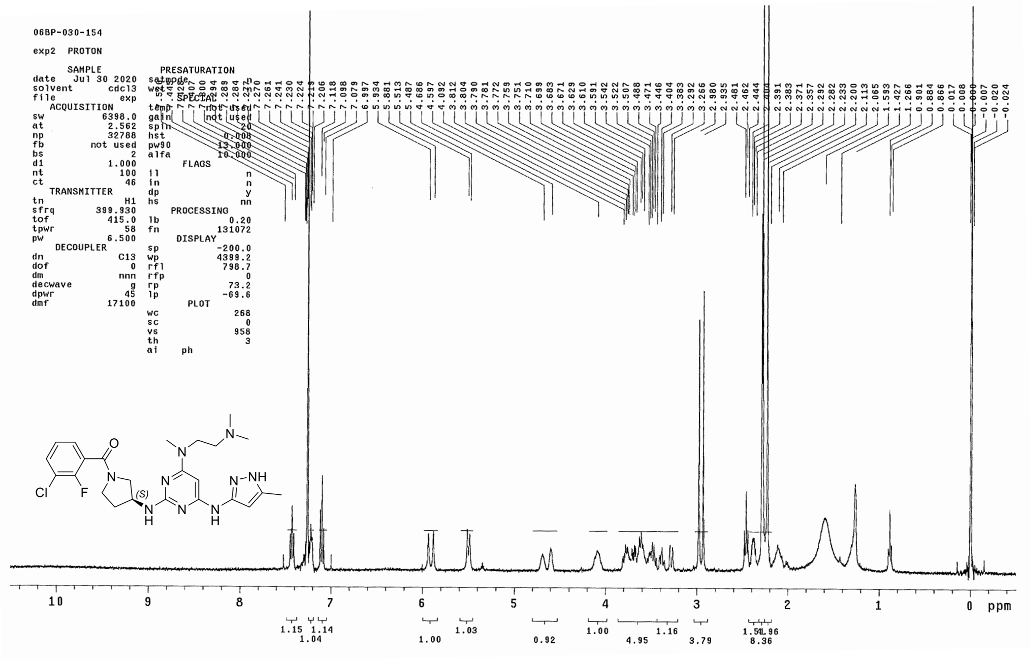

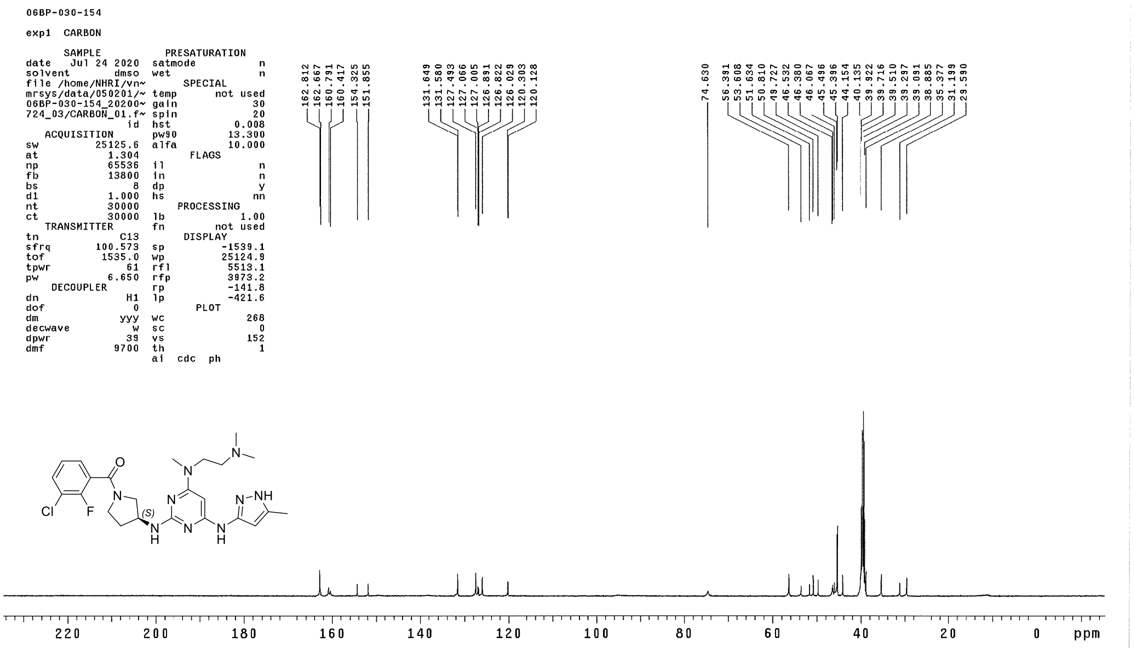

Sample Name: 06BP-030-154-50 Vial Number: 150
Injection from this vial: 1 of 1 Volume: 20.0 ul
Sample Description:

Chrom Type: Fixed WL Chromatogram, 254 nm

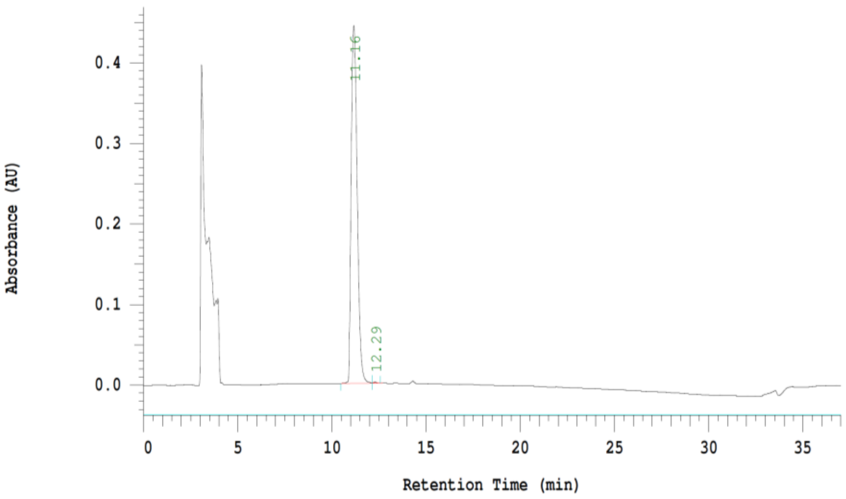

Processing Method: Purity_37min
Column Type: Column Method Developer: Bob
Method Description:

Peak Quantitation: AREA
Calculation Method: AREA%

No.	RT	Area	Height	Conc 1
1	11.16	4852782	221908	99.906
2	12.29	4585	464	0.094
		4857367	222372	100.000

Peak rejection level: 0

Figure S8. NMR and HPLC spectra of compound 6.

(*S*)-(3-((4-(4-ethylpiperazin-1-yl)-6-((5-methyl-1H-pyrazol-3-yl)amino)pyrimidin-2-yl)amino)pyrrolidin-1-yl)(phenyl)methanone (6)

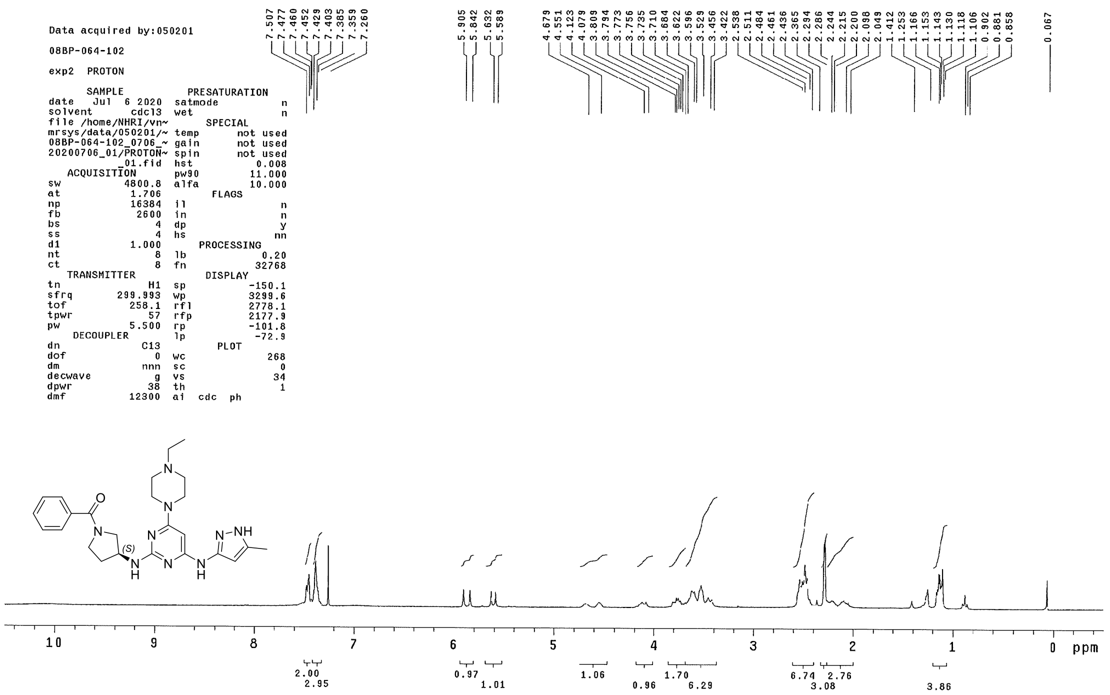

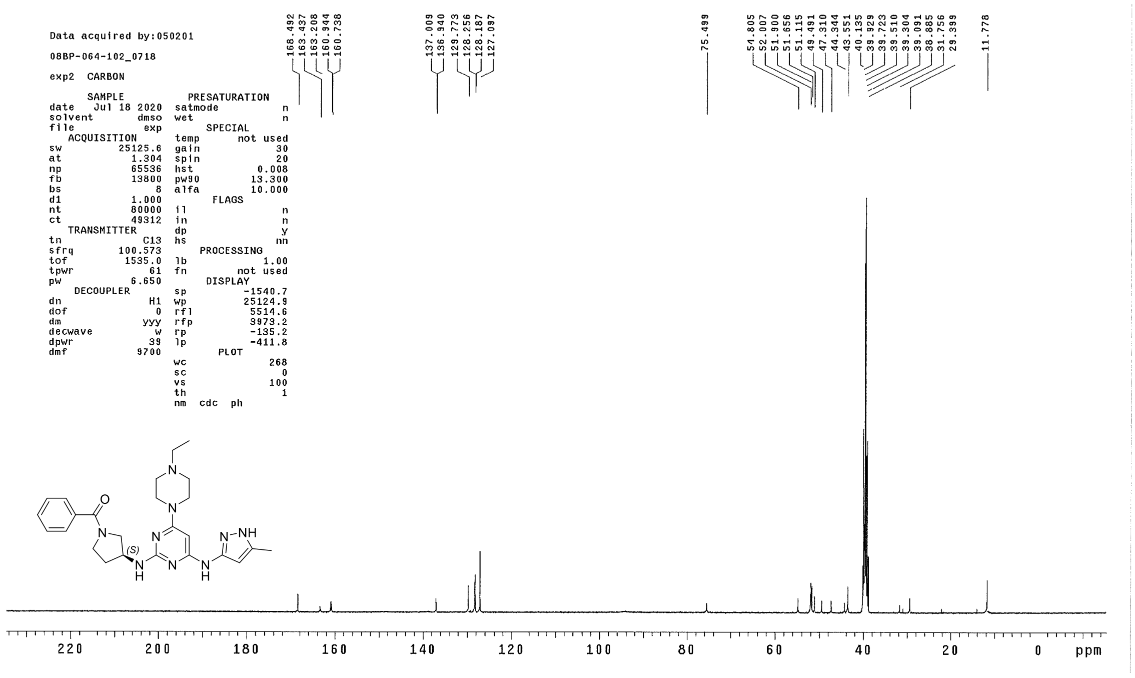

Sample Name: 08BP-064-102-50 Vial Number: 4
Injection from this vial: 1 of 1 Volume: 20.0 ul
Sample Description:

Chrom Type: Fixed WL Chromatogram, 254 nm

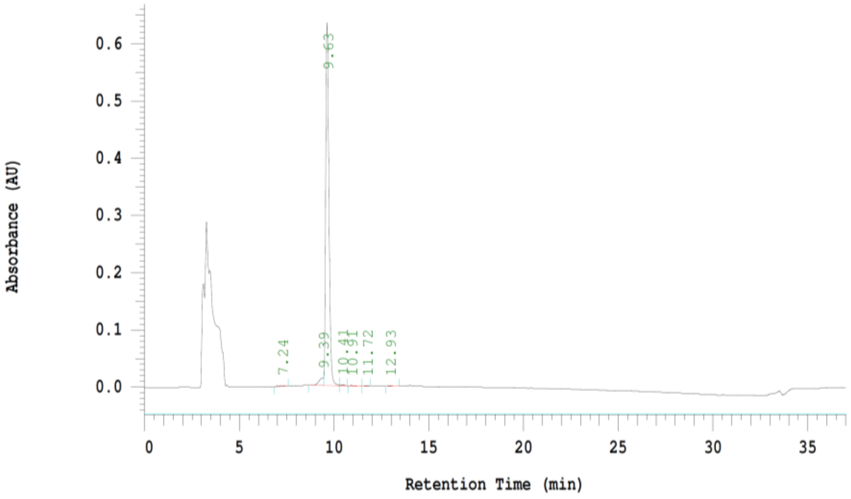

Processing Method: Purity_37min
Column Type: Column Method Developer: Bob
Method Description:

Peak Quantitation: AREA
Calculation Method: AREA%

No.	RT	Area	Height	Conc 1
1	7.24	10782	527	0.279
2	9.39	106876	6567	2.765
3	9.63	3726693	316638	96.407
4	10.41	7022	568	0.182
5	10.91	5611	421	0.145
6	11.72	3650	306	0.094
7	12.93	4944	348	0.128
		3865578	325375	100.000

Peak rejection level: 0

Figure S9. NMR and HPLC spectra of compound 7.

(*S*)-(4-chlorophenyl)(3-((4-(4-ethylpiperazin-1-yl)-6-((5-methyl-1H-pyrazol-3-yl)amino)pyrimidin-2-yl)amino)pyrrolidin-1-yl)methanone (7)

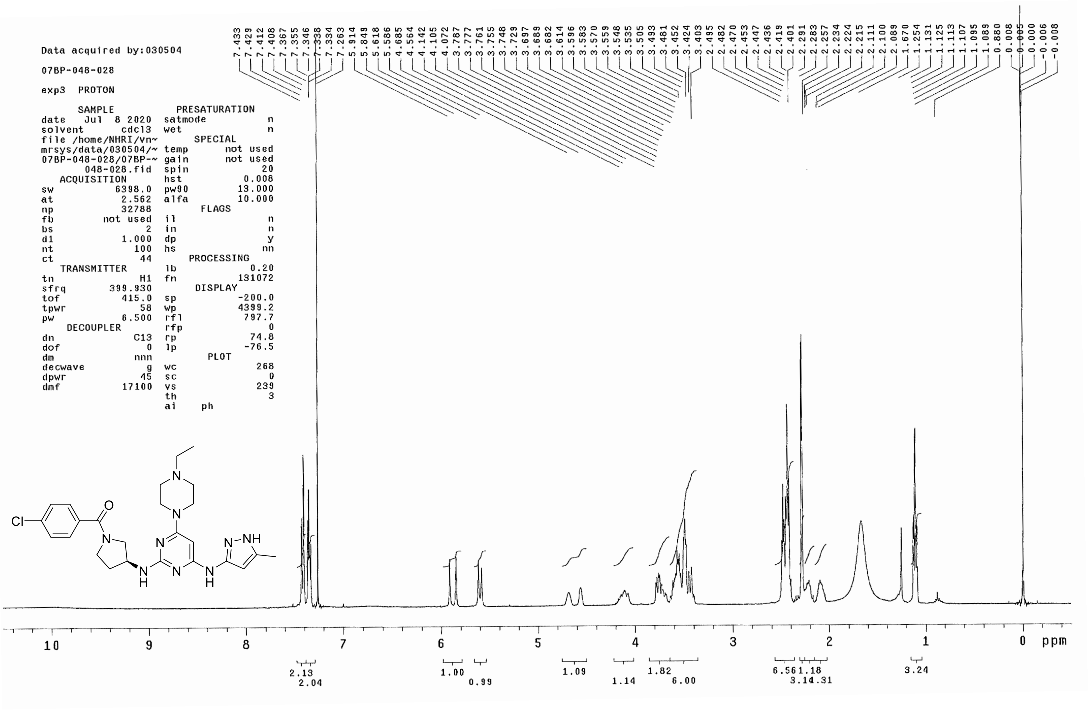

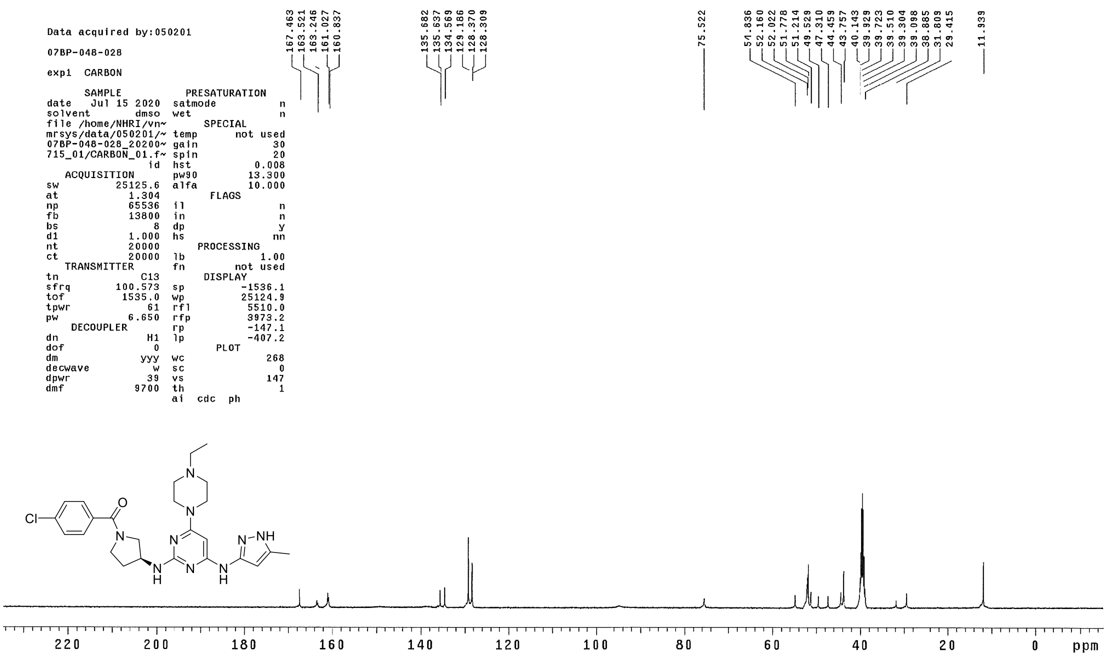

Sample Name: 07BP-048-028-50 Vial Number: 108
Injection from this vial: 1 of 1 Volume: 20.0 ul
Sample Description:

Chrom Type: Fixed WL Chromatogram, 254 nm

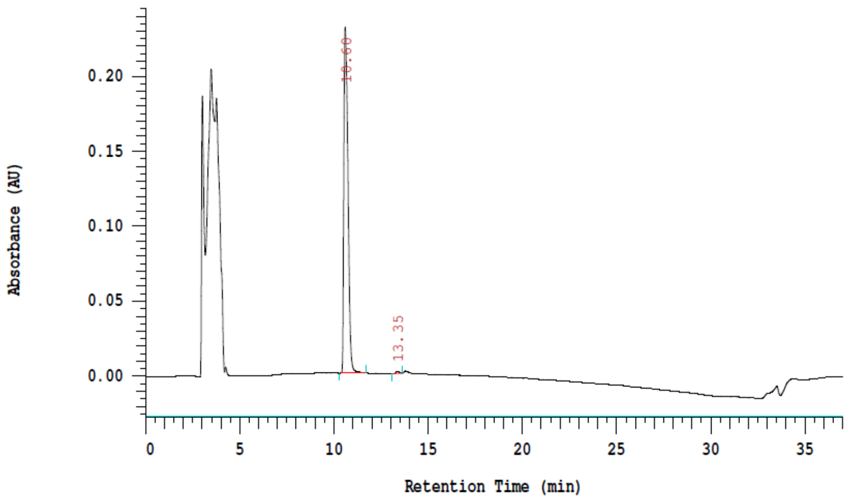

Processing Method: Purity_37min
Column Type: Column Method Developer: Bob
Method Description:

Peak Quantitation: AREA
Calculation Method: AREA%

No.	RT	Area	Height	Conc 1
1	10.60	1721077	115221	99.629
2	13.35	6406	514	0.371
		1727483	115735	100.000

Peak rejection level: 0

Figure S10. NMR and HPLC spectra of compound 8.

(*S*)-(2,4-dichlorophenyl)(3-((4-(4-ethylpiperazin-1-yl)-6-((5-methyl-1H-pyrazol-3-yl)amino)pyrimidin-2-yl)amino)pyrrolidin-1-yl)methanone (8)

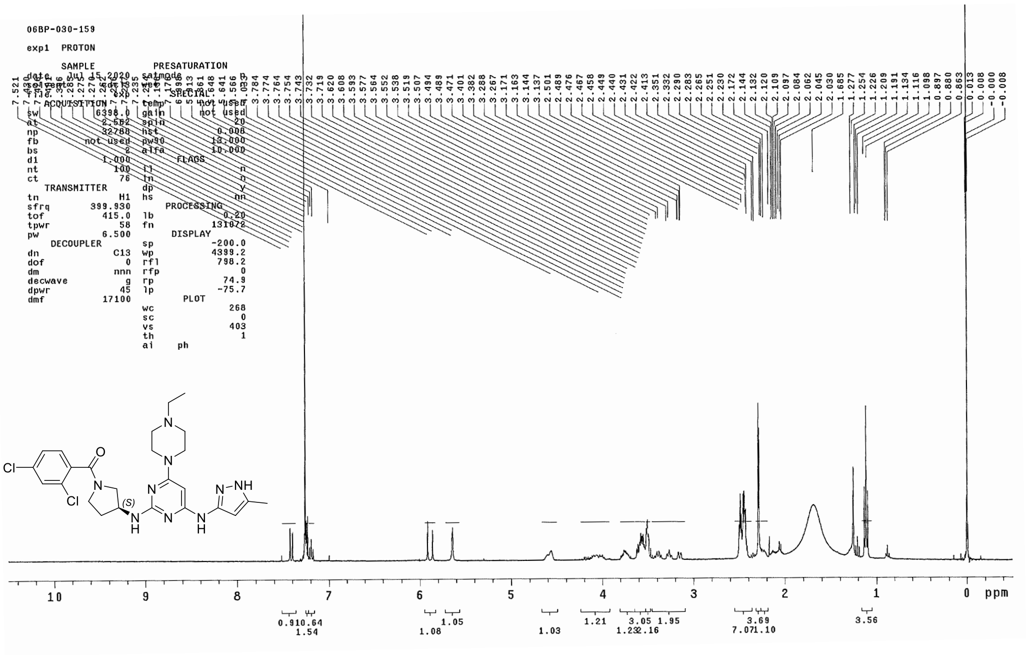

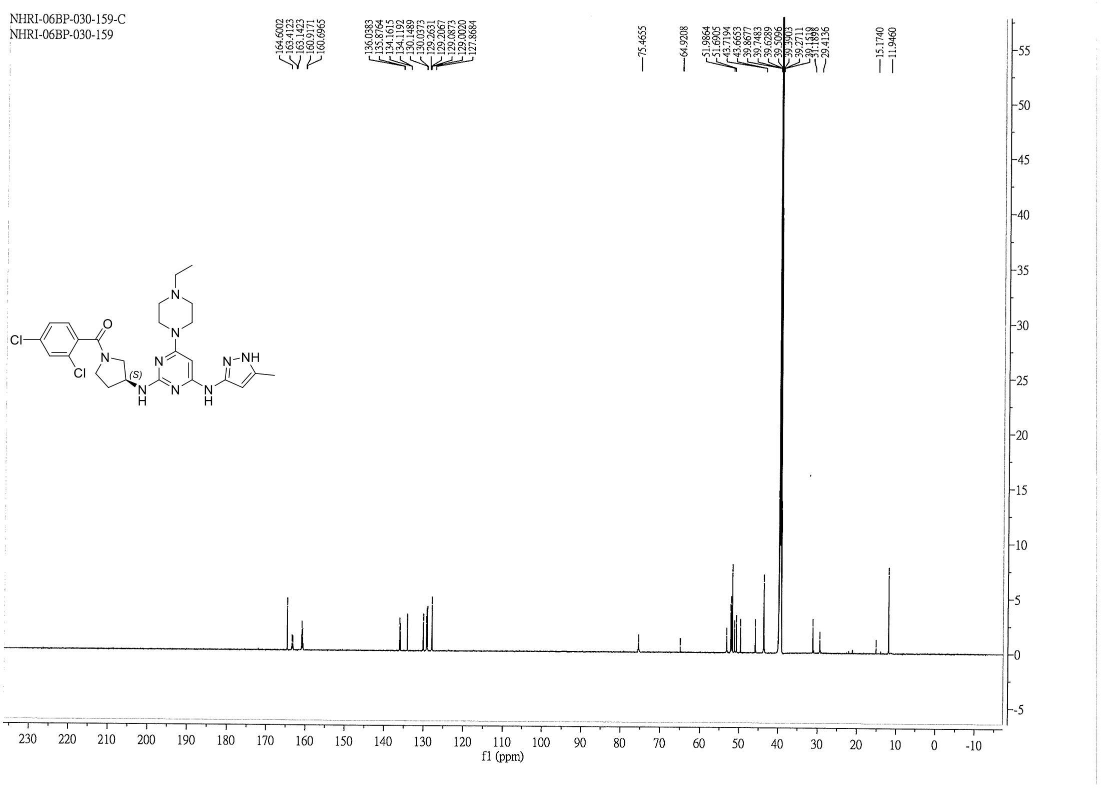

Sample Name: 06BP-030-159-50   Vial Number: 110
Injection from this vial: 1 of 1   Volume: 20.0 ul
Sample Description:

Chrom Type: Fixed WL Chromatogram, 254 nm

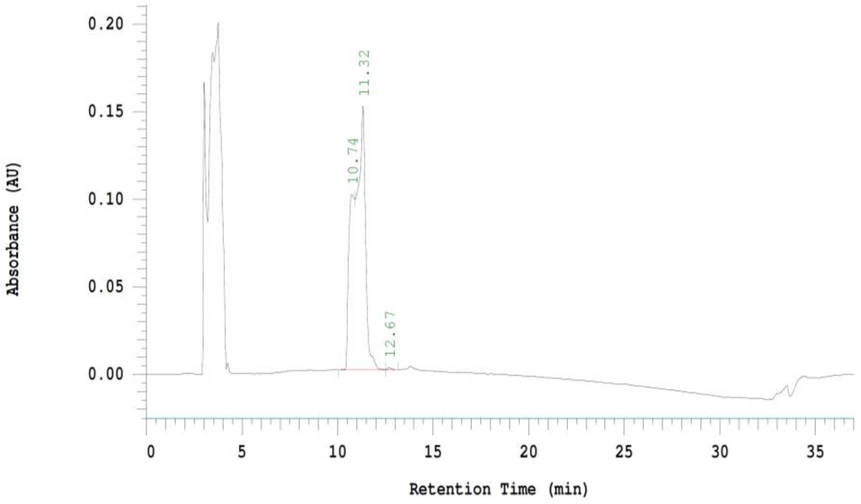

Processing Method: Purity_37min
Column Type: Column   Method Developer: Bob
Method Description:

Peak Quantitation: AREA
Calculation Method: AREA%

No.	RT	Area	Height	Conc 1
1	10.74	1043036	50100	32.101
2	11.32	2197890	75149	67.644
3	12.67	8257	590	0.254
		3249183	125839	100.000

Peak rejection level: 0

Figure S11. NMR and HPLC spectra of compound 9.

(*S*)-(4-chloro-2-methylphenyl)(3-((4-(4-ethylpiperazin-1-yl)-6-((5-methyl-1H-pyrazol-3-yl)amino)pyrimidin-2-yl)amino)pyrrolidin-1-yl)methanone (9)

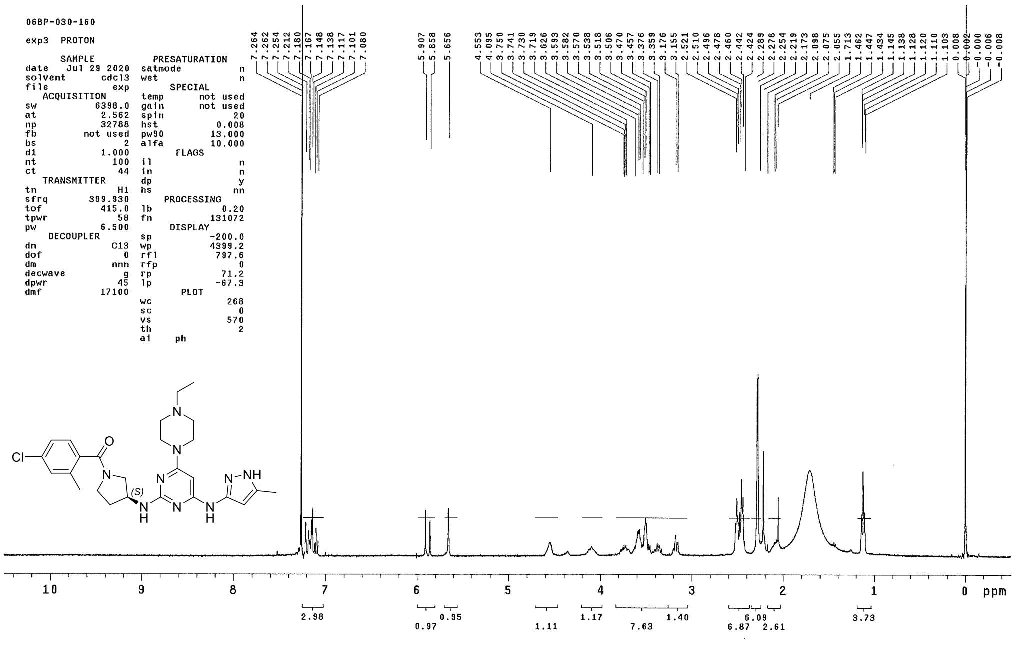

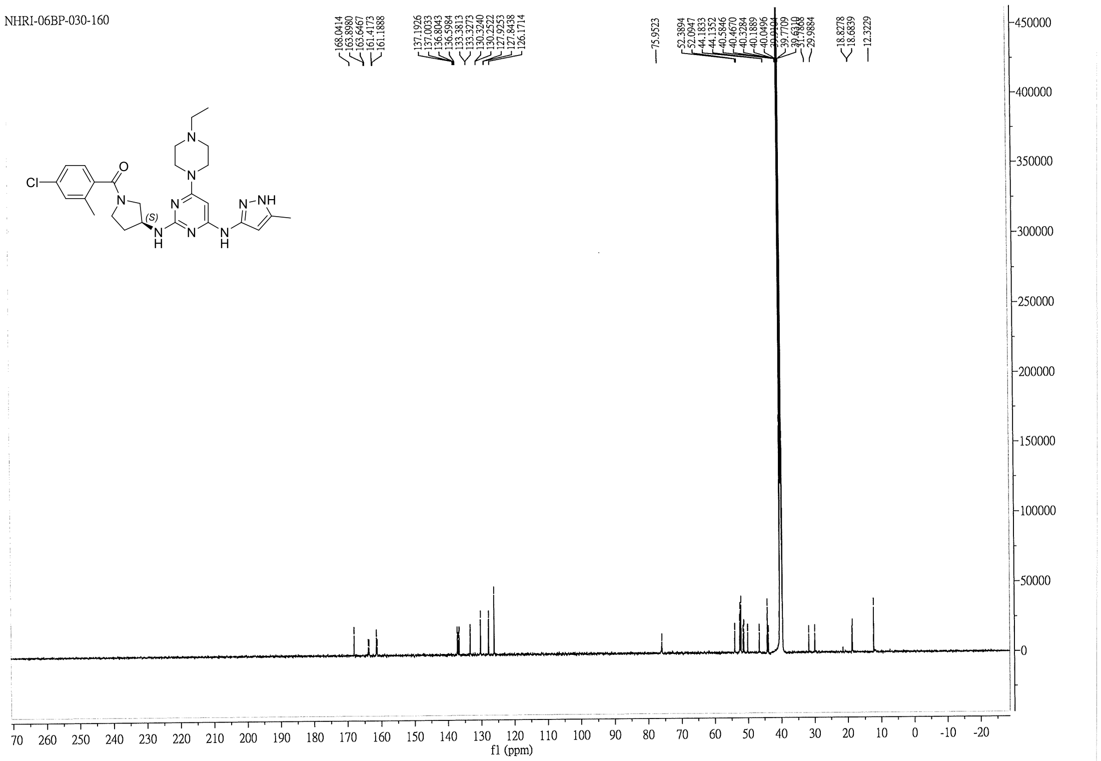

Sample Name: 06BP-030-160-50 Vial Number: 64
Injection from this vial: 1 of 1 Volume: 20.0 ul
Sample Description:

Chrom Type: Fixed WL Chromatogram, 254 nm

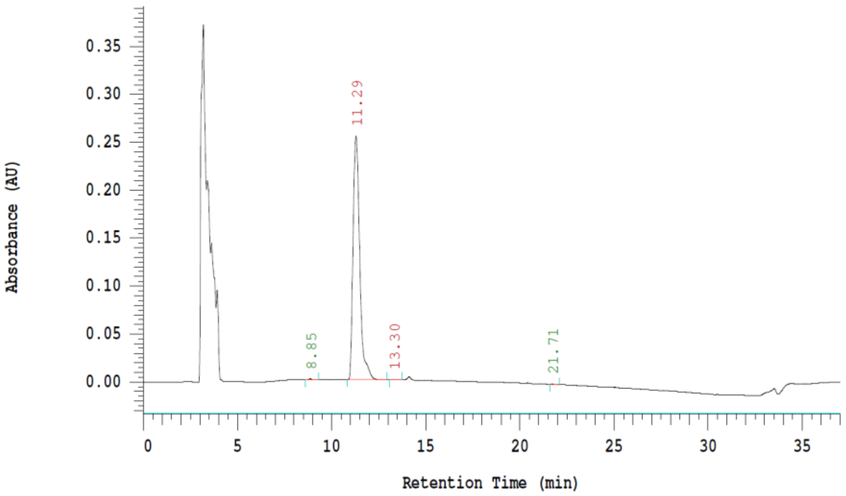

Processing Method: Purity_37min
Column Type: Column Method Developer: Bob
Method Description:

Peak Quantitation: AREA
Calculation Method: AREA%

No.	RT	Area	Height	Conc 1
1	8.85	6463	640	0.208
2	11.29	3096628	127017	99.646
3	13.30	2501	147	0.080
4	21.71	2048	202	0.066
		3107640	128006	100.000

Peak rejection level: 0

Figure S12. NMR and HPLC spectra of compound 10.

(*S*)-(4-chloro-3-fluorophenyl)(3-((4-(4-ethylpiperazin-1-yl)-6-((5-methyl-1H-pyrazol-3-yl)amino)pyrimidin-2-yl)amino)pyrrolidin-1-yl)methanone (10)

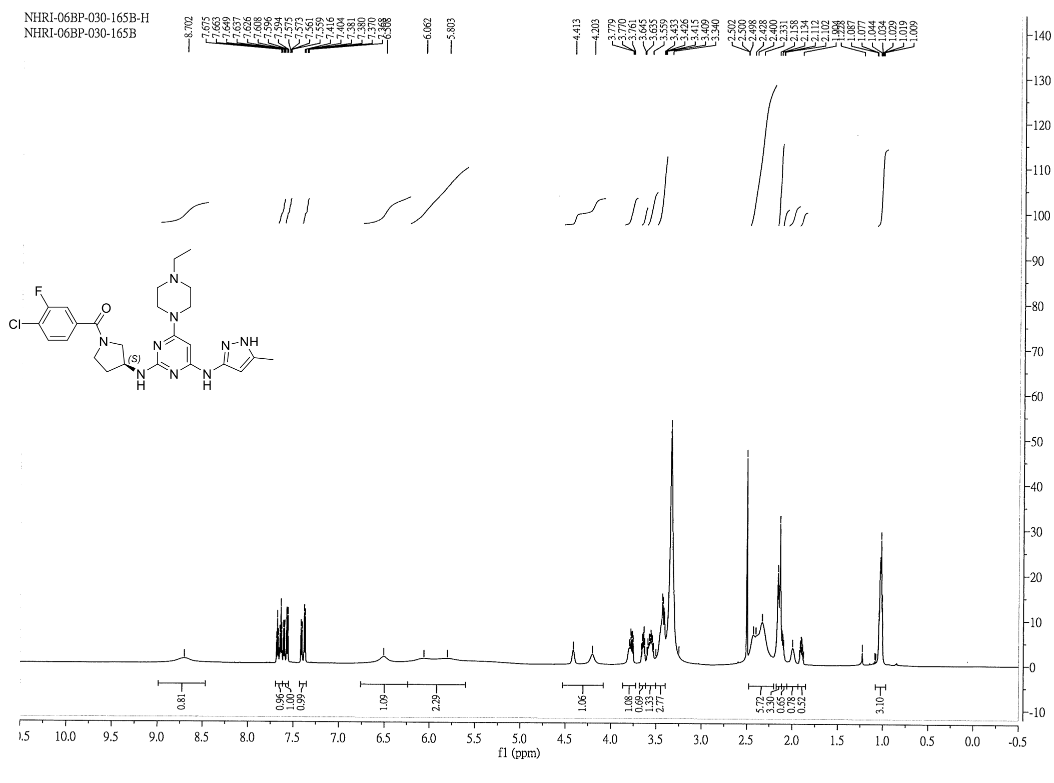

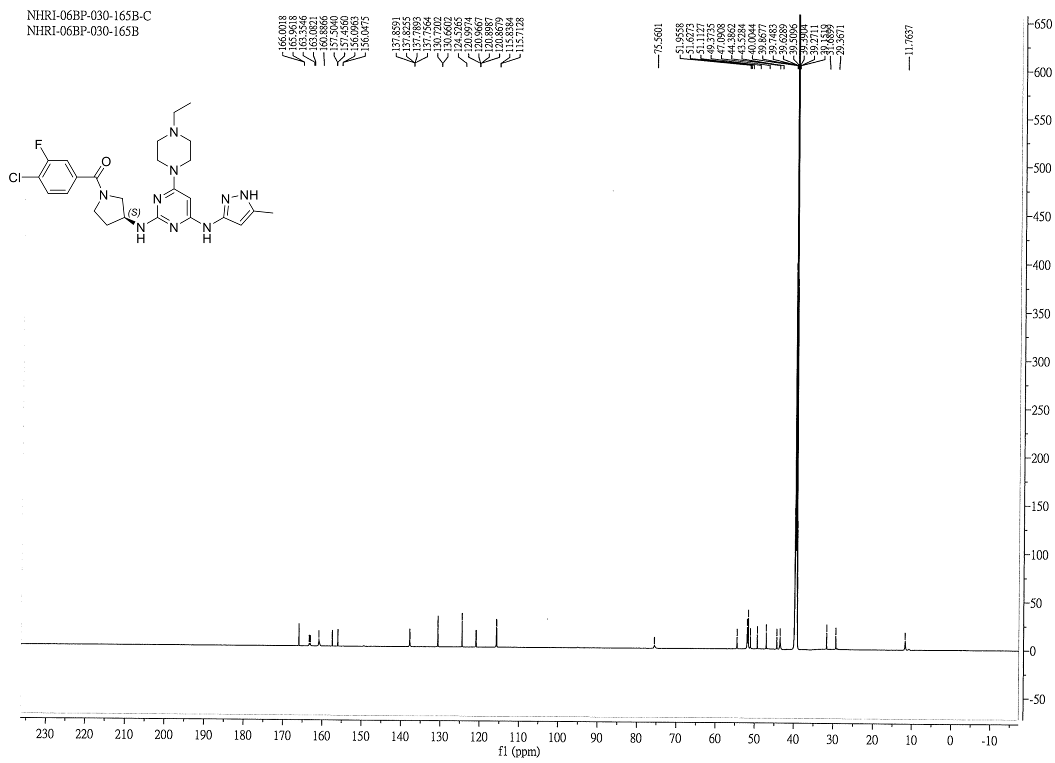

Sample Name: 06BP-030-165B-50   Vial Number: 76
Injection from this vial: 1 of 1   Volume: 20.0 ul
Sample Description:

Chrom Type: Fixed WL Chromatogram, 254 nm

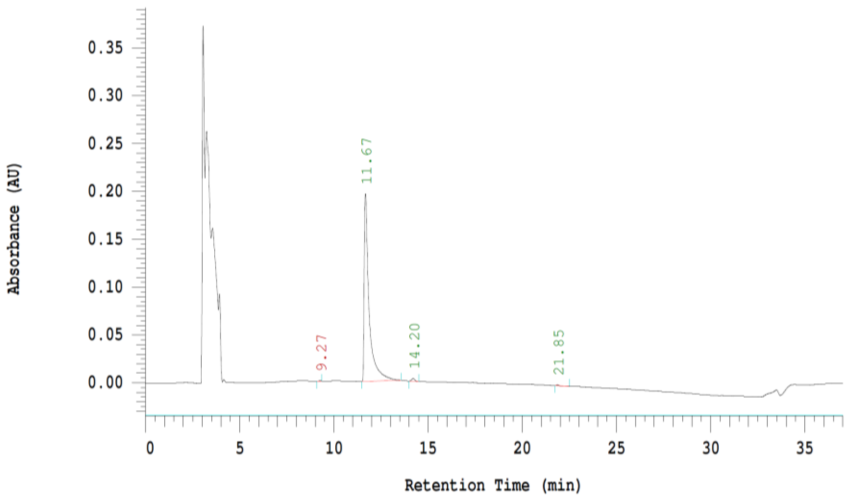

Processing Method: Purity_37min
Column Type: Column   Method Developer: Bob
Method Description:

Peak Quantitation: AREA
Calculation Method: AREA%

No.	RT	Area	Height	Conc 1
1	9.27	935	129	0.053
2	11.67	1747304	97958	98.786
3	14.20	16524	1525	0.934
4	21.85	4014	362	0.227
		1768777	99974	100.000

Peak rejection level: 0

Figure S13. NMR and HPLC spectra of compound 11.

(*S*)-(4-chloro-3-methylphenyl)(3-((4-(4-ethylpiperazin-1-yl)-6-((5-methyl-1H-pyrazol-3-yl)amino)pyrimidin-2-yl)amino)pyrrolidin-1-yl)methanone (11)

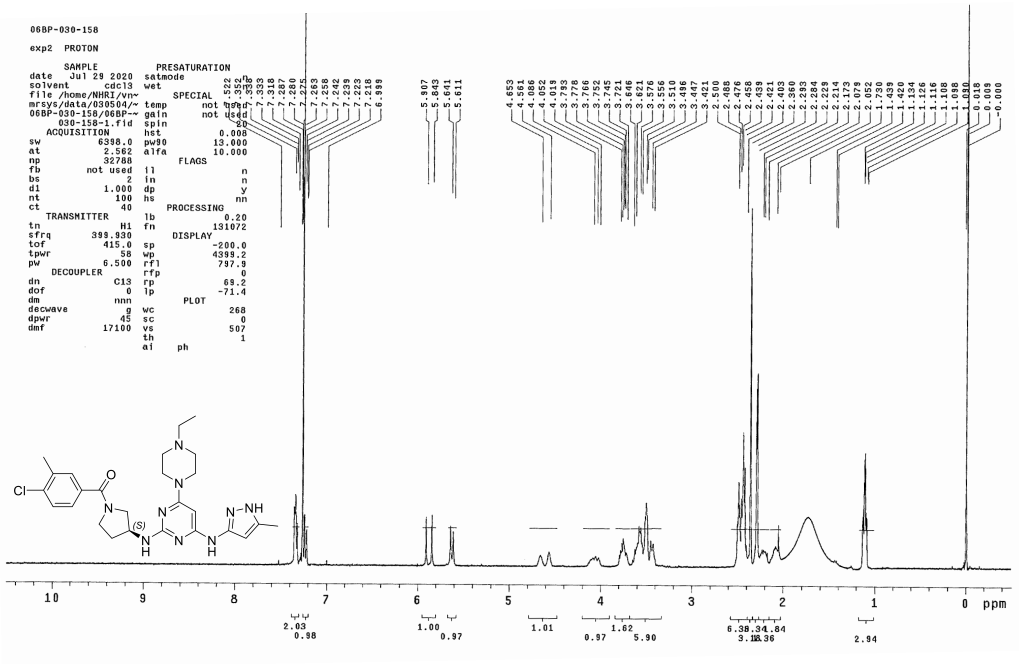

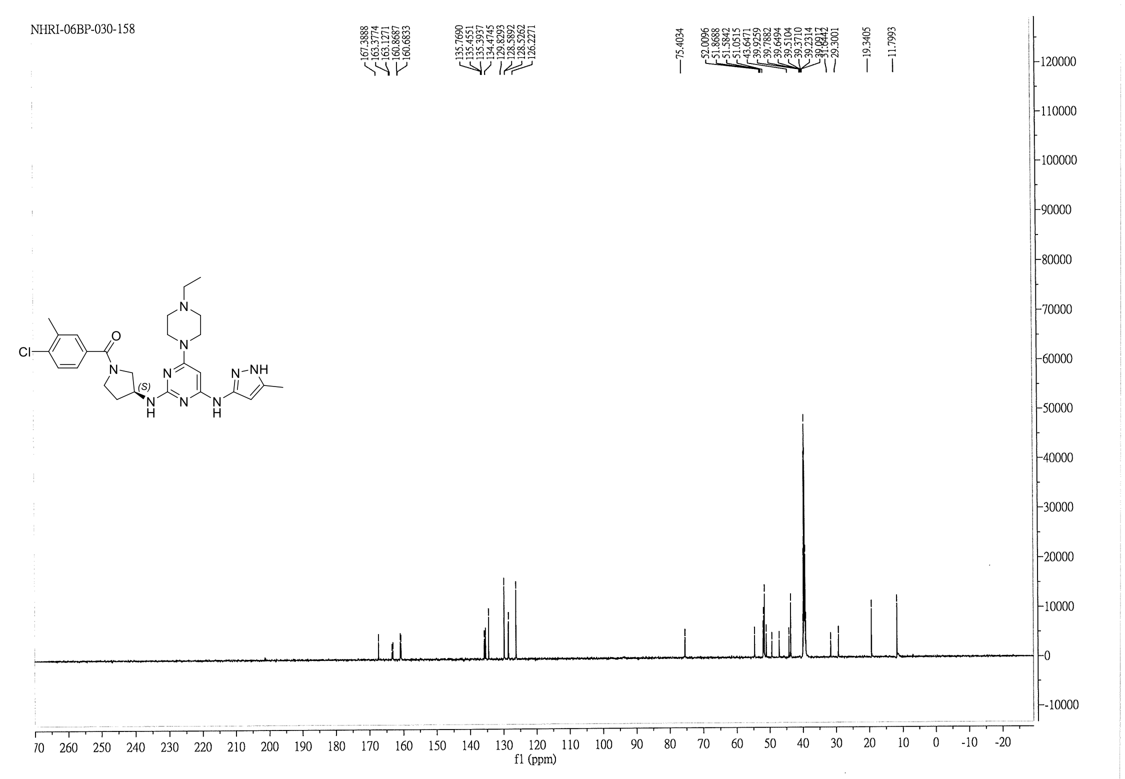

Sample Name: 06BP-030-158-50 Vial Number: 62
Injection from this vial: 1 of 1 Volume: 20.0 ul
Sample Description:

Chrom Type: Fixed WL Chromatogram, 254 nm

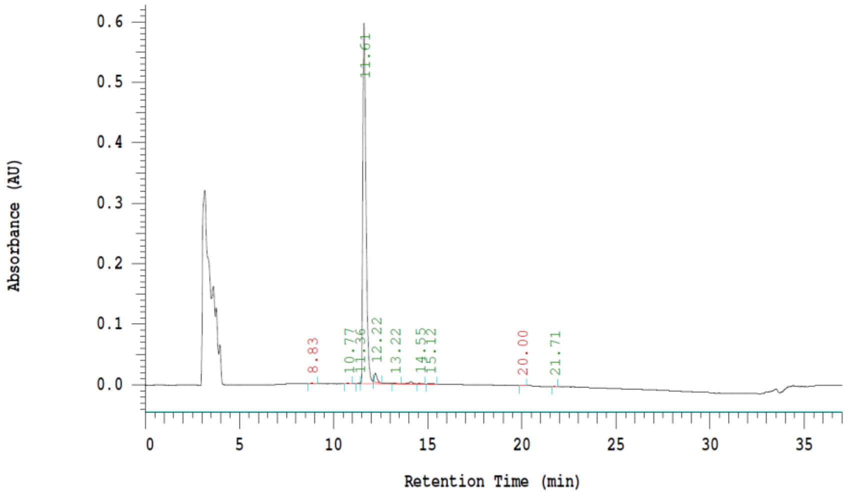

Processing Method: Purity_37min
Column Type: Column Method Developer: Bob
Method Description:

Peak Quantitation: AREA
Calculation Method: AREA%

No.	RT	Area	Height	Conc 1
1	8.83	3461	378	0.096
2	10.77	3020	230	0.083
3	11.36	2568	352	0.071
4	11.61	3523496	297667	97.327
5	12.22	69811	7172	1.928
6	13.22	4761	511	0.132
7	14.55	5766	486	0.159
8	15.12	3168	170	0.088
9	20.00	1436	192	0.040
10	21.71	2760	328	0.076
		3620247	307486	100.000

Peak rejection level: 0

Figure S14. NMR and HPLC spectra of compound 12.

(*S*)-(5-chloro-2-fluorophenyl)(3-((4-(4-ethylpiperazin-1-yl)-6-((5-methyl-1H-pyrazol-3-yl)amino)pyrimidin-2-yl)amino)pyrrolidin-1-yl)methanone (12)

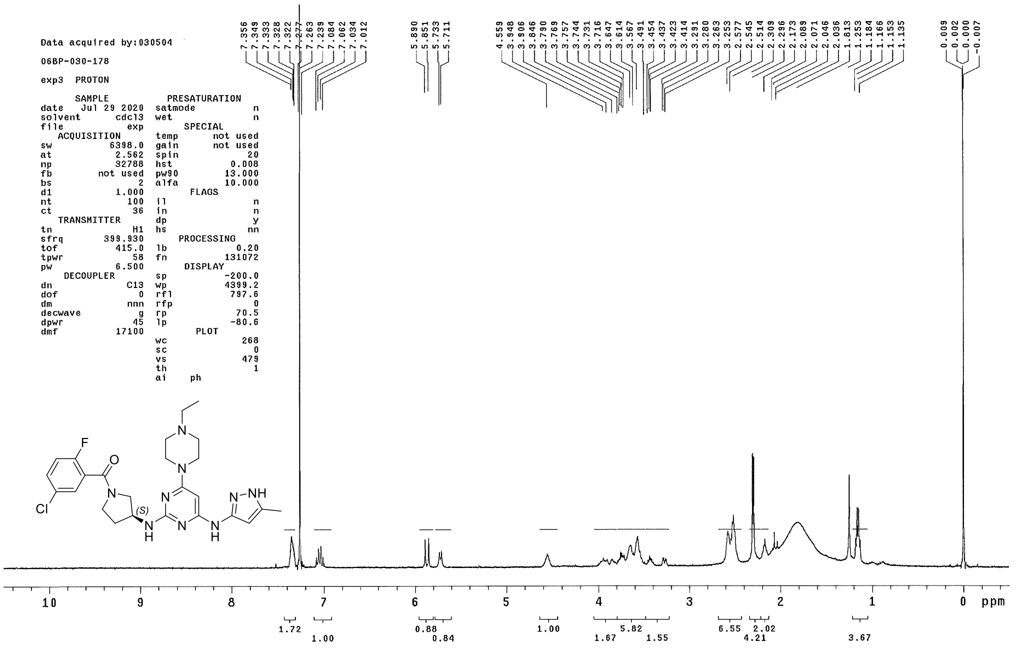

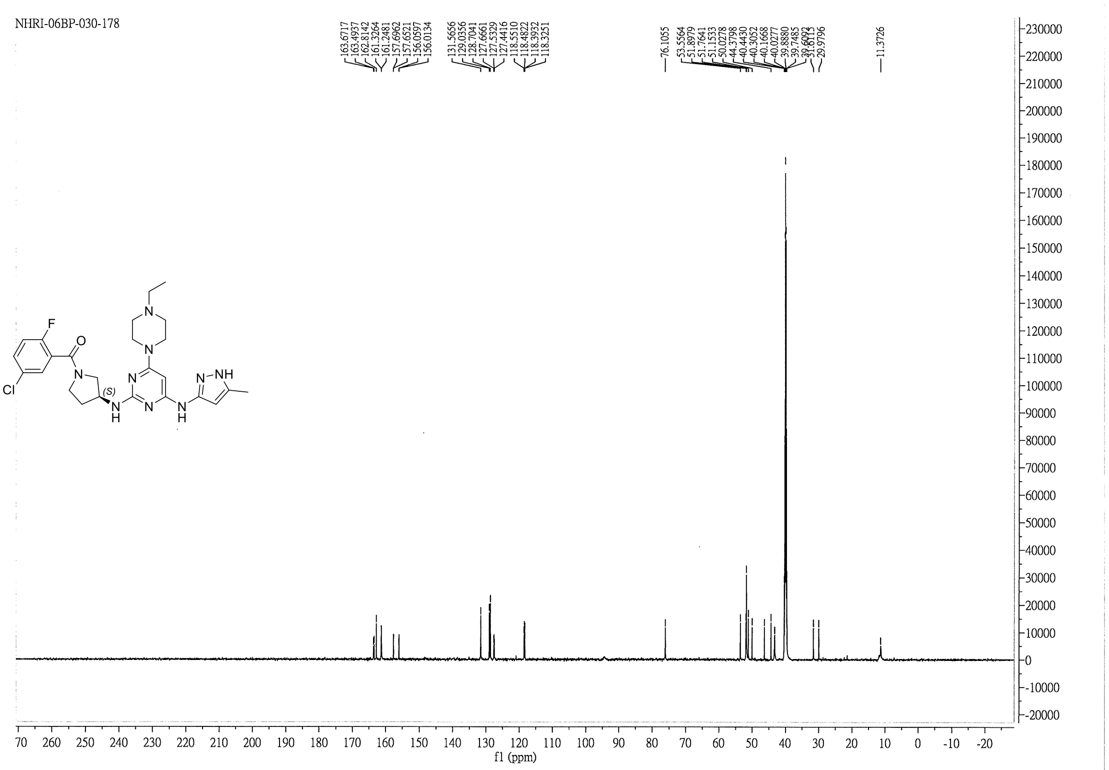

Sample Name: 06BP-030-178-50
Vial Number: 66
Injection from this vial: 1 of 1
Volume: 20.0 ul
Sample Description:

Chrom Type: Fixed WL Chromatogram, 254 nm

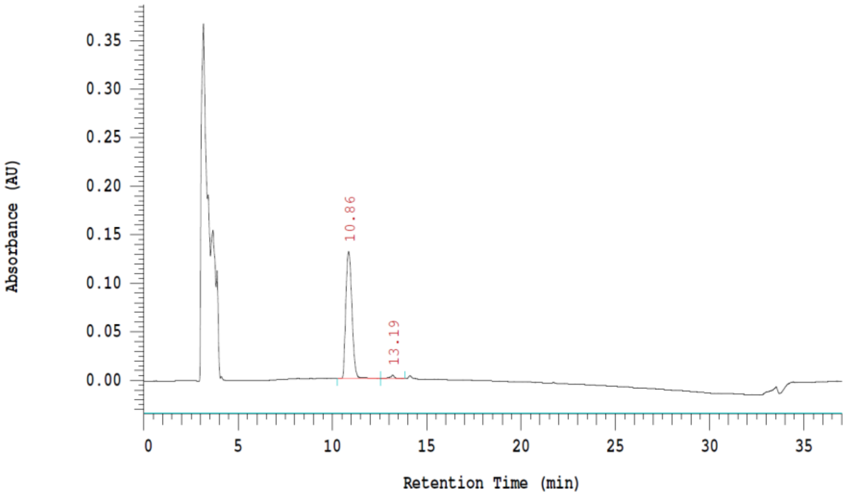

Processing Method: Purity_37min
Column Type: Column
Method Developer: Bob
Method Description:

Peak Quantitation: AREA
Calculation Method: AREA%

No.	RT	Area	Height	Conc 1
1	10.86	1451846	65396	97.888
2	13.19	31331	1831	2.112
		1483177	67227	100.000

Peak rejection level: 0

Figure S15. NMR and HPLC spectra of compound 13.

(*S*)-(4-chloro-2-fluorophenyl)(3-((4-(4-ethylpiperazin-1-yl)-6-((5-methyl-1H-pyrazol-3-yl)amino)pyrimidin-2-yl)amino)pyrrolidin-1-yl)methanone (13)

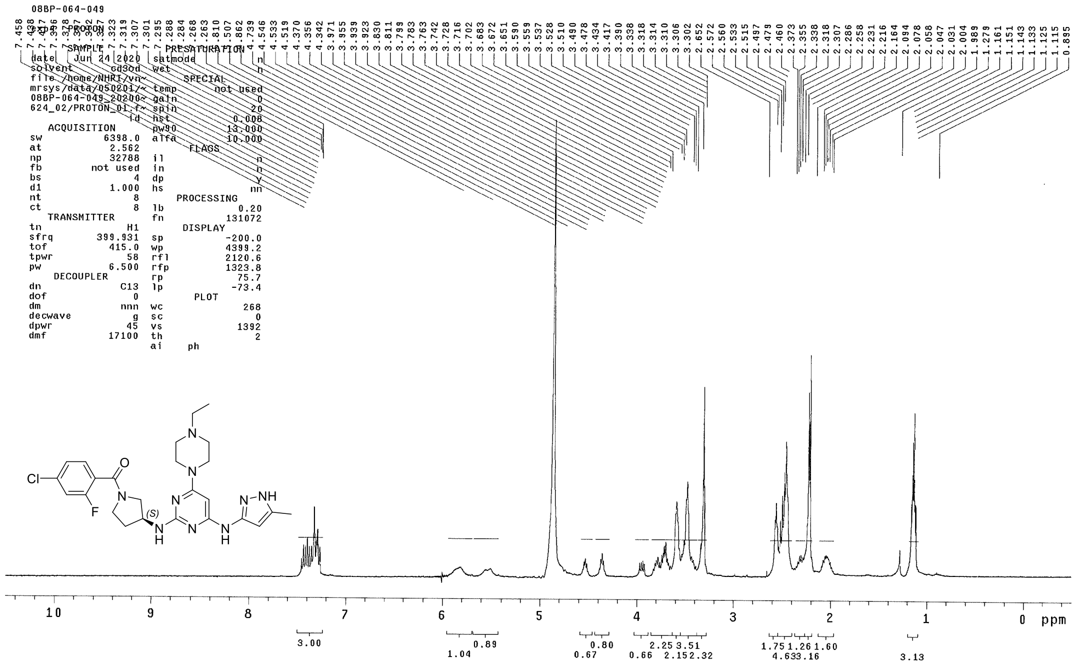

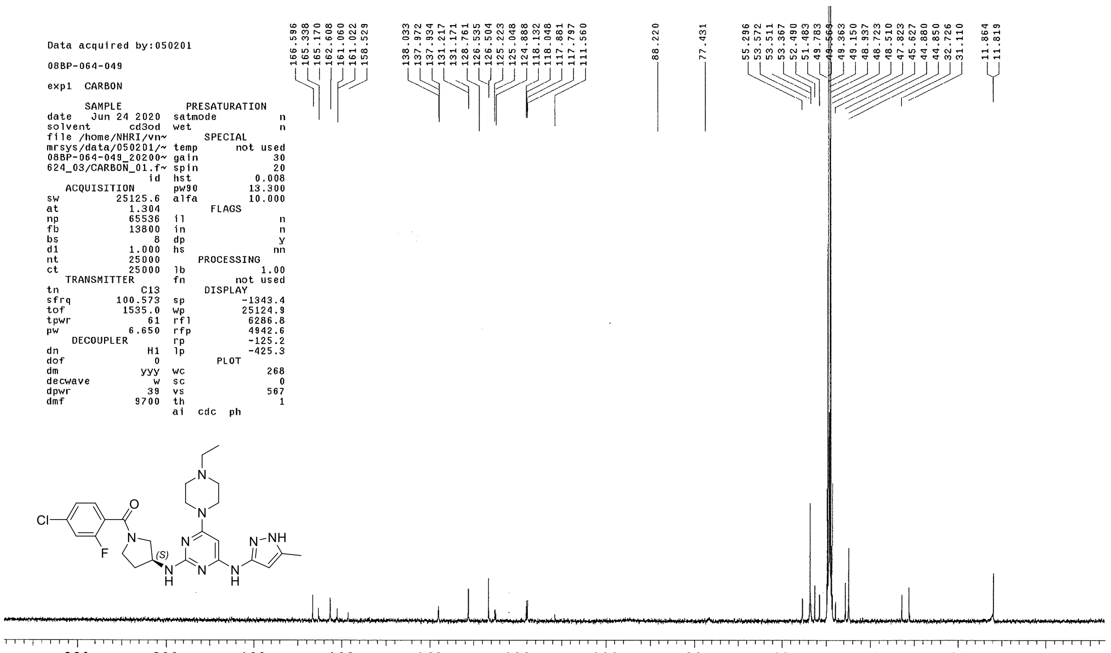

Sample Name: 08BP-064-049-50 Vial Number: 160
Injection from this vial: 1 of 1 Volume: 20.0 ul
Sample Description:

Chrom Type: Fixed WL Chromatogram, 254 nm

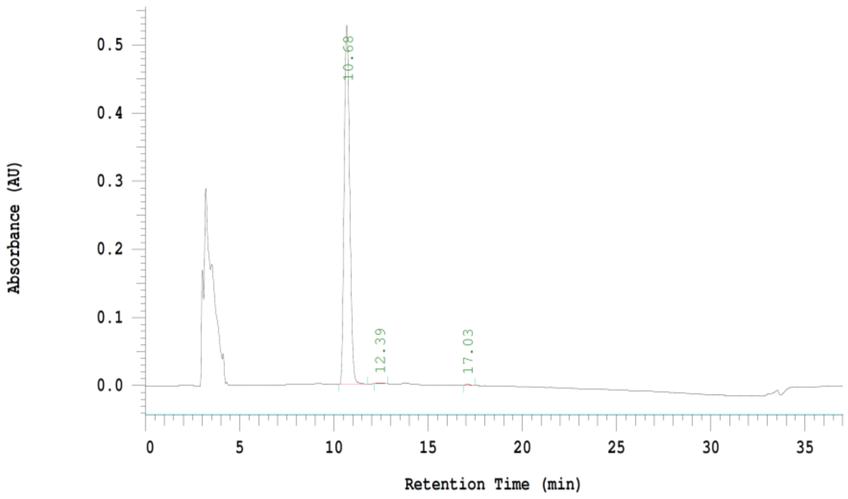

Processing Method: Purity_37min
Column Type: Column Method Developer: Bob
Method Description:

Peak Quantitation: AREA
Calculation Method: AREA%

No.	RT	Area	Height	Conc 1
1	10.68	5307798	263279	99.573
2	12.39	6130	232	0.115
3	17.03	16648	984	0.312
		5330576	264495	100.000

Peak rejection level: 0

Figure S16. NMR and HPLC spectra of compound 14.

(*S*)-(3-((4-(4-ethylpiperazin-1-yl)-6-((5-methyl-1H-pyrazol-3-yl)amino)pyrimidin-2-yl)amino)pyrrolidin-1-yl)(2-fluoro-4-(trifluoromethyl)phenyl)methanone (14)

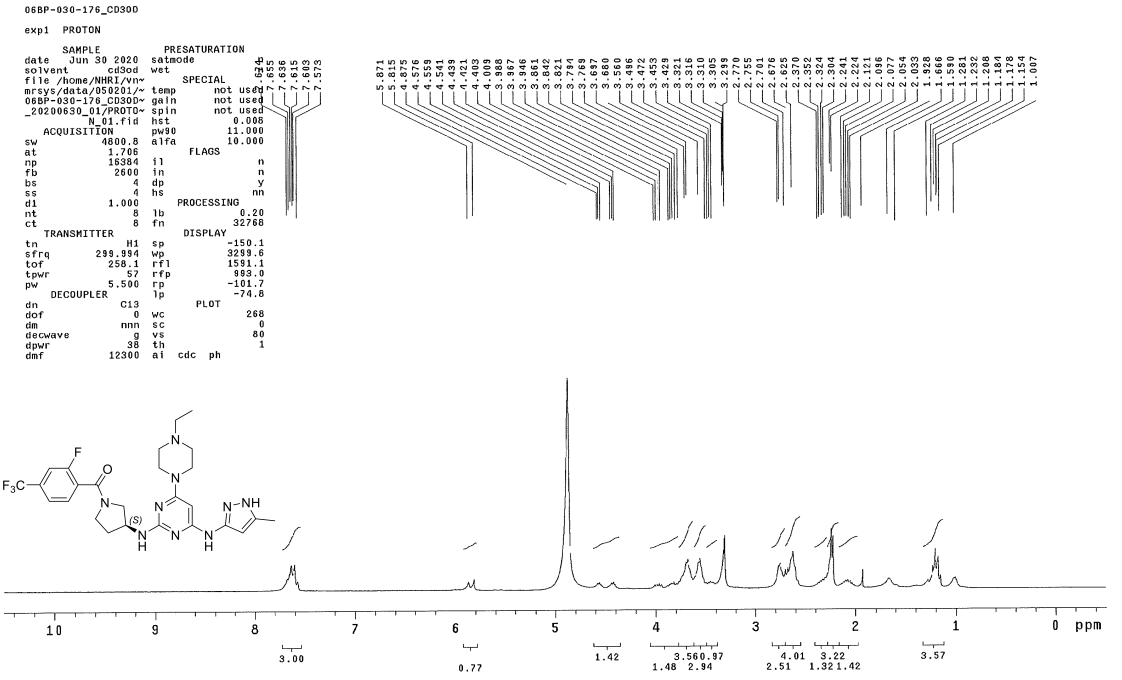

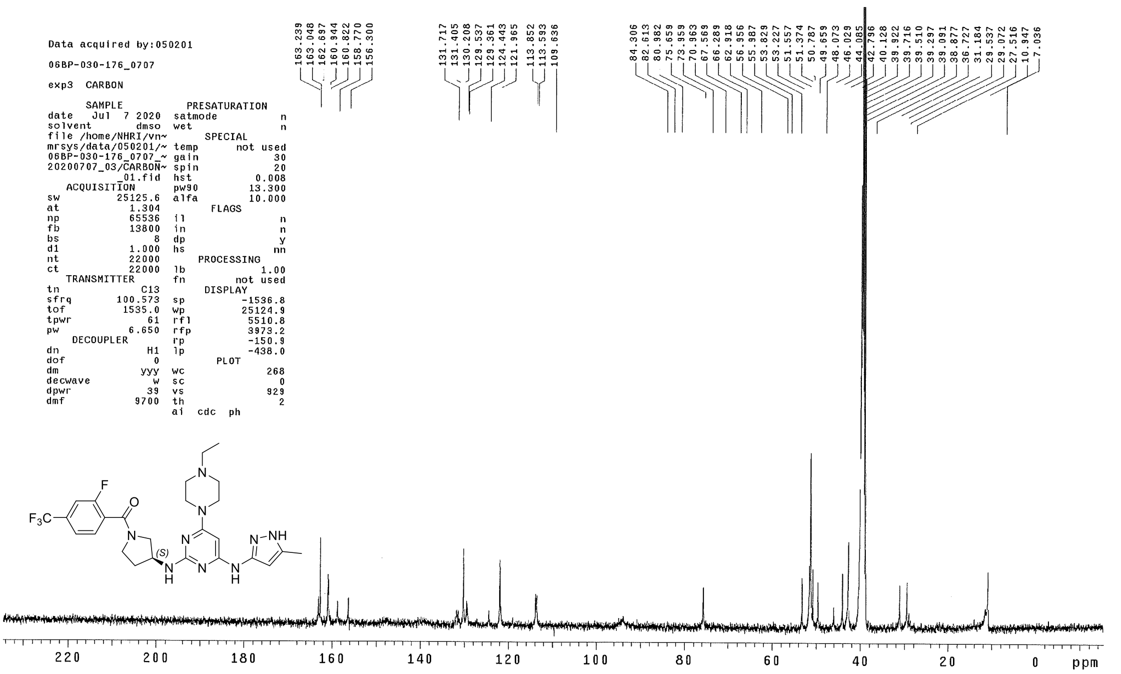

Sample Name: 06BP-030-176-50   Vial Number: 116
Injection from this vial: 1 of 1   Volume: 20.0 ul
Sample Description:

Chrom Type: Fixed WL Chromatogram, 254 nm

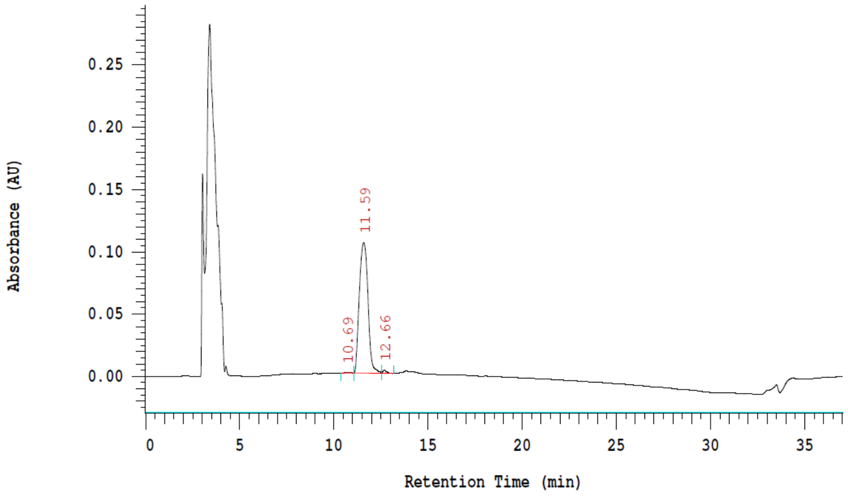

Processing Method: Purity_37min
Column Type: Column   Method Developer: Bob
Method Description:

Peak Quantitation: AREA
Calculation Method: AREA%

No.	RT	Area	Height	Conc 1
1	10.69	4455	175	0.264
2	11.59	1665806	52411	98.605
3	12.66	19113	1360	1.131
		1689374	53946	100.000

Peak rejection level: 0

Figure S17. NMR and HPLC spectra of compound 15.

(*S*)-(4-chloro-2,6-difluorophenyl)(3-((4-(4-ethylpiperazin-1-yl)-6-((5-methyl-1H-pyrazol-3-yl)amino)pyrimidin-2-yl)amino)pyrrolidin-1-yl)methanone (15)

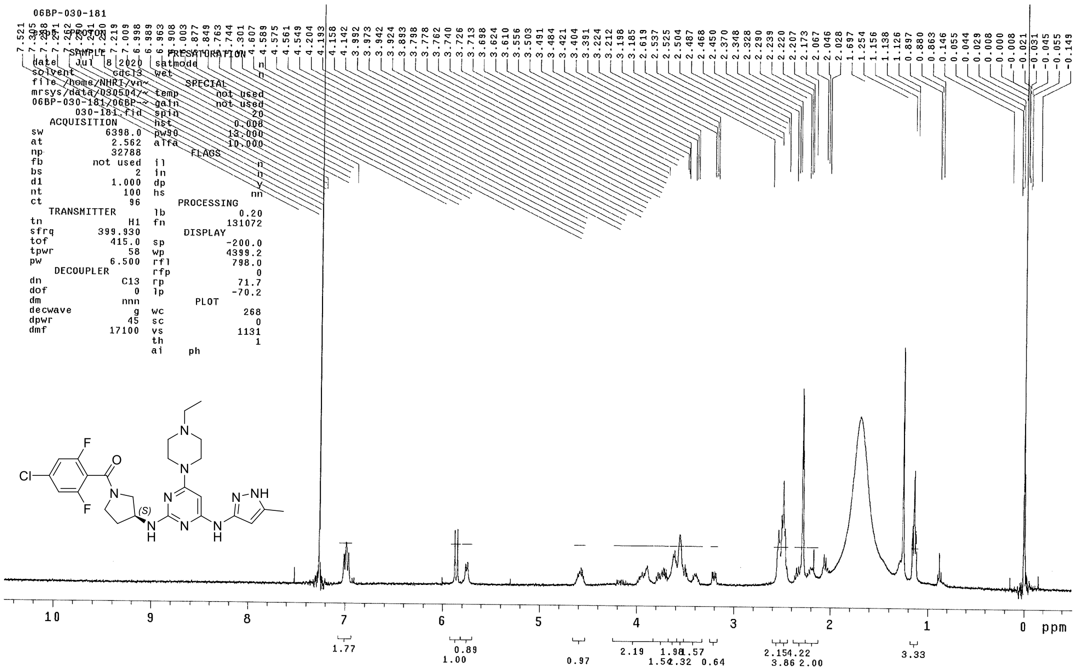

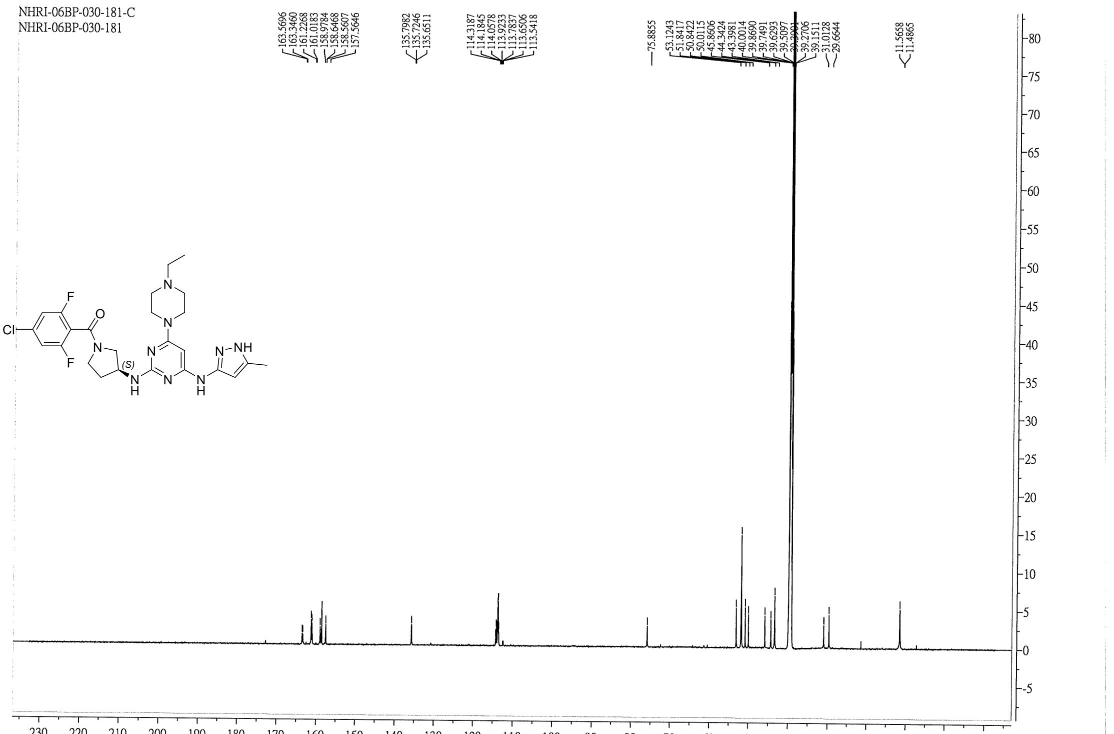

Sample Name: 06BP-030-181-50
Vial Number: 120
Injection from this vial: 1 of 1
Volume: 20.0 ul
Sample Description:

Chrom Type: Fixed WL Chromatogram, 254 nm

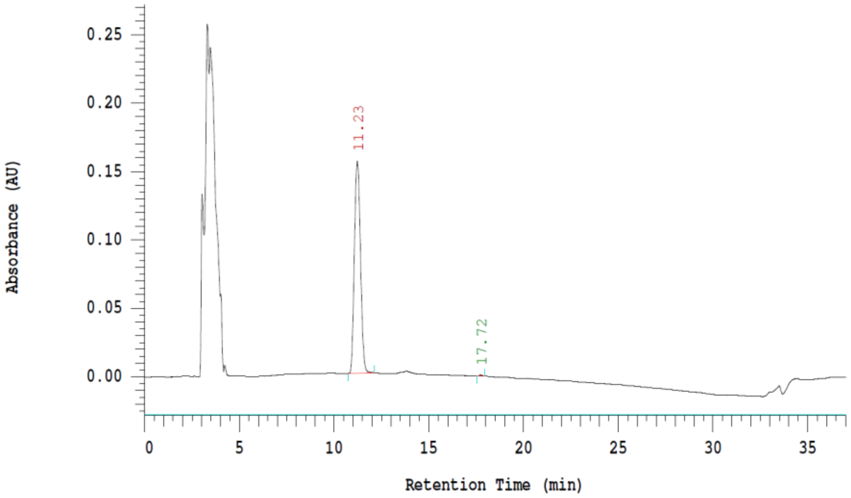

Processing Method: Purity_37min
Column Type: Column
Method Developer: Bob
Method Description:

Peak Quantitation: AREA
Calculation Method: AREA%

No.	RT	Area	Height	Conc 1
1	11.23	1710714	77480	99.745
2	17.72	4377	428	0.255
		1715091	77908	100.000

Peak rejection level: 0

Figure S18. NMR and HPLC spectra of compound 16.

(*S*)-(4-chloro-2,5-difluorophenyl)(3-((4-(4-ethylpiperazin-1-yl)-6-((5-methyl-1H-pyrazol-3-yl)amino)pyrimidin-2-yl)amino)pyrrolidin-1-yl)methanone (16)

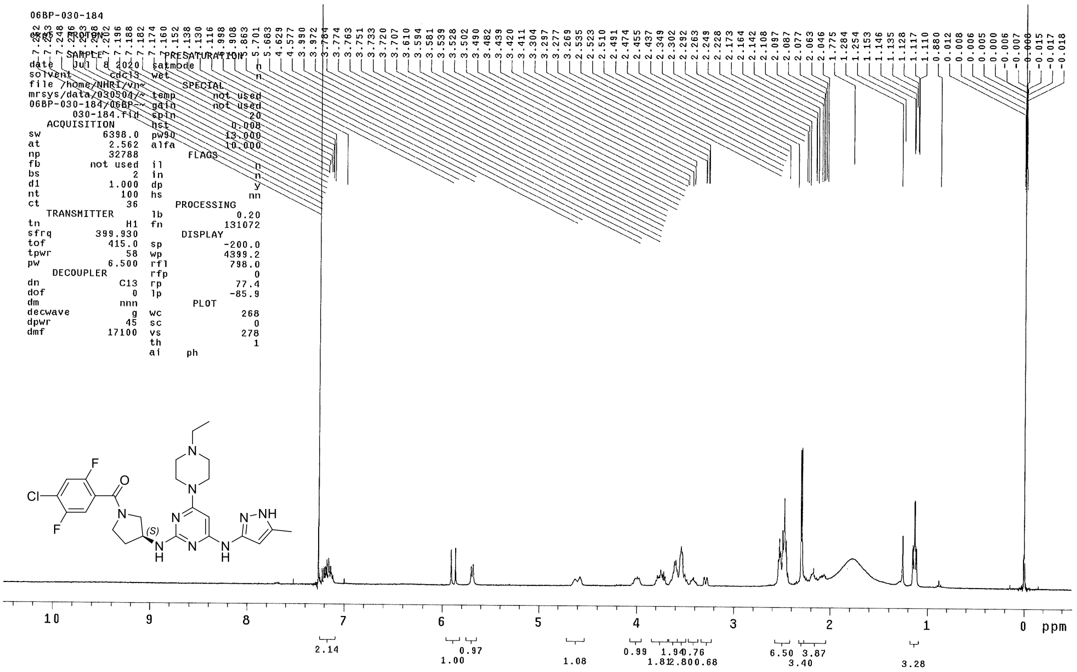

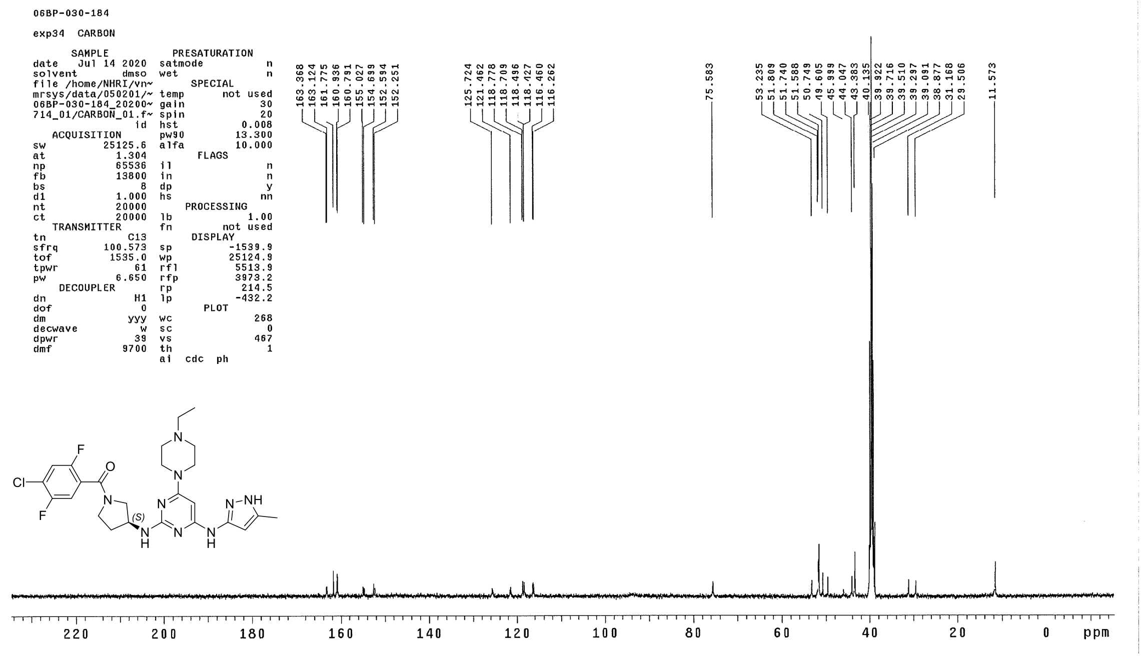

Sample Name: 06BP-030-184-50 Vial Number: 124
Injection from this vial: 1 of 1 Volume: 20.0 ul
Sample Description:

Chrom Type: Fixed WL Chromatogram, 254 nm

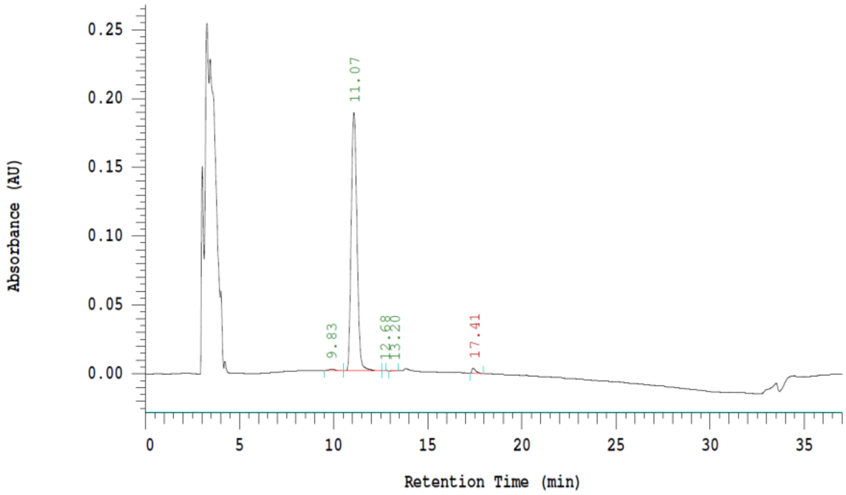

Processing Method: Purity_37min
Column Type: Column Method Developer: Bob
Method Description:

Peak Quantitation: AREA
Calculation Method: AREA%

No.	RT	Area	Height	Conc 1
1	9.83	11809	546	0.587
2	11.07	1971673	93614	97.981
3	12.68	634	106	0.032
4	13.20	2412	164	0.120
5	17.41	25770	1845	1.281
		2012298	96275	100.000

Peak rejection level: 0

Figure S19. NMR and HPLC spectra of compound 17.

(*S*)-(4-chloro-2,3-difluorophenyl)(3-((4-(4-ethylpiperazin-1-yl)-6-((5-methyl-1H-pyrazol-3-yl)amino)pyrimidin-2-yl)amino)pyrrolidin-1-yl)methanone (17)

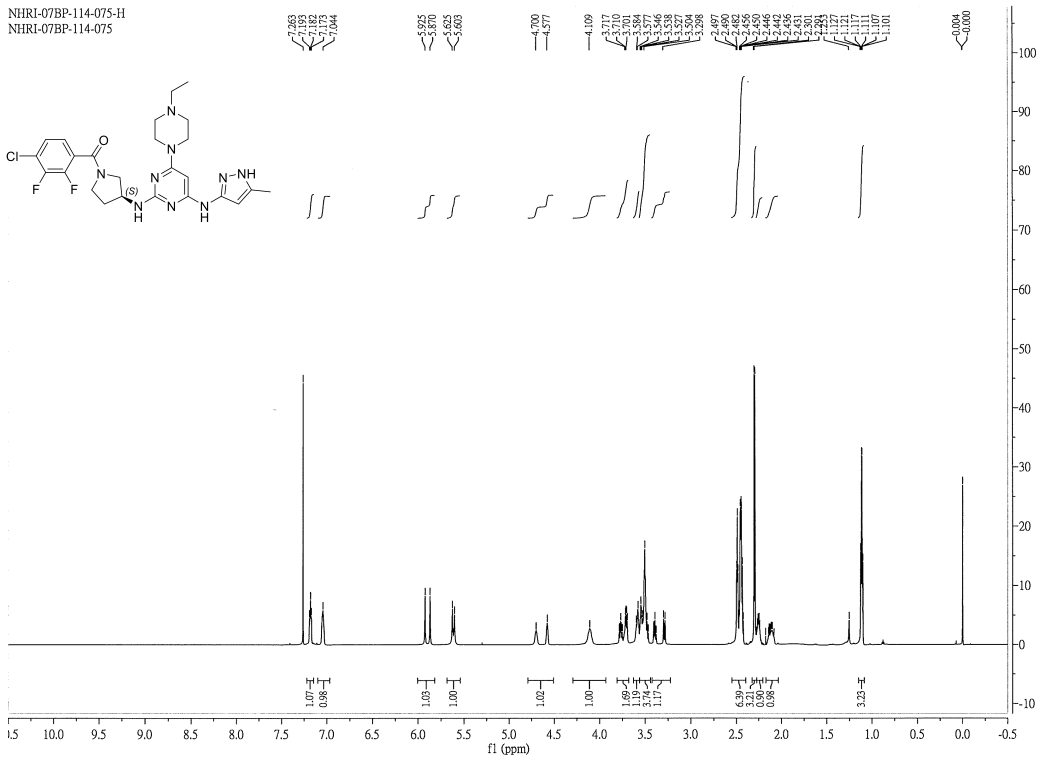

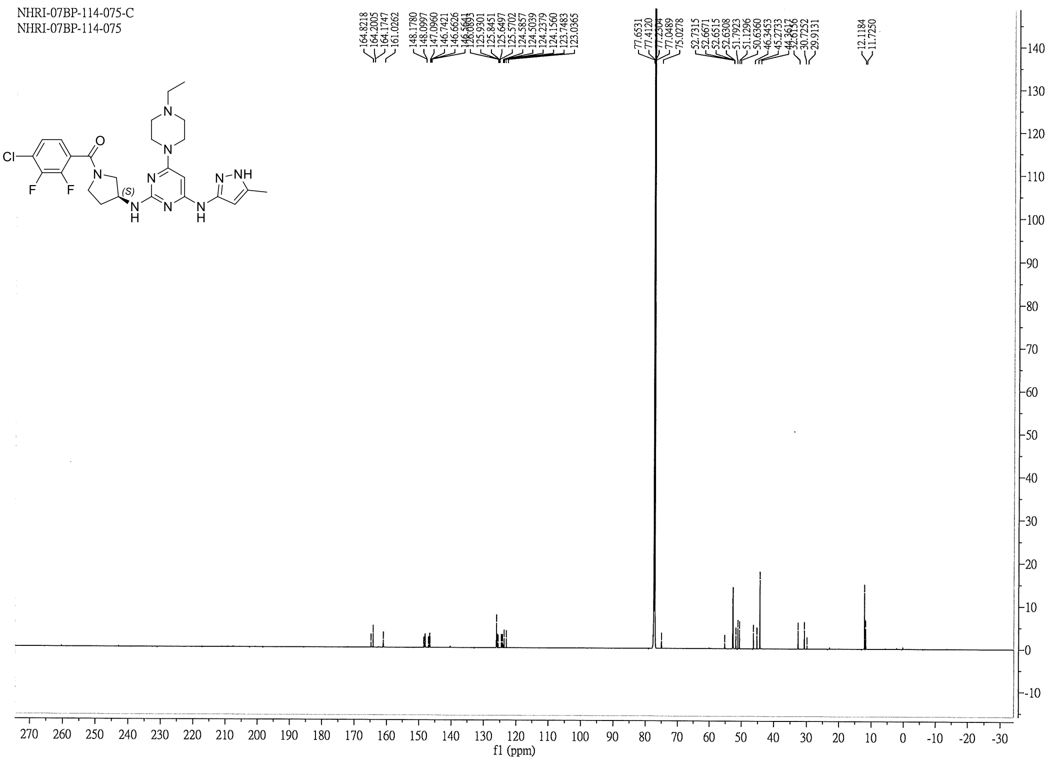

Sample Name: 07BP-114-075-50
Injection from this vial: 1 of 1
Sample Description:

Vial Number: 30
Volume: 20.0 ul

Chrom Type: Fixed WL Chromatogram, 254 nm

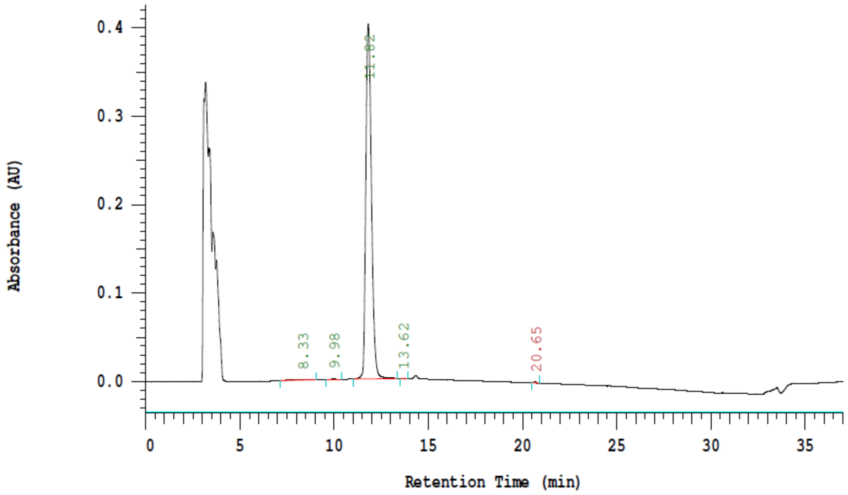

Processing Method: Purity_37min
Column Type: Column
Method Description:

Method Developer: Bob

Peak Quantitation: AREA
Calculation Method: AREA%

No.	RT	Area	Height	Conc 1
1	8.33	22832	315	0.548
2	9.98	3663	233	0.088
3	11.82	4128163	200684	99.163
4	13.62	1867	135	0.045
5	20.65	6479	794	0.156
		4163004	202161	100.000

Peak rejection level: 0

Figure S20. NMR and HPLC spectra of compound 18.

(*S*)-(4-chloro-3,5-difluorophenyl)(3-((4-(4-ethylpiperazin-1-yl)-6-((5-methyl-1H-pyrazol-3-yl)amino)pyrimidin-2-yl)amino)pyrrolidin-1-yl)methanone (18)

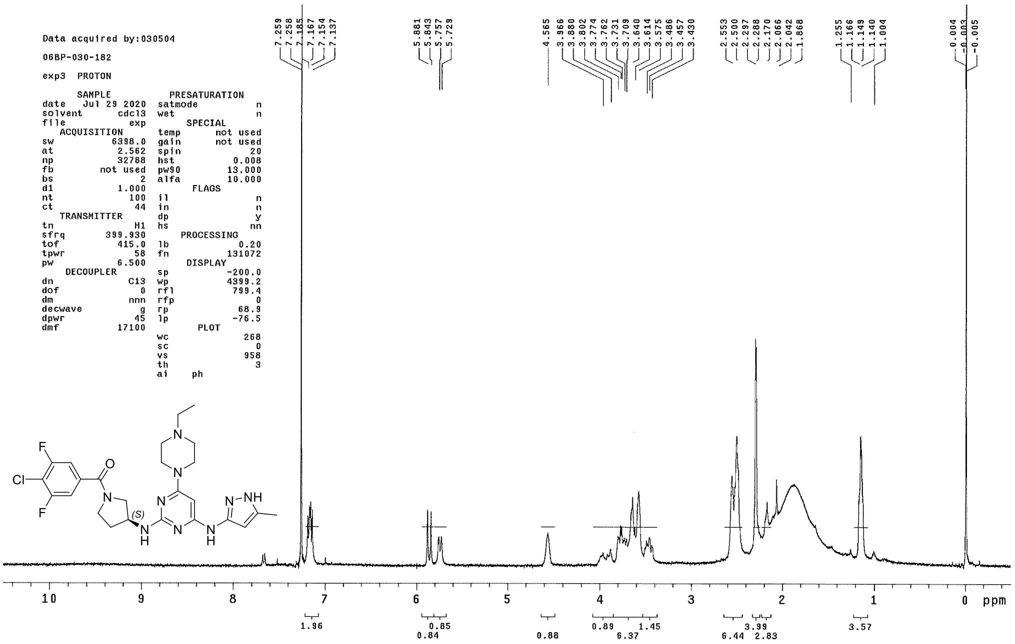

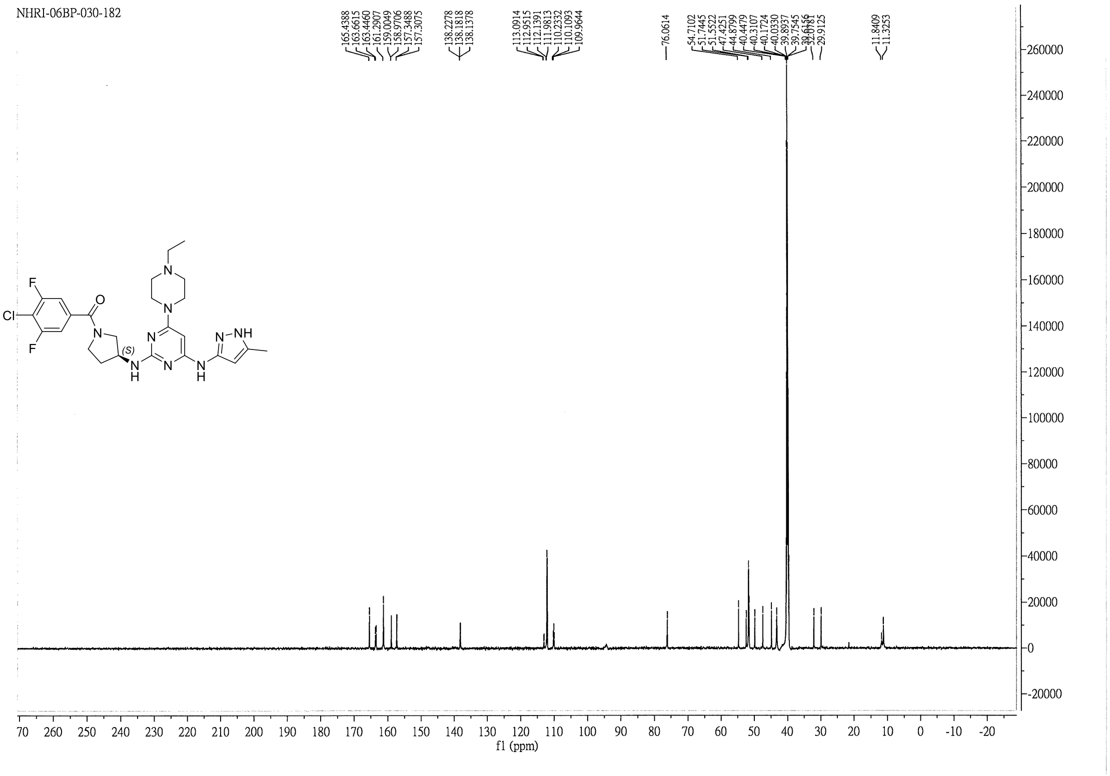

Sample Name: 06BP-030-182-50
Vial Number: 152
Injection from this vial: 1 of 1
Volume: 20.0 ul
Sample Description:

Chrom Type: Fixed WL Chromatogram, 254 nm

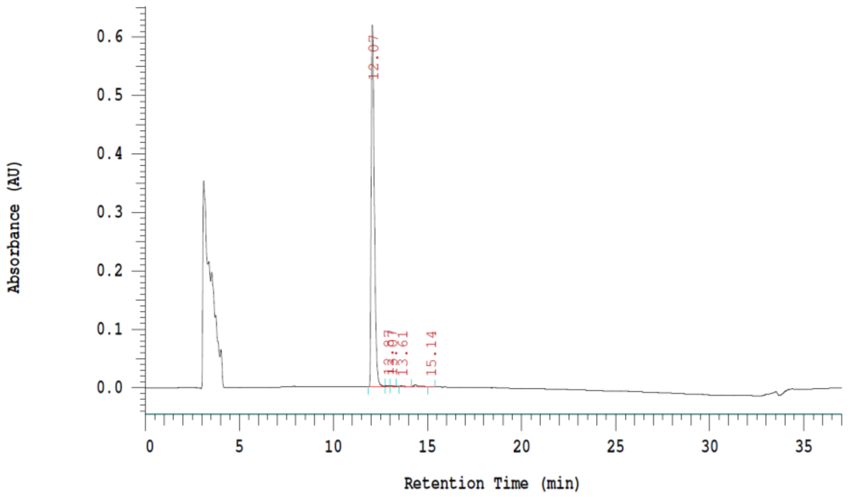

Processing Method: Purity_37min
Column Type: Column
Method Developer: Bob
Method Description:

Peak Quantitation: AREA
Calculation Method: AREA%

No.	RT	Area	Height	Conc 1
1	12.07	3542144	309584	98.778
2	12.87	13516	1111	0.377
3	13.07	12178	957	0.340
4	13.61	14727	929	0.411
5	15.14	3384	285	0.094
		3585949	312866	100.000

Peak rejection level: 0

Figure S21. NMR and HPLC spectra of compound 19.

(*S*)-(3-((4-(4-ethylpiperazin-1-yl)-6-((5-methyl-1H-pyrazol-3-yl)amino)pyrimidin-2-yl)amino)pyrrolidin-1-yl)(2,4,5-trifluorophenyl)methanone (19)

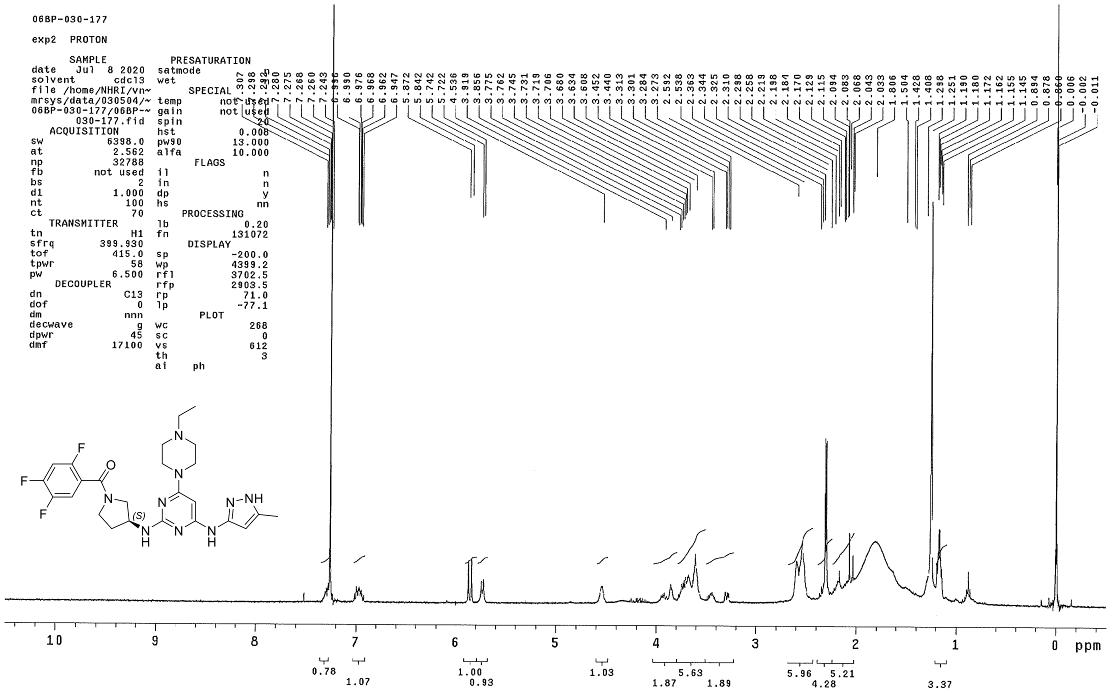

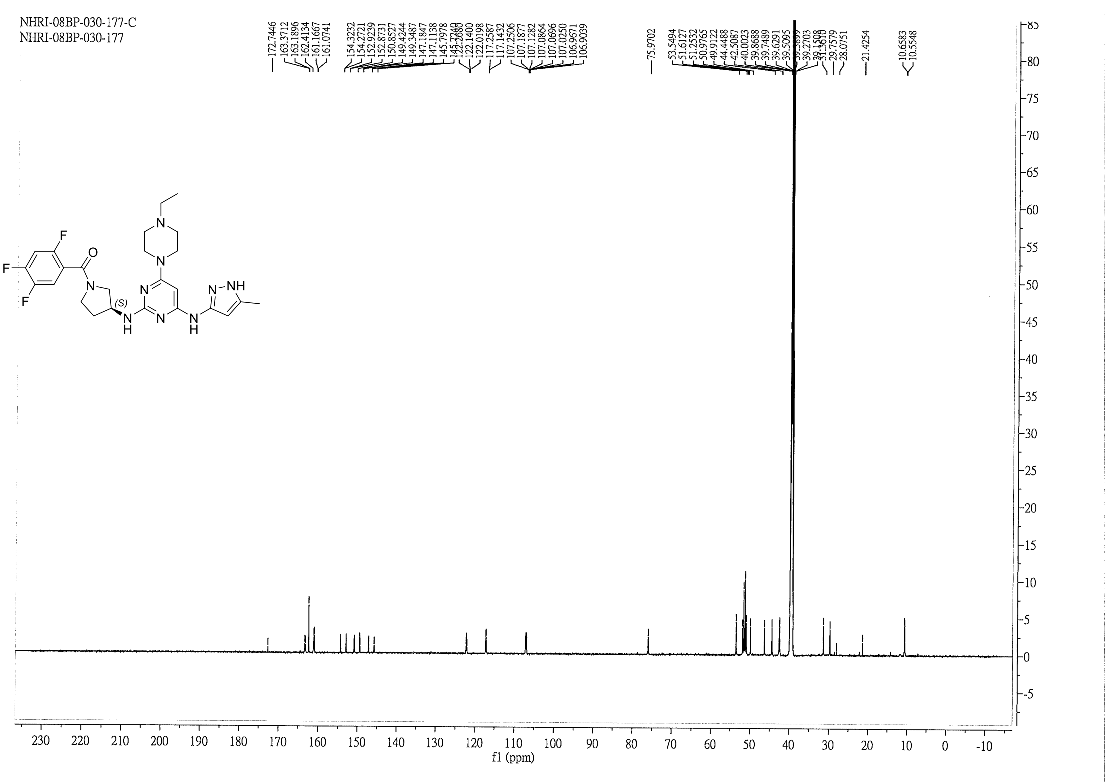

Sample Name: 06BP-030-177-50 Vial Number: 118
Injection from this vial: 1 of 1 Volume: 20.0 ul
Sample Description:

Chrom Type: Fixed WL Chromatogram, 254 nm

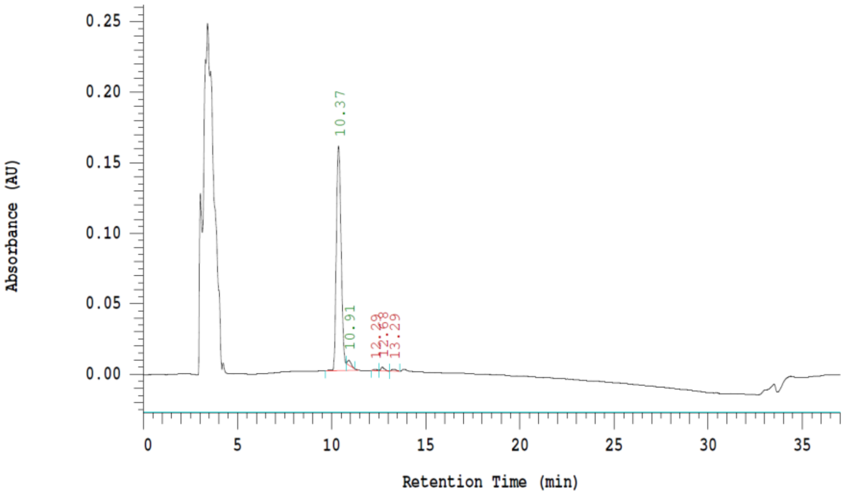

Processing Method: Purity_37min
Column Type: Column Method Developer: Bob
Method Description:

Peak Quantitation: AREA
Calculation Method: AREA%

No.	RT	Area	Height	Conc 1
1	10.37	1438636	79559	96.235
2	10.91	22159	1636	1.482
3	12.29	6921	441	0.463
4	12.68	16414	1251	1.098
5	13.29	10781	691	0.721
		1494911	83578	100.000

Peak rejection level: 0

Figure S22. NMR and HPLC spectra of compound 20.

(*S*)-(6-chloro-2-fluoropyridin-3-yl)(3-((4-(4-ethylpiperazin-1-yl)-6-((5-methyl-1H-pyrazol-3-yl)amino)pyrimidin-2-yl)amino)pyrrolidin-1-yl)methanone (20)

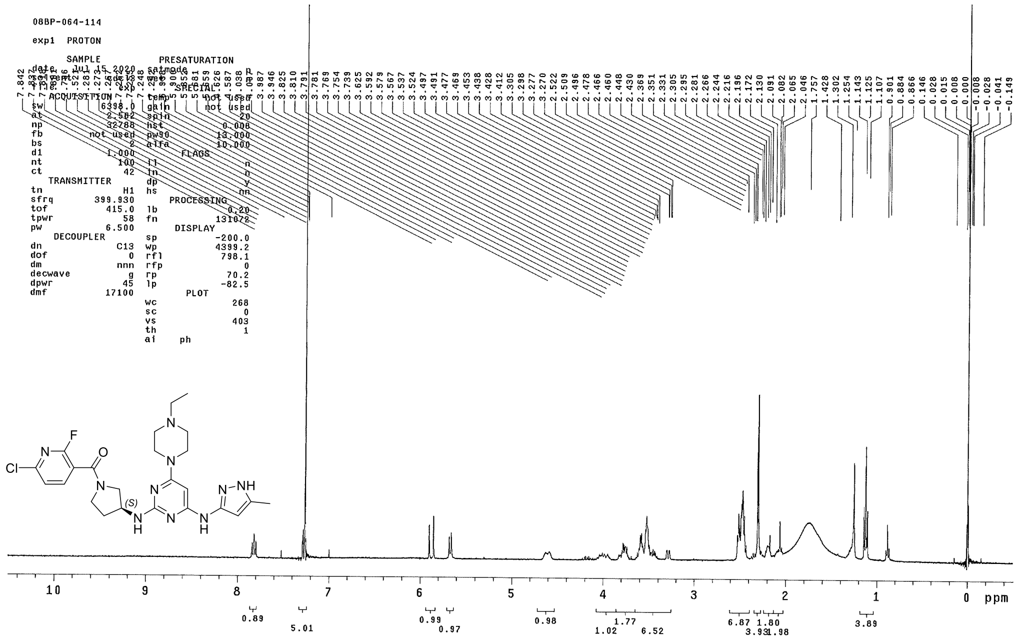

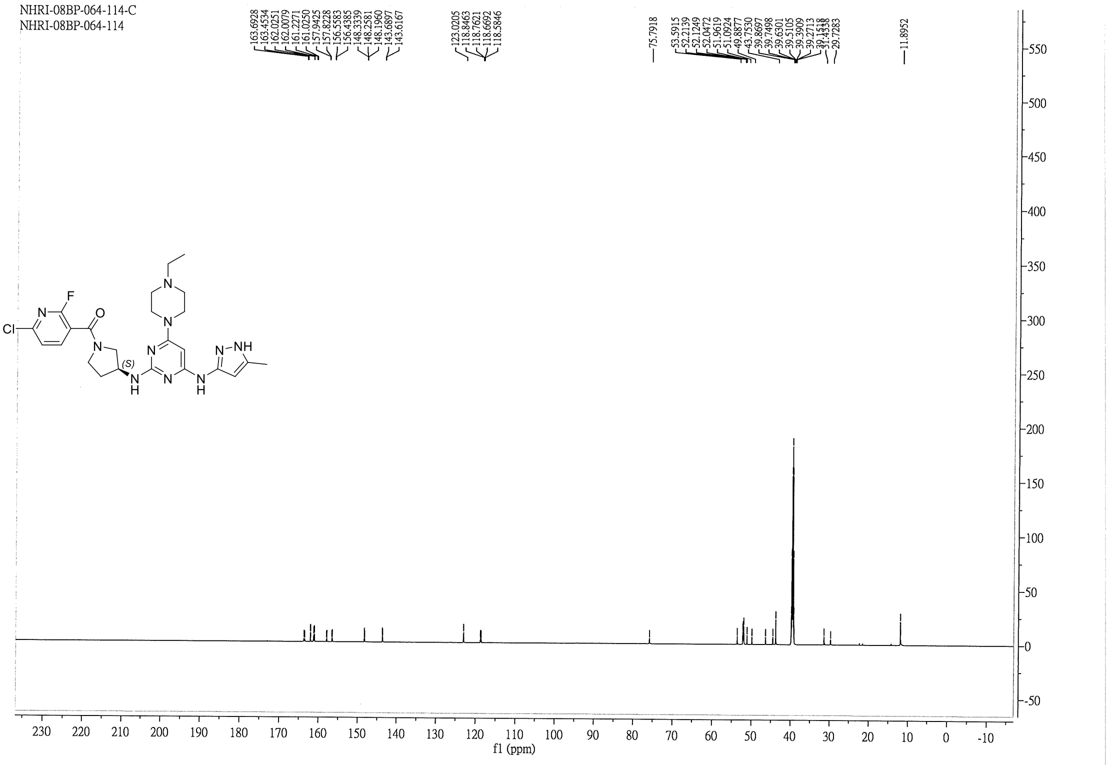

Sample Name: 08BP-064-114-50 Vial Number: 114
Injection from this vial: 1 of 1 Volume: 20.0 ul
Sample Description:

Chrom Type: Fixed WL Chromatogram, 254 nm

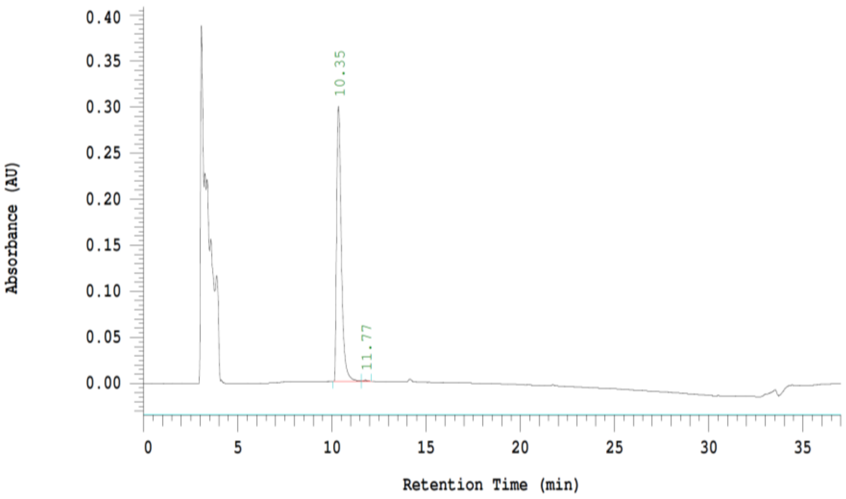

Processing Method: Purity_37min
Column Type: Column Method Developer: Bob
Method Description:

Peak Quantitation: AREA
Calculation Method: AREA%

No.	RT	Area	Height	Conc 1
1	10.35	2563056	149367	99.709
2	11.77	7488	450	0.291
		2570544	149817	100.000

Peak rejection level: 0

Figure S23. NMR and HPLC spectra of compound 21.

(*S*)-N2-(1-((4-chloro-2-fluorophenyl)sulfonyl)pyrrolidin-3-yl)-6-(4-ethylpiperazin-1-yl)-N4-(5-methyl-1H-pyrazol-3-yl)pyrimidine-2,4-diamine (21)

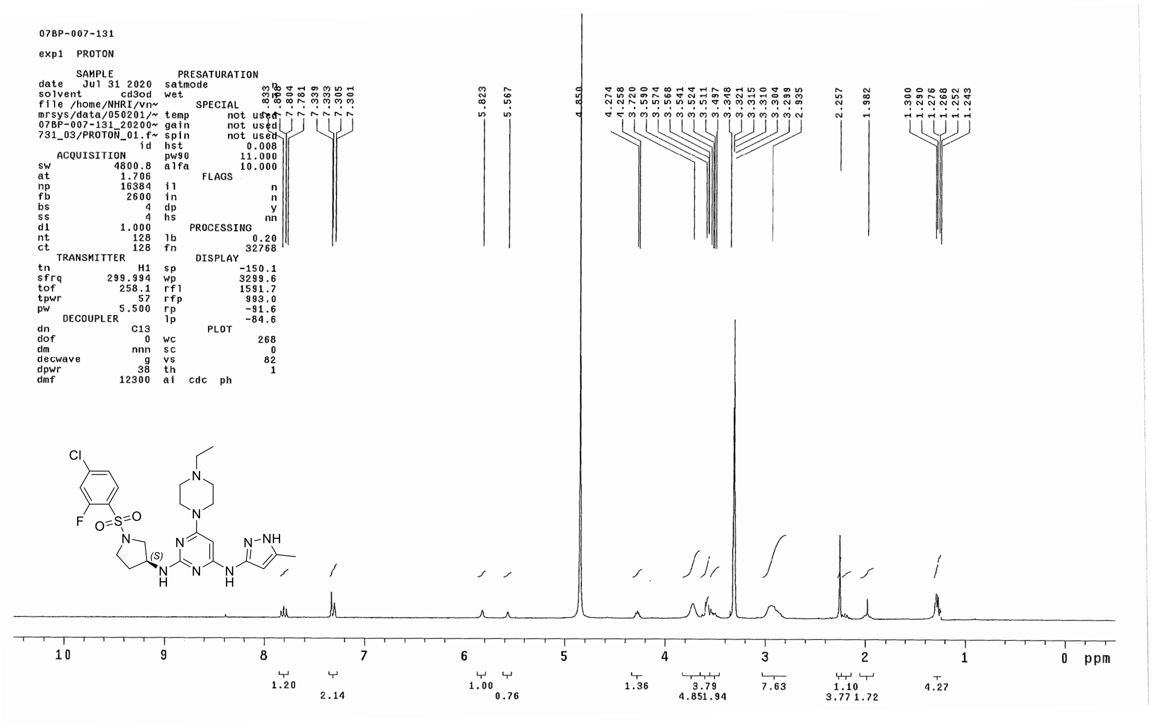

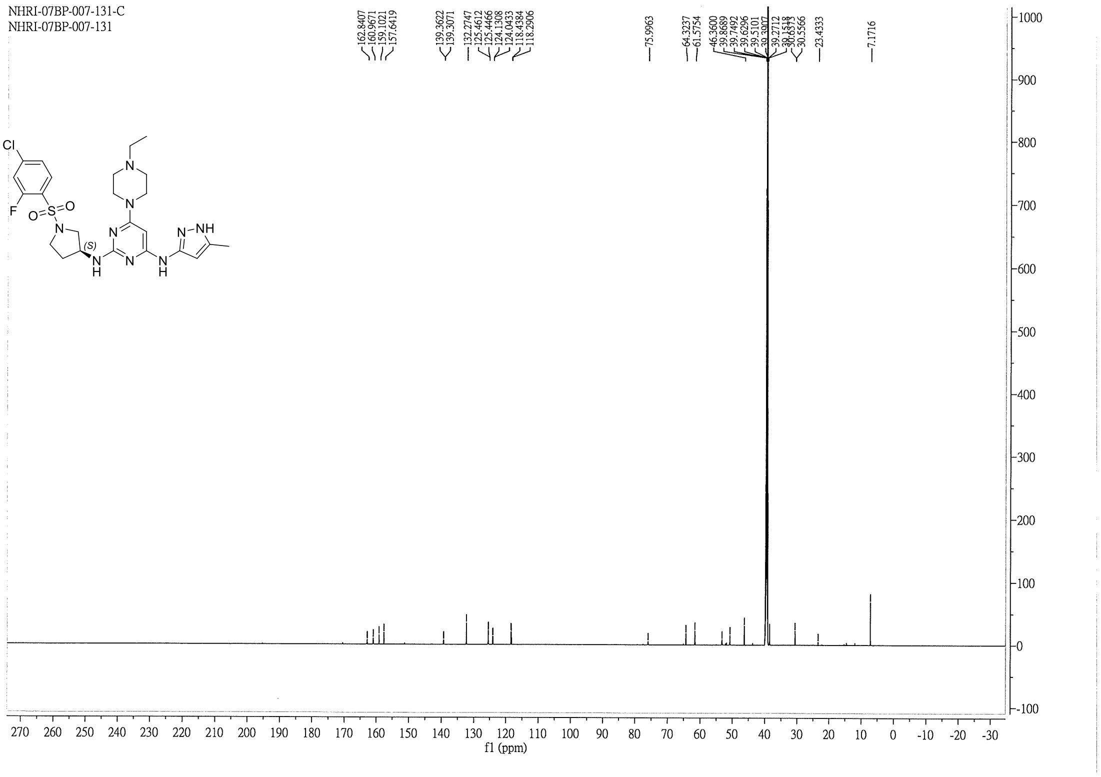

Sample Name: 07BP-007-131-50   Vial Number: 106
Injection from this vial: 1 of 1   Volume: 20.0 ul
Sample Description:

Chrom Type: Fixed WL Chromatogram, 254 nm

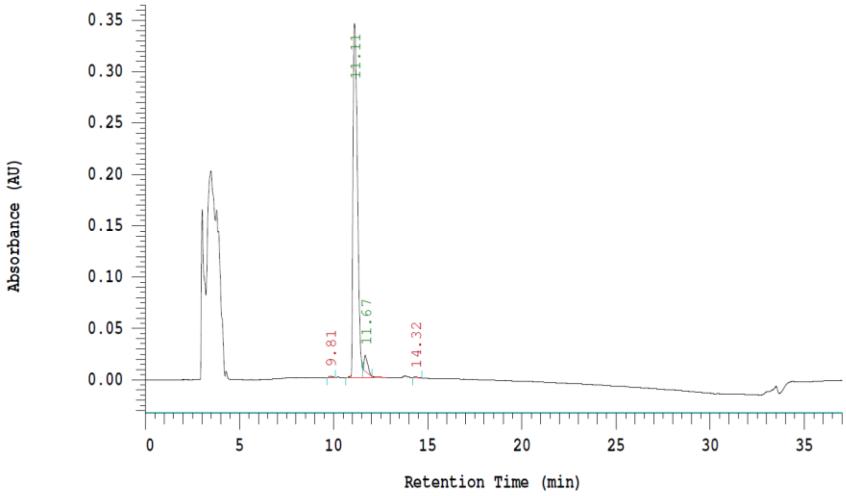

Processing Method: Purity_37min
Column Type: Column   Method Developer: Bob
Method Description:

Peak Quantitation: AREA
Calculation Method: AREA%

No.	RT	Area	Height	Conc 1
1	9.81	8130	651	0.272
2	11.11	2878906	172210	96.337
3	11.67	95794	7855	3.206
4	14.32	5532	334	0.185
		2988362	181050	100.000

Peak rejection level: 0

Figure S24. NMR and HPLC spectra of compound 22.

Ethyl (*S*)-5-((2-((1-(4-chloro-2-fluorobenzoyl)pyrrolidin-3-yl)amino)-6-(4-ethylpiperazin-1-yl)pyrimidin-4-yl)amino)-3-methyl-1H-pyrazole-1-carboxylate (22)

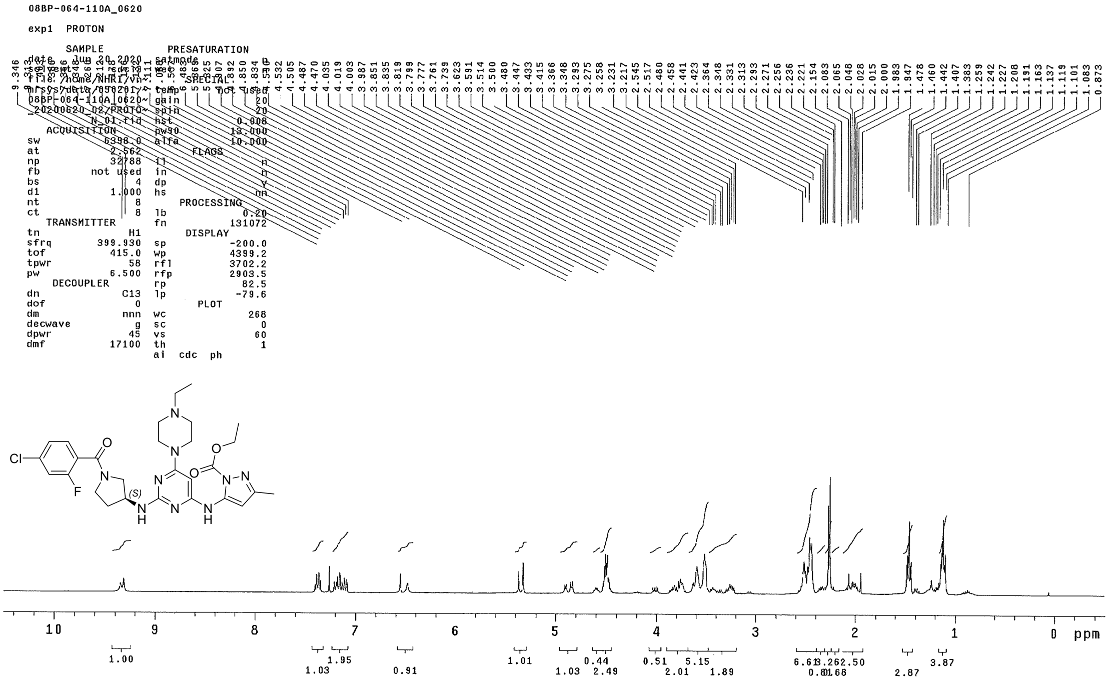

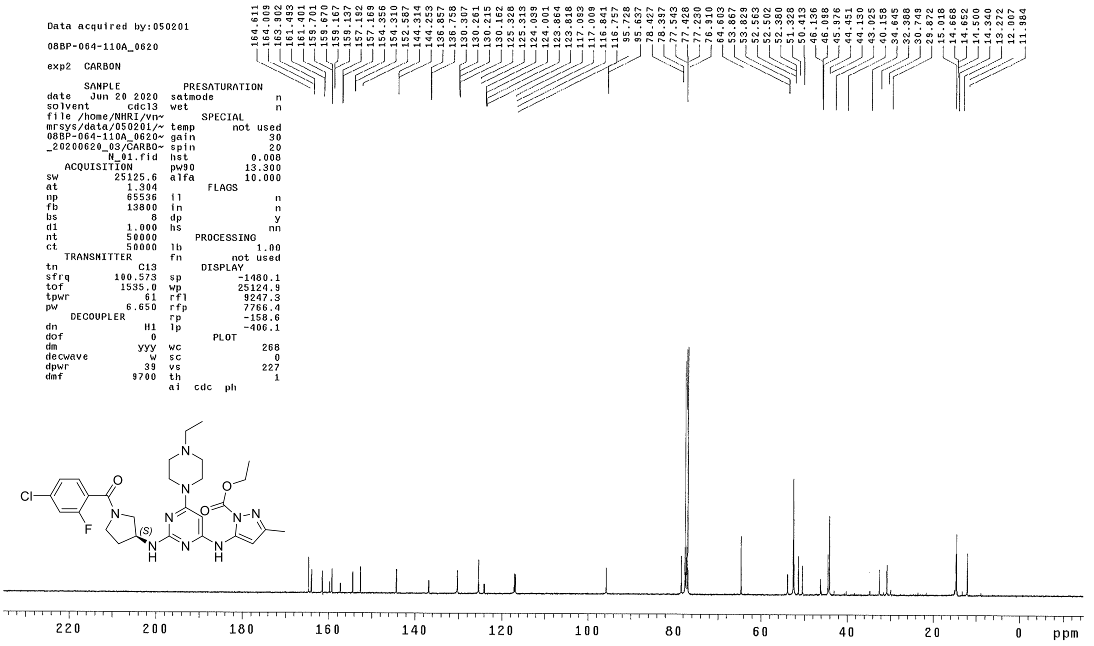

Sample Name: 08BP-064-110A-50 Vial Number: 164
Injection from this vial: 1 of 1 Volume: 20.0 ul
Sample Description:

Chrom Type: Fixed WL Chromatogram, 254 nm

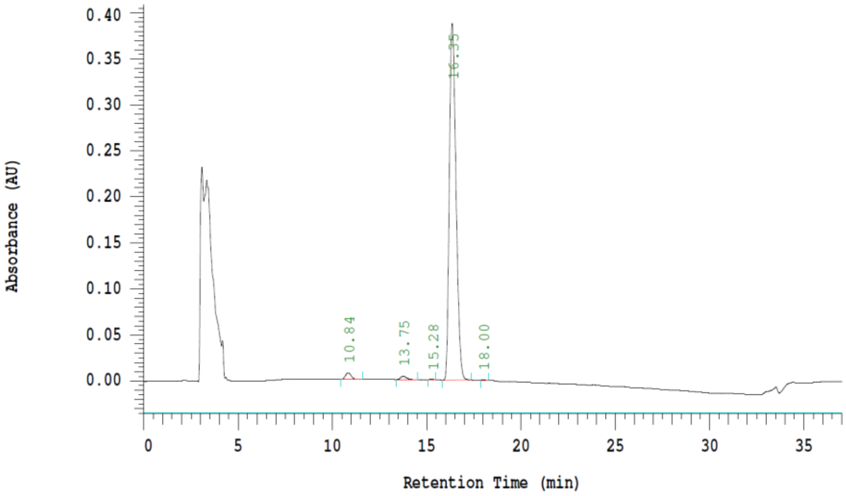

Processing Method: Purity_37min
Column Type: Column Method Developer: Bob
Method Description:

Peak Quantitation: AREA
Calculation Method: AREA%

No.	RT	Area	Height	Conc 1
1	10.84	71168	3494	1.485
2	13.75	42392	1954	0.884
3	15.28	1760	140	0.037
4	16.35	4675766	193693	97.548
5	18.00	2210	184	0.046
		4793296	199465	100.000

Peak rejection level: 0

Figure S25. NMR and HPLC spectra of compound 23.

Ethyl (*S*)-3-((2-((1-(4-chloro-2-fluorobenzoyl)pyrrolidin-3-yl)amino)-6-(4-ethylpiperazin-1-yl)pyrimidin-4-yl)amino)-5-methyl-1H-pyrazole-1-carboxylate (23)

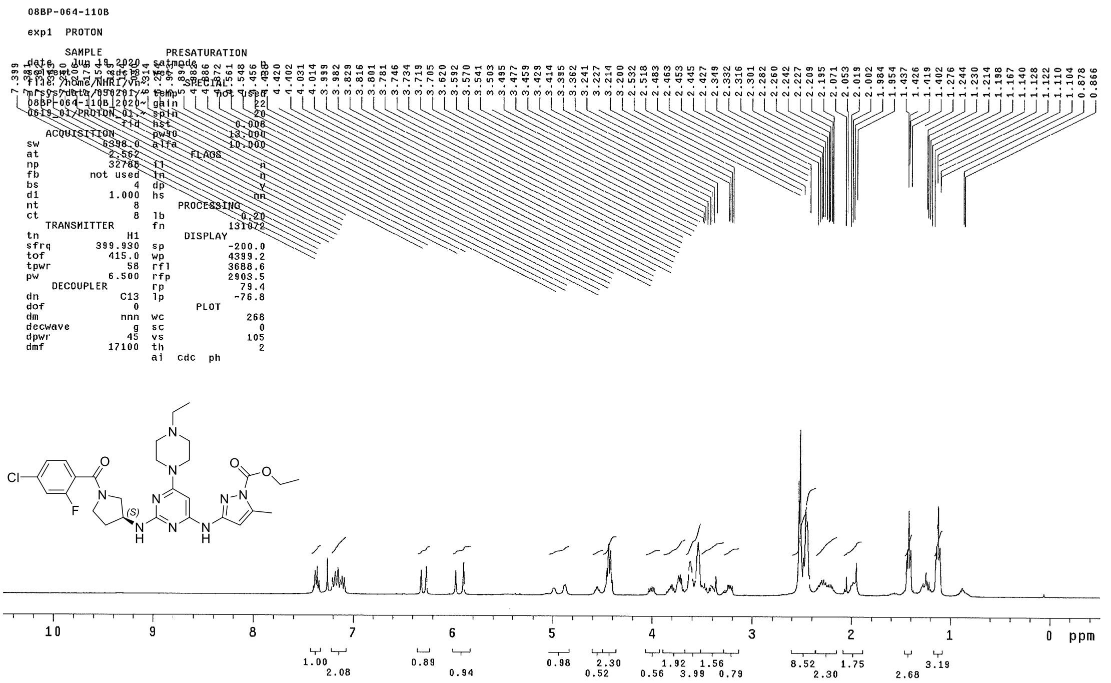

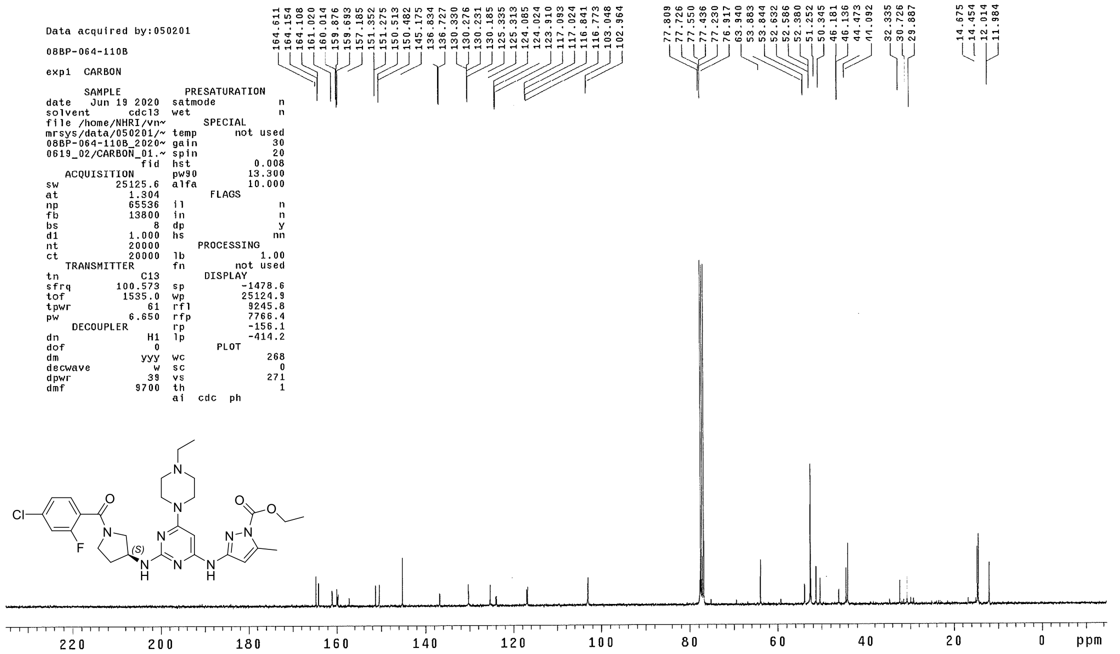

Sample Name: 08BP-064-110B-50
Injection from this vial: 1 of 1
Sample Description:

Vial Number: 76
Volume: 20.0 ul

Chrom Type: Fixed WL Chromatogram, 254 nm

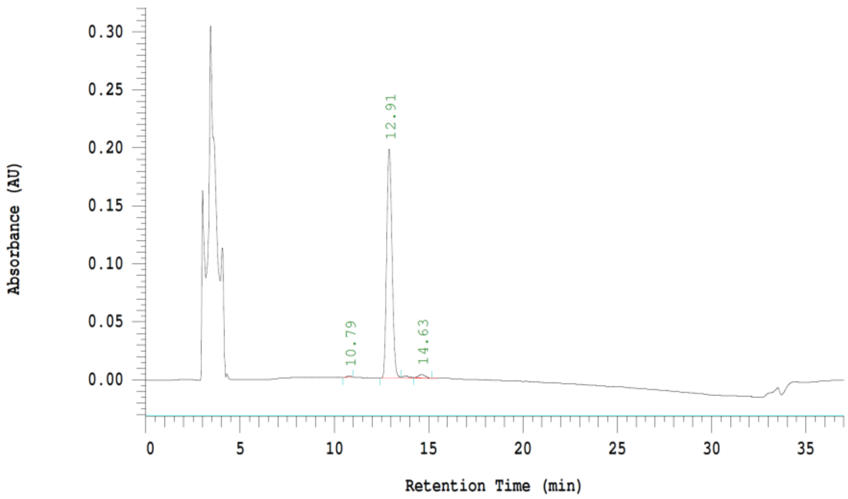

Processing Method: Purity_37min
Column Type: Column
Method Description:

Method Developer: Bob

Peak Quantitation: AREA
Calculation Method: AREA%

No.	RT	Area	Height	Conc 1
1	10.79	7474	435	0.380
2	12.91	1920236	98637	97.667
3	14.63	38385	1542	1.952
		1966095	100614	100.000

Peak rejection level: 0

Figure S26. NMR and HPLC spectra of compound 24.

***tert*-butyl (*S*)-5-((2-((1-(4-chloro-2-fluorobenzoyl)pyrrolidin-3-yl)amino)-6-(4-ethylpiperazin-1-yl)pyrimidin-4-yl)amino)-3-methyl-1H-pyrazole-1-carboxylate (24)**

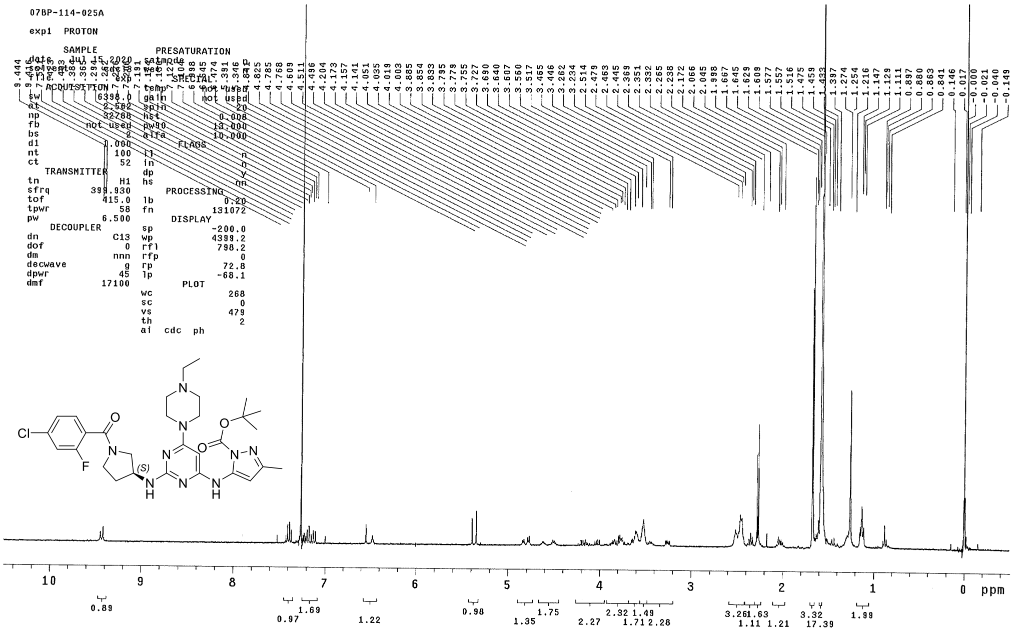

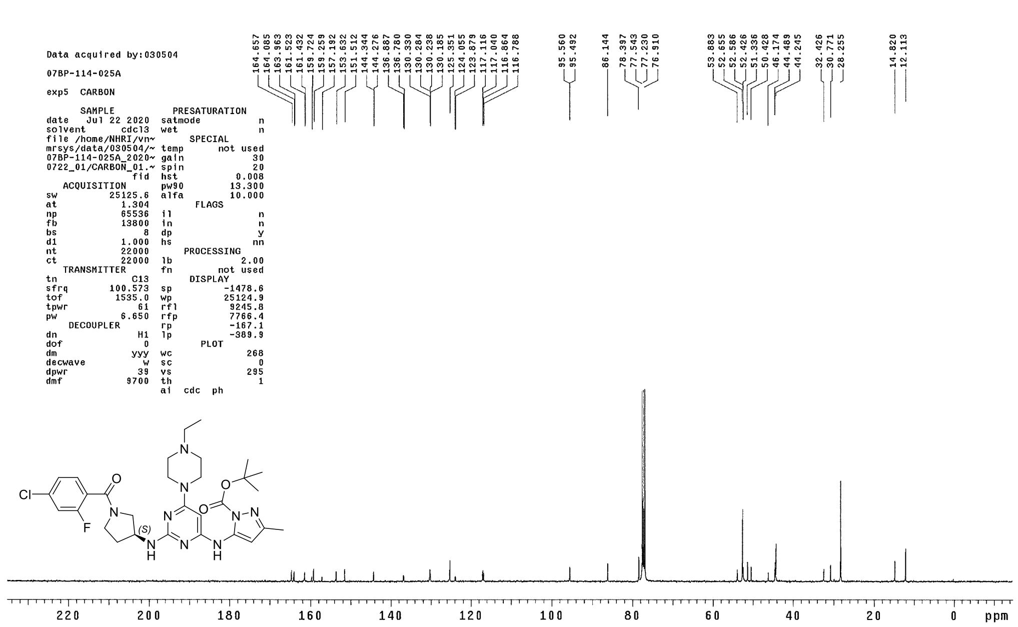

Sample Name: 07BP-114-025A-1-50　　Vial Number: 56
Injection from this vial: 1 of 1　　Volume: 20.0 ul
Sample Description:

Chrom Type: Fixed WL Chromatogram, 254 nm

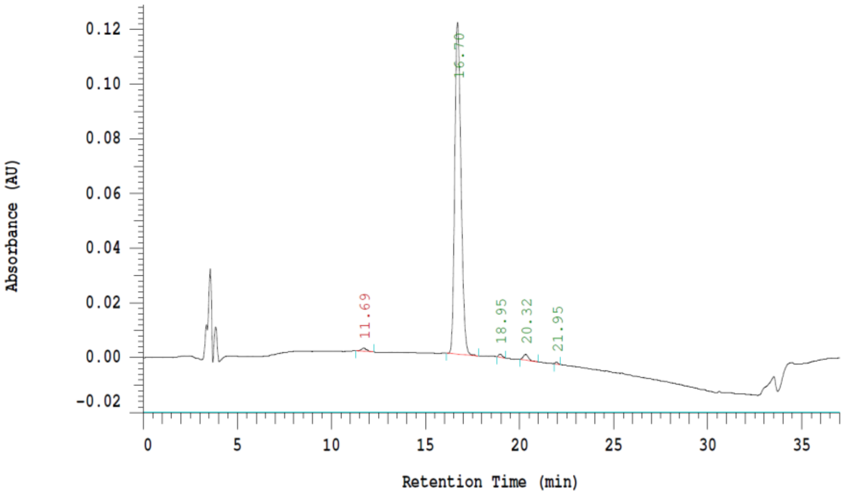

Processing Method: Purity_37min
Column Type: Column　　Method Developer: Bob
Method Description:

Peak Quantitation: AREA
Calculation Method: AREA%

No.	RT	Area	Height	Conc 1
1	11.69	10749	505	0.754
2	16.70	1384707	60567	97.101
3	18.95	7700	598	0.540
4	20.32	19485	1043	1.366
5	21.95	3406	397	0.239
		1426047	63110	100.000

Peak rejection level: 0

Figure S27. NMR and HPLC spectra of compound 25.

(*S*)-1-(5-((2-((1-(4-chloro-2-fluorobenzoyl)pyrrolidin-3-yl)amino)-6-(4-ethylpiperazin-1-yl)pyrimidin-4-yl)amino)-3-methyl-1H-pyrazol-1-yl)propan-1-one (25)

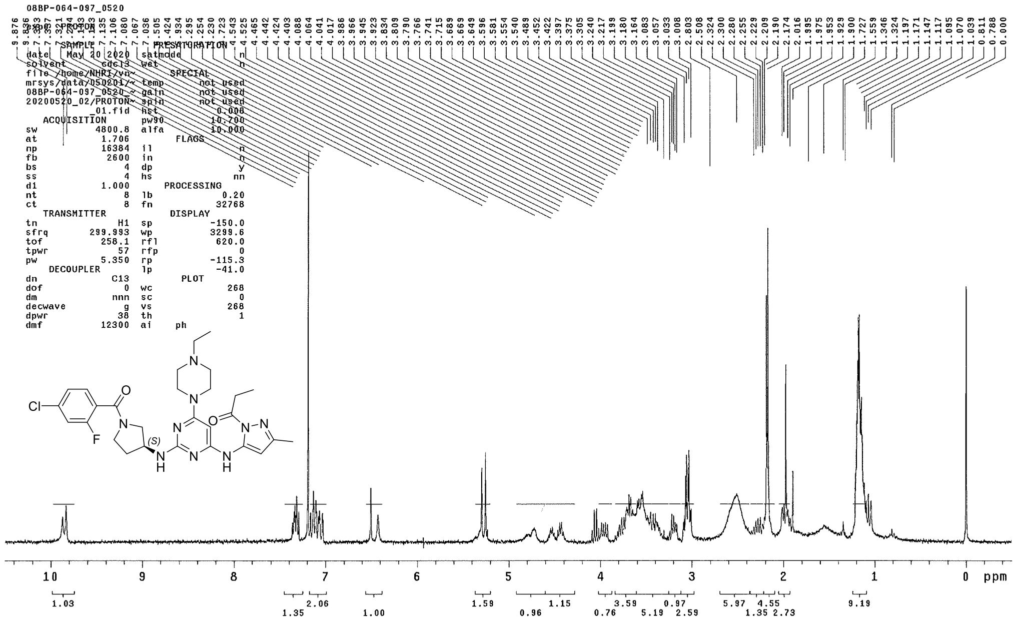

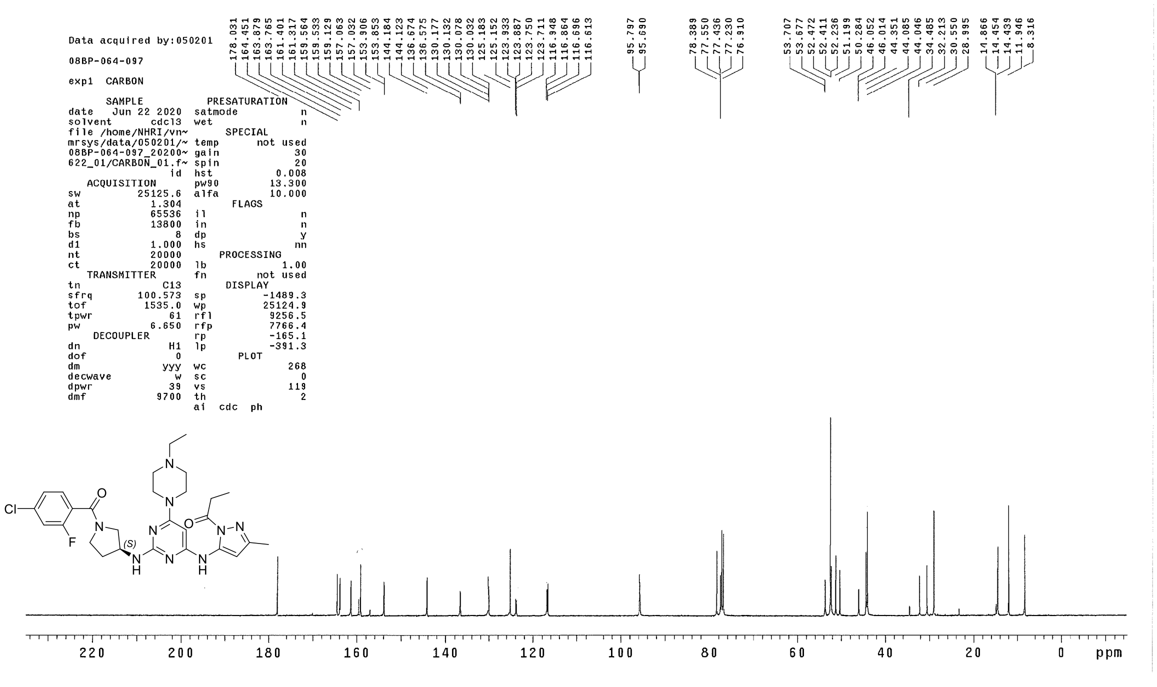

Sample Name: 08BP-064-097A_0630-50 Vial Number: 84
Injection from this vial: 1 of 1 Volume: 20.0 ul
Sample Description:

Chrom Type: Fixed WL Chromatogram, 254 nm

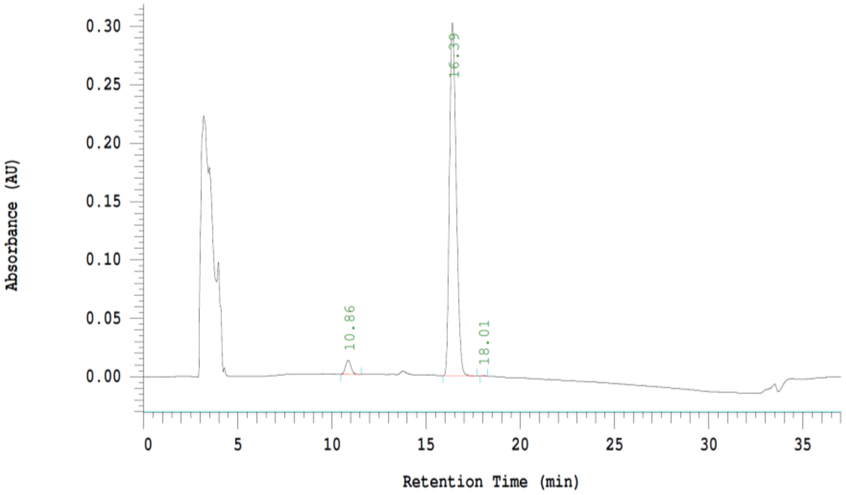

Processing Method: Purity_37min
Column Type: Column Method Developer: Bob
Method Description:

Peak Quantitation: AREA
Calculation Method: AREA%

No.	RT	Area	Height	Conc 1
1	10.86	118935	5933	3.122
2	16.39	3688002	150897	96.812
3	18.01	2522	200	0.066
		3809459	157030	100.000

Peak rejection level: 0

Figure S28. NMR and HPLC spectra of compound 26.

(*S*)-1-(3-((2-((1-(4-chloro-2-fluorobenzoyl)pyrrolidin-3-yl)amino)-6-(4-ethylpiperazin-1-yl)pyrimidin-4-yl)amino)-5-methyl-1H-pyrazol-1-yl)propan-1-one (26)

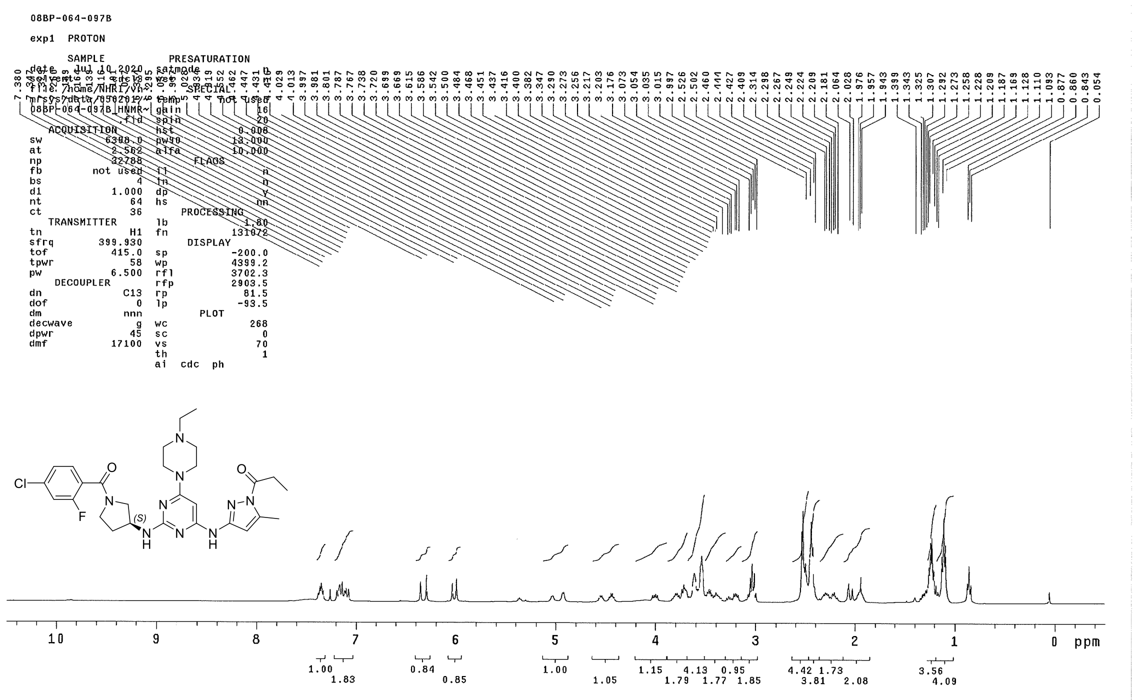

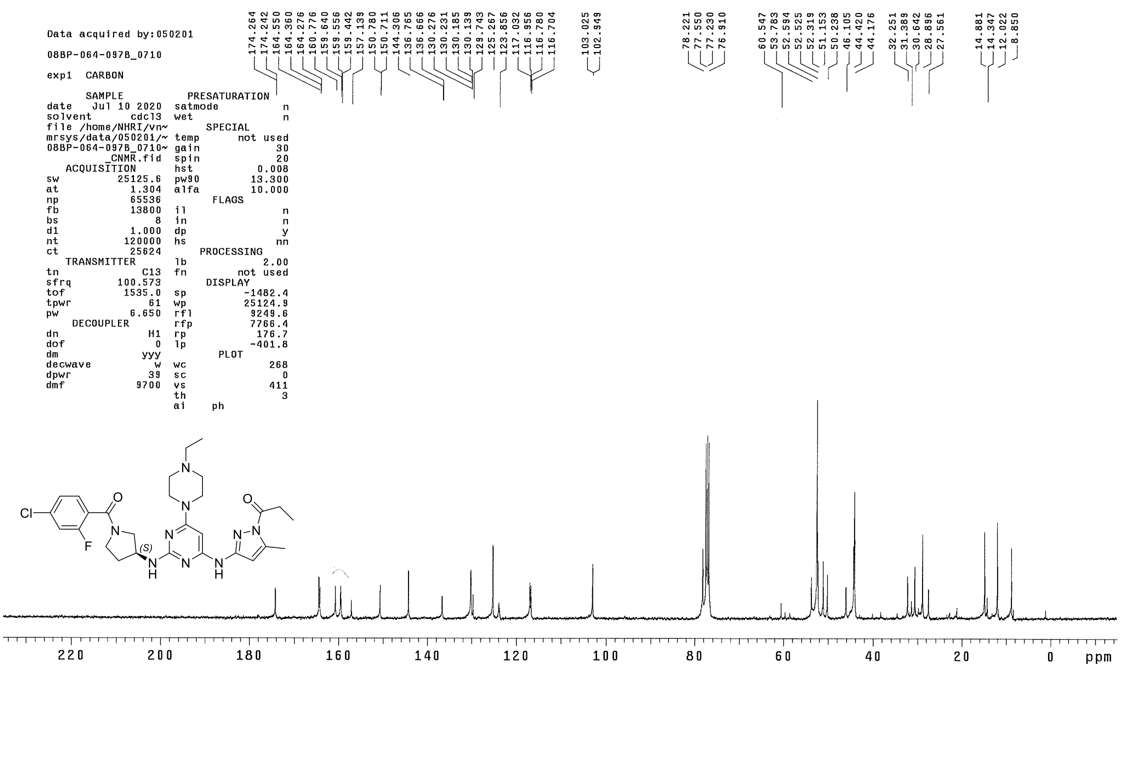

Sample Name: 08BP-064-097B-50 Vial Number: 82
Injection from this vial: 1 of 1 Volume: 20.0 ul
Sample Description:

Chrom Type: Fixed WL Chromatogram, 254 nm

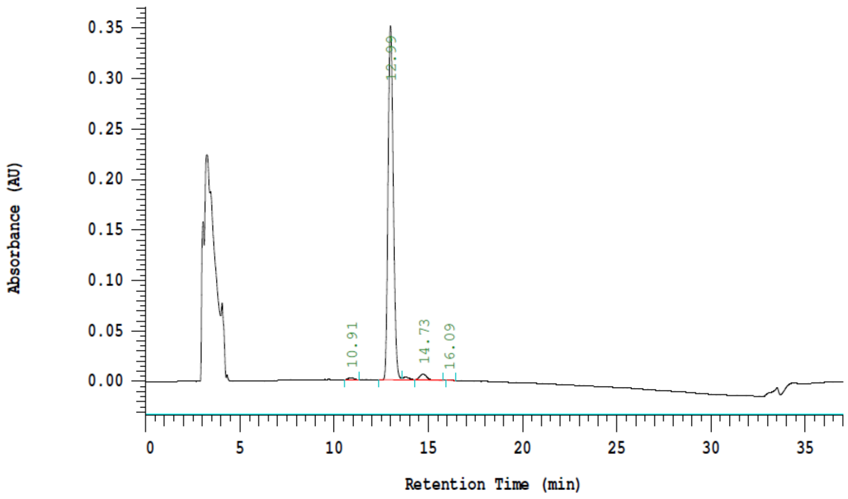

Processing Method: Purity_37min
Column Type: Column Method Developer: Bob
Method Description:

Peak Quantitation: AREA
Calculation Method: AREA%

No.	RT	Area	Height	Conc 1
1	10.91	18112	916	0.527
2	12.99	3342574	175226	97.322
3	14.73	70140	2817	2.042
4	16.09	3723	215	0.108
		3434549	179174	100.000

Peak rejection level: 0

Figure S29. NMR and HPLC spectra of compound 27.

(*S*)-1-(5-((2-((1-(4-chloro-2-fluorobenzoyl)pyrrolidin-3-yl)amino)-6-(4-ethylpiperazin-1-yl)pyrimidin-4-yl)amino)-3-methyl-1H-pyrazol-1-yl)-2,2-dimethylpropan-1-one (27)

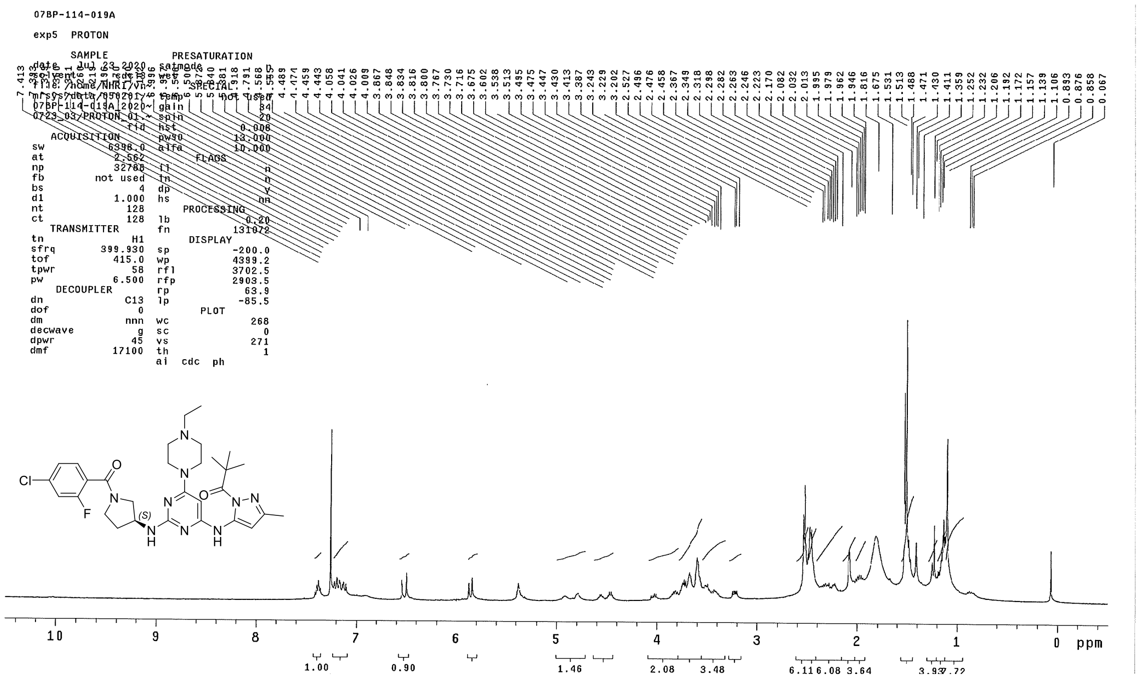

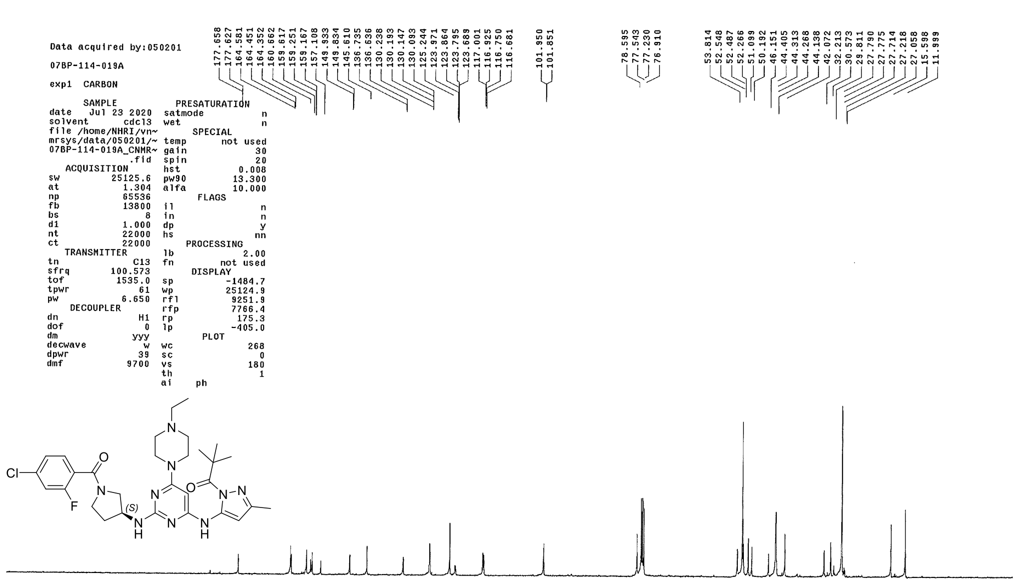

Sample Name: 07BP-114-019A-50
Injection from this vial: 1 of 1
Sample Description:

Vial Number: 52
Volume: 20.0 ul

Chrom Type: Fixed WL Chromatogram, 254 nm

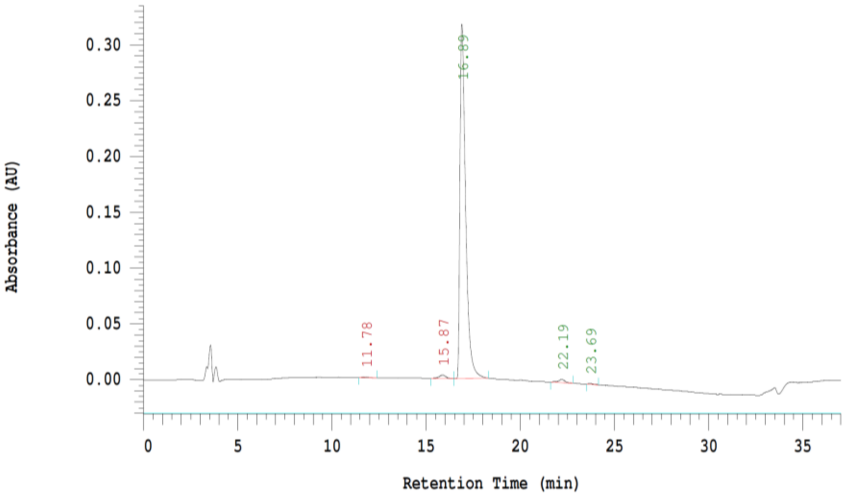

Processing Method: Purity_37min
Column Type: Column
Method Description:

Method Developer: Bob

Peak Quantitation: AREA
Calculation Method: AREA%

No.	RT	Area	Height	Conc 1
1	11.78	5807	275	0.173
2	15.87	37714	1492	1.123
3	16.89	3273011	158605	97.451
4	22.19	35921	1444	1.070
5	23.69	6180	353	0.184
		3358633	162169	100.000

Peak rejection level: 0

Figure S30. ^{1}H NMR spectrum of compound 29.

2,6-dichloro-N-(5-methyl-1H-pyrazol-3-yl)pyrimidin-4-amine (29)

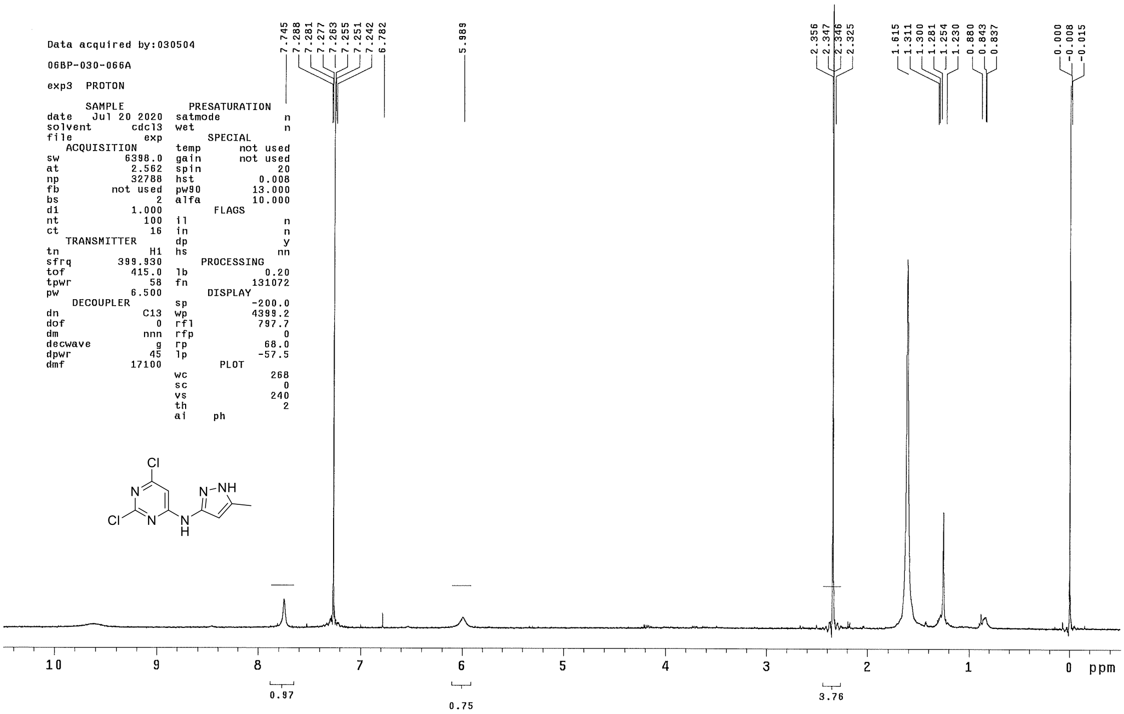

Figure S31. ^{1}H NMR spectrum of compound 30.

(*S*)-(3-chloro-2-fluorophenyl)(3-((4-chloro-6-((5-methyl-1H-pyrazol-3-yl)amino)pyrimidin-2-yl)amino)pyrrolidin-1-yl)methanone (30)

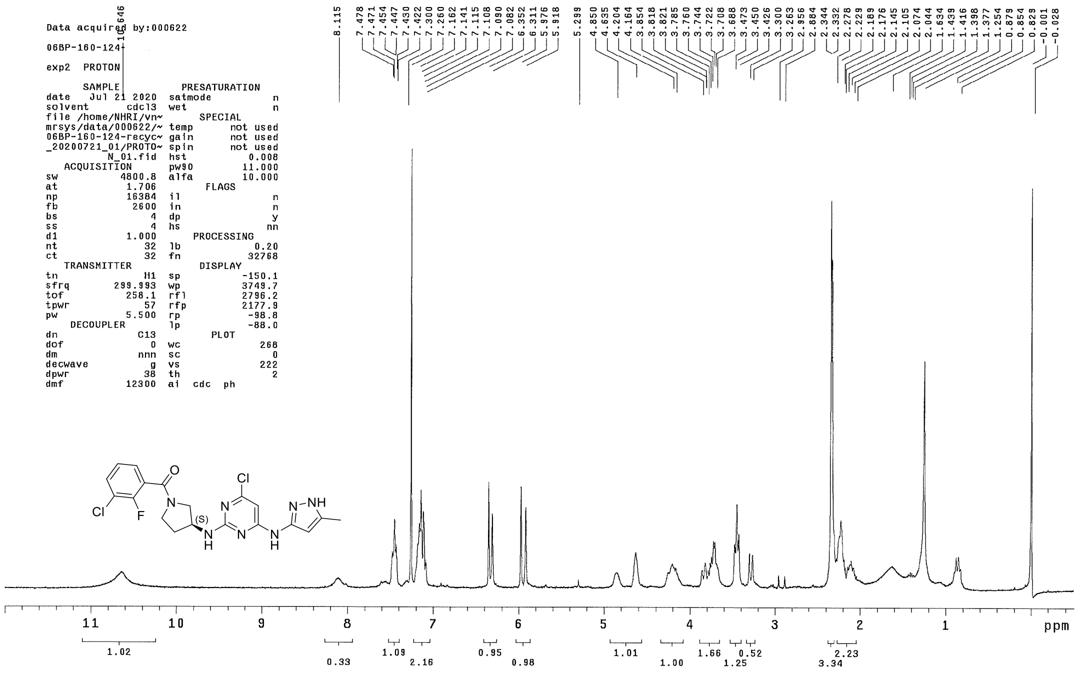

Figure S32. ^{1}H NMR spectrum of compound 32.

***tert*-butyl (*S*)-3-((4,6-dichloropyrimidin-2-yl)amino)pyrrolidine-1-carboxylate (32)**

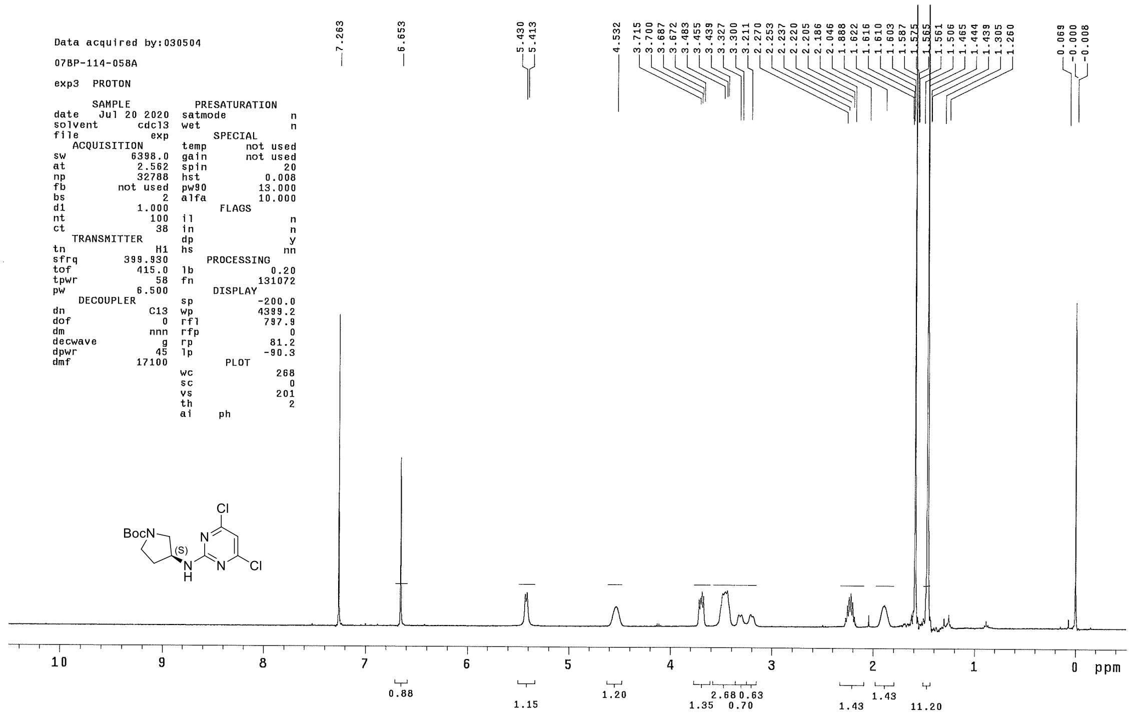

Figure S33. ^{1}H NMR spectrum of compound 33.

***tert*-butyl (*S*)-3-((4-chloro-6-((5-methyl-1H-pyrazol-3-yl)amino)pyrimidin-2-yl)amino)pyrrolidine-1-carboxylate (33)**

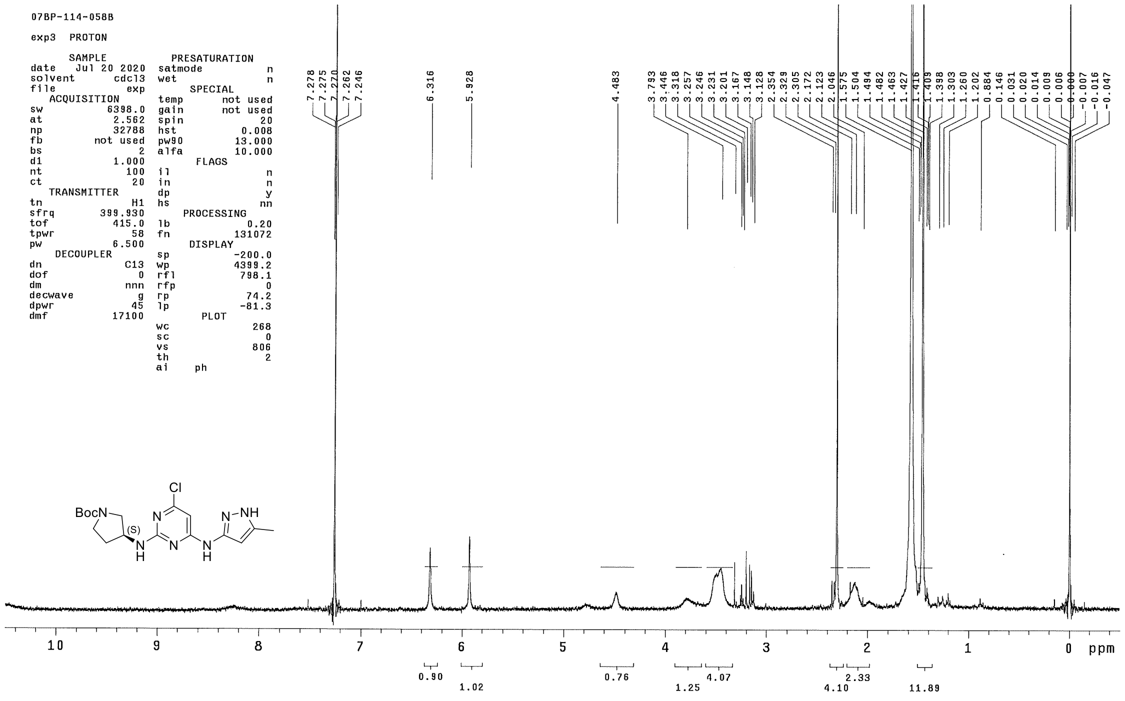

Figure S34. ^{1}H NMR spectrum of compound 34.

***tert*-butyl (*S*)-3-((4-(4-ethylpiperazin-1-yl)-6-((5-methyl-1H-pyrazol-3-yl)amino)pyrimidin-2-yl)amino)pyrrolidine-1-carboxylate (34)**

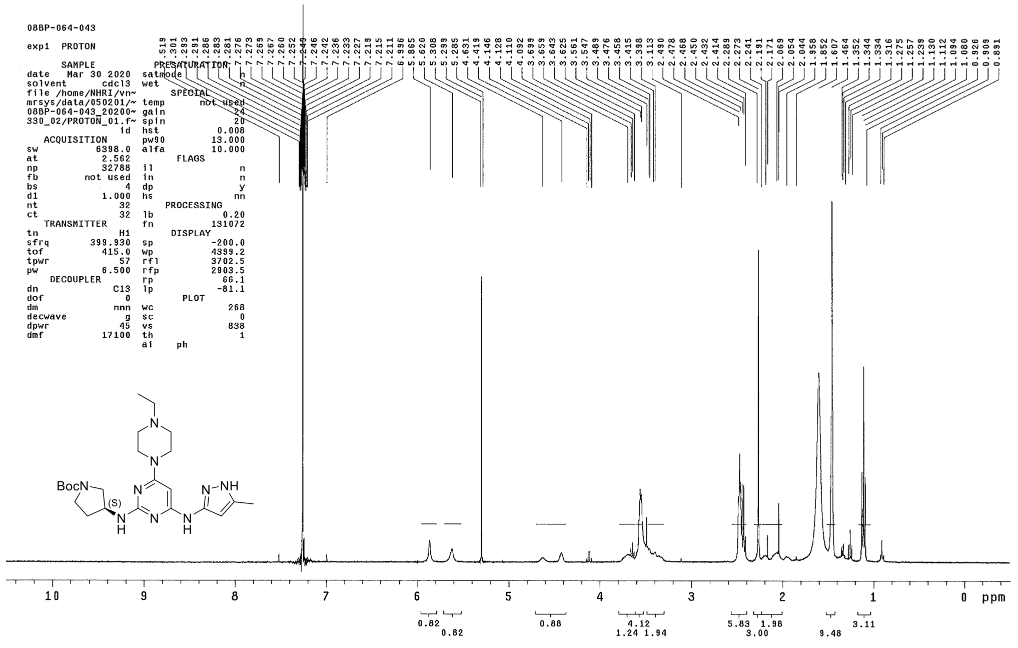

Supplement: Supplementary file 2 — jm0c01806_si_002.pdf [file jm0c01806_si_002.pdf]
